# Supplementary material for: Synthesis of inter-[60]fullerene conjugates with inherent chirality
Source: Nat Commun. 2024 Jan 15;15:514. doi: 10.1038/s41467-024-44834-x (PMC10789730; doi:10.1038/s41467-024-44834-x)
Supplement: Supplementary file 1 — Supplementary Information [file 41467_2024_44834_MOESM1_ESM.pdf]

Supplementary Information

Synthesis of inter-[60]fullerene conjugates with inherent chirality

Yoshifumi Hashikawa,* Shu Okamoto, and Yasujiro Murata*

Institute for Chemical Research, Kyoto University, Uji, Kyoto 611-0011, Japan
E-mail: hashi@scl.kyoto-u.ac.jp
yasujiro@scl.kyoto-u.ac.jp

Contents

Supplementary Methods 3

Supplementary Note 1. Synthetic Trials for Inter-[60]Fullerene Hybrids 3

Supplementary Note 2. Cyclic Voltammograms 14

Supplementary Note 3. UV-Vis-NIR Spectra 15

Supplementary Note 4. IR Spectra 16

Supplementary Note 5. Association Constant 19

Supplementary Note 6. Chirality 21

Supplementary Note 7. Single Crystal X-Ray Structures 24

Supplementary Note 8. DFT Calculations 30

Supplementary References 63

Supplementary Methods

Supplementary Note 1. Synthetic Trials for Inter-[60]Fullerene Hybrids

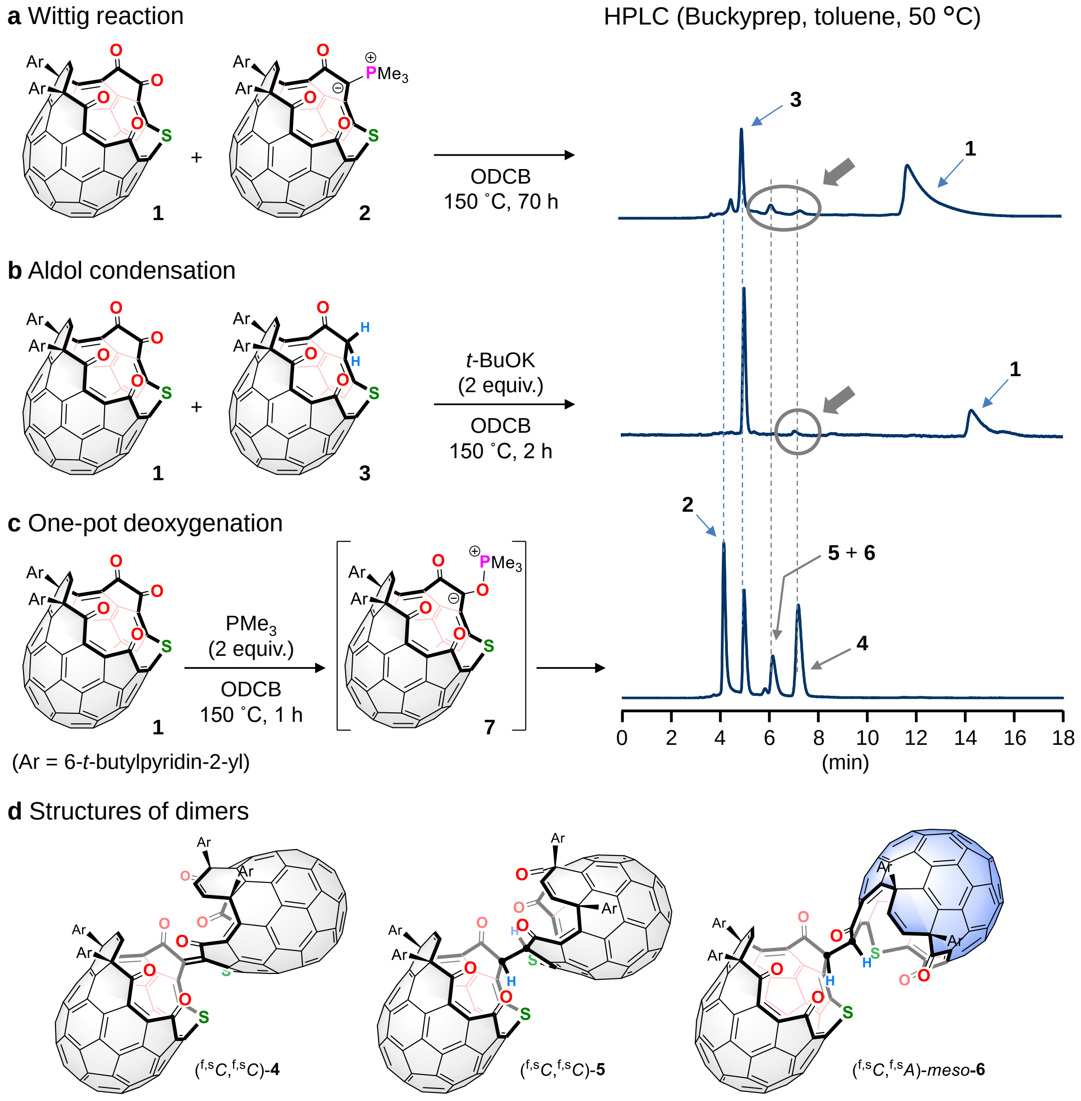

Supplementary Figure 1. Synthetic trials of open-[60]fullerene dimers and HPLC charts of the crude mixtures. a. Wittig reaction using **1** (5.00 mg, 4.41 µmol) and **2** (5.27 mg, 4.41 µmol, 1.0 equiv.) in ODCB (0.50 mL). **b.** Aldol condensation using **1** (5.14 mg, 4.53 µmol), **3** (5.04 mg, 4.50 µmol, 1.0 equiv.), and *t*-BuOK (0.8 mg, 7 µmol, 2 equiv.) in ODCB (0.50 mL). **c.** One-pot deoxygenation using **1** (20.0 mg, 17.6 µmol) and trimethylphosphine (1.0 M in toluene, 35.4 µL, 35 µmol, 2.0 equiv.) in ODCB (2.0 mL, 8.8 mM). **d.** Structures of dimers. The retention time of **1** depends upon a sample concentration.

4: IR (KBr) *ν* 1736, 1742 (C=O) cm^{-1}; ^{1}H NMR (500 MHz, CS_2) *δ* 8.03–8.09 (m, 4H), 7.73–7.76 (m, 4H), 7.64 (d, *J* = 7.4 Hz, 2H), 7.54 (d, *J* = 7.4 Hz, 2H), 7.31–7.36 (m, 4H), 1.52 (s, 18H), 1.42 (s, 18H), –11.11 (s, 2.46H for encapsulated H_2O); ^{13}C NMR (201 MHz, ODCB-d_4) *δ* 189.88, 189.81, 189.72, 183.67, 181.84, 181.75, 168.54, 168.38, 168.15, 167.87, 167.83, 164.31, 164.20, 164.15, 164.08, 155.48, 152.46, 152.39, 151.43, 151.31, 151.02, 150.63, 150.46, 150.30, 150.24, 150.12, 150.06, 149.85, 149.80, 149.56, 149.52, 149.50, 149.48, 149.37, 149.23, 149.14, 149.10, 148.98, 148.84, 148.74, 148.44, 147.10, 146.95, 145.31, 145.13, 144.92, 144.86, 144.69, 144.61, 144.54, 144.47, 144.40, 144.02, 143.89, 143.76, 143.65, 143.50, 143.38, 143.34, 143.14, 142.86, 142.70, 141.75, 141.59, 141.49, 141.33, 140.72, 140.65, 140.55, 140.28, 139.58, 139.54, 139.46, 139.37, 139.32, 139.21, 139.03, 138.71, 138.28, 137.85, 137.79, 137.74, 137.47, 137.36, 137.35, 137.33, 137.31, 137.20, 137.12, 136.87, 136.59, 136.48, 136.36, 136.16, 135.89, 135.83, 135.78, 135.49, 134.17, 133.79, 133.47, 132.87, 132.73, 132.51, 131.95, 131.84, 131.69, 131.25, 131.07, 130.52, 130.40, 130.37, 130.25, 130.21, 129.92, 129.80, 129.71, 129.67, 129.53, 129.32, 129.29, 128.40, 127.53, 126.86, 126.81, 126.44, 124.98, 124.11, 121.03, 119.92, 117.21, 117.00, 116.96, 58.62, 54.57, 54.54, 54.50, 37.61, 37.54, 37.49, 30.06, 29.99, 29.96, 29.91, 29.83. (The carbon signals of **4** and H_2O@**4** were separately observed. The sum of carbon signals for each molecule must be 78 in theory (156 for two components). Observed 145. The 11 sp^2 carbon signals are overlapped.); HRMS (APCI) *m*/*z*: $[M]^{•+}$ Calcd for $C_{164}H_{52}N_4O_6S_2$ (**4**) 2236.3323; Found 2236.3428.

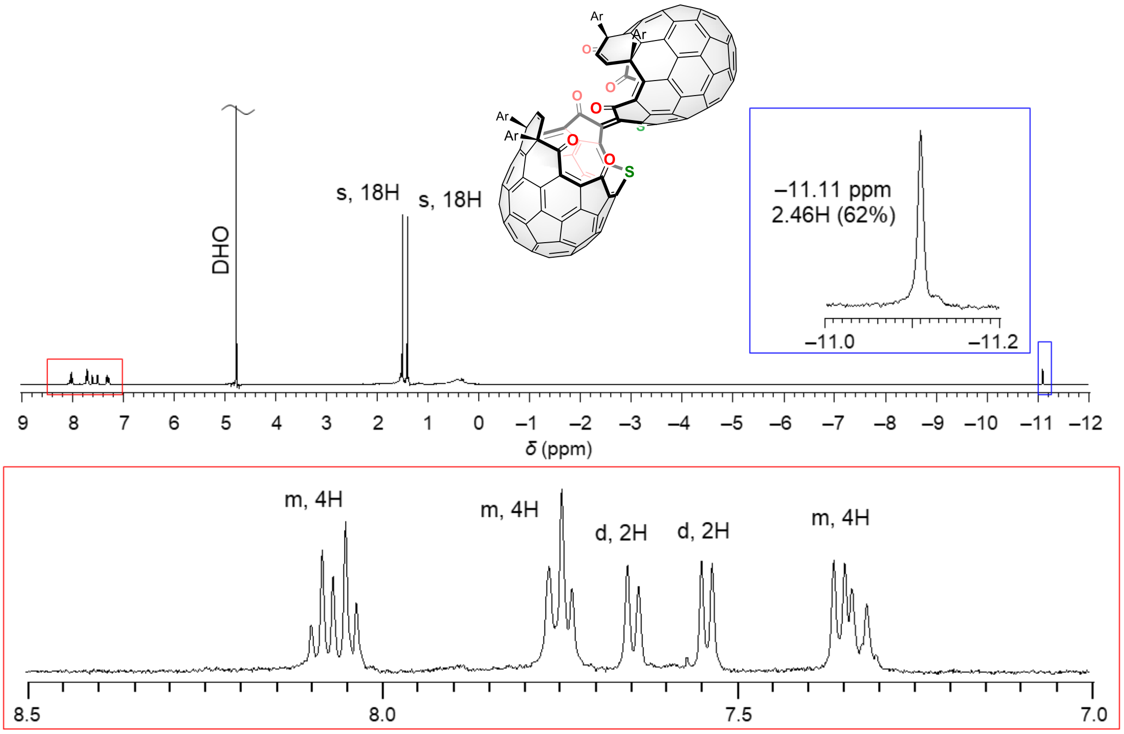

Supplementary Figure 2. ^{1}H NMR spectra (500 MHz, CS_2, NMR lock was established by D_2O filled in a glass capillary) of **4**.

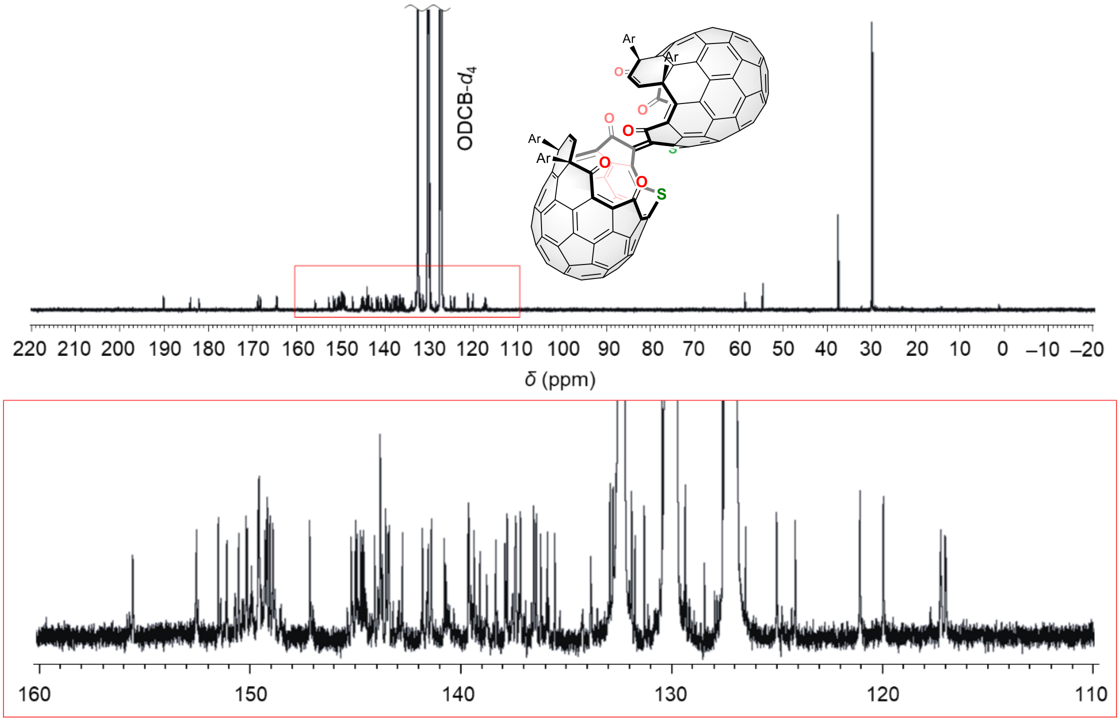

Supplementary Figure 3. ^{13}C NMR spectra (201 MHz, ODCB-d_4) of **4**.

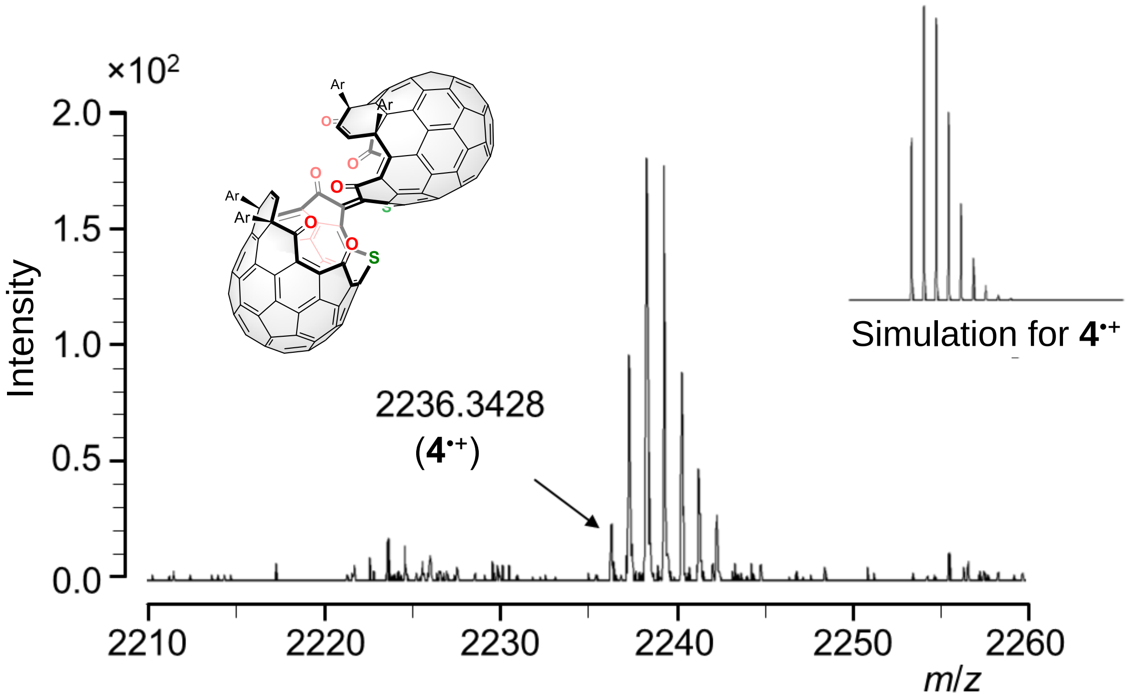

Supplementary Figure 4. APCI mass spectra (positive ion mode) of **4**. The signals corresponding to **4**$^{•+}$ and [**4**+H]$^{+}$ were overlapped.

($^{f,s}C$,$^{f,s}C$)-**5**: IR (KBr) *ν* 1733 (C=O) cm^{-1}; ^{1}H NMR (500 MHz, ODCB-d_4) *δ* 7.39 (t, *J* = 7.7 Hz, 2H), 7.30 (t, *J* = 8.0 Hz, 2H), 7.17–7.19 (m, 4H), 7.02–7.05 (m, 4H), 6.87 (d, *J* = 8.0 Hz, 2H), 6.53 (m, 2H), 5.15–5.20 (m, 2H), 1.15 (s, 18H), 1.06 (s, 18H), –11.46 (s, 2.04H for encapsulated H_2O); ^{13}C NMR (201 MHz, ODCB-d_4) *δ* 199.99, 191.28, 191.21, 191.17, 184.11, 184.04, 183.89, 183.88, 168.22, 168.20, 168.02, 167.98, 164.26, 164.14, 164.08, 163.38, 163.33, 163.23, 155.41, 155.39, 155.23, 155.21, 152.23, 152.13, 151.37, 151.28, 151.04, 150.88, 150.53, 150.49, 150.48, 150.35, 150.28, 150.23, 150.02, 149.98, 149.86, 149.78, 149.71, 149.50, 149.46, 149.28, 149.25, 149.15, 149.03, 148.97, 148.94, 148.91, 148.88, 148.77, 148.71, 148.57, 148.43, 148.37, 147.20, 147.10, 147.00, 146.94, 146.83, 145.84, 145.61, 145.30, 145.14, 145.05, 144.77, 144.71, 144.69, 144.65, 144.55, 144.50, 144.44, 144.39, 144.32, 144.20, 144.08, 144.04, 143.85, 143.83, 143.79, 143.69, 143.60, 143.50, 143.44, 143.29, 143.26, 143.19, 143.15, 143.12, 143.05, 143.03, 142.93, 141.28, 141.13, 140.92, 140.77, 140.58, 140.50, 140.37, 140.32, 139.53, 139.46, 139.36, 139.32, 139.22, 138.87, 138.82, 138.66, 138.62, 138.47, 138.31, 138.28, 138.26, 138.24, 138.09, 138.01, 137.67, 137.61, 137.57, 137.45, 137.24, 137.22, 137.18, 137.09, 137.07, 136.98, 136.90, 136.87, 136.63, 136.58, 136.53, 136.41, 136.39, 136.28, 136.21, 136.12, 136.10, 135.90, 135.82, 135.58, 135.55, 135.34, 133.89, 133.20, 132.88, , 132.24, 132.03, 131.98, 131.94, 131.89, 131.77, 131.74, 131.21, 130.92, 130.82, 130.77, 130.75, 130.41, 130.40, 130.37, 130.37, 129.53, 129.38, 129.25, 129.13, 127.92, 127.80, 127.68, 127.54, 127.53, 126.81, 126.78, 124.97, 124.91, 124.76, 124.66, 120.19, 120.17, 119.92, 117.05, 117.04, 117.02, 117.00, 59.05, 59.03, 58.98, 55.45, 55.41, 54.38, 54.36, 54.36, 37.44, 37.41, 37.41, 29.80, 29.73 (The carbon signals of **5**, H_2O@**5**, and other encapsulated species (N_2 or Ar) were separately observed. The sum of carbon signals for each molecule must be 78 in theory (234 for three components). Observed 195. One carbonyl, 30 sp^2, and 8 sp^3 carbon signals are overlapped.); HRMS (APCI) *m*/*z*: [M]$^{\bullet +}$ Calcd for $C_{164}H_{54}N_4O_6S_2$ (**5**) 2238.3479; Found 2238.3383.

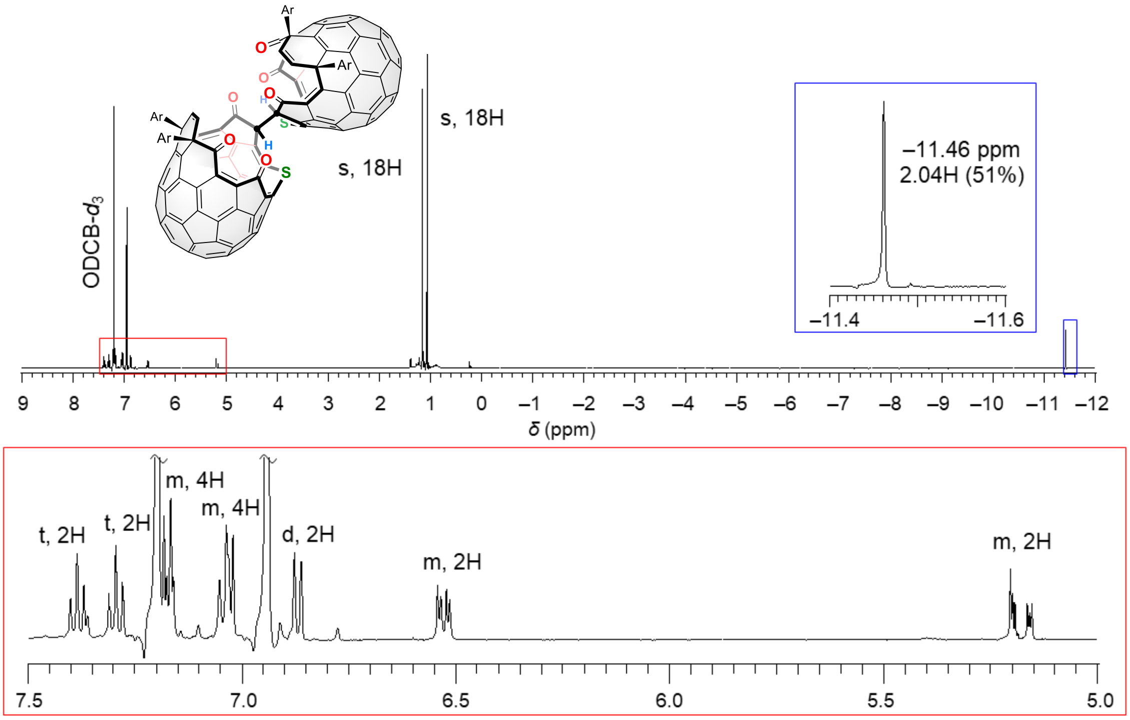

Supplementary Figure 5. ^{1}H NMR spectra (500 MHz, ODCB-d_4) of ($^{f,s}C$,$^{f,s}C$)-**5**.

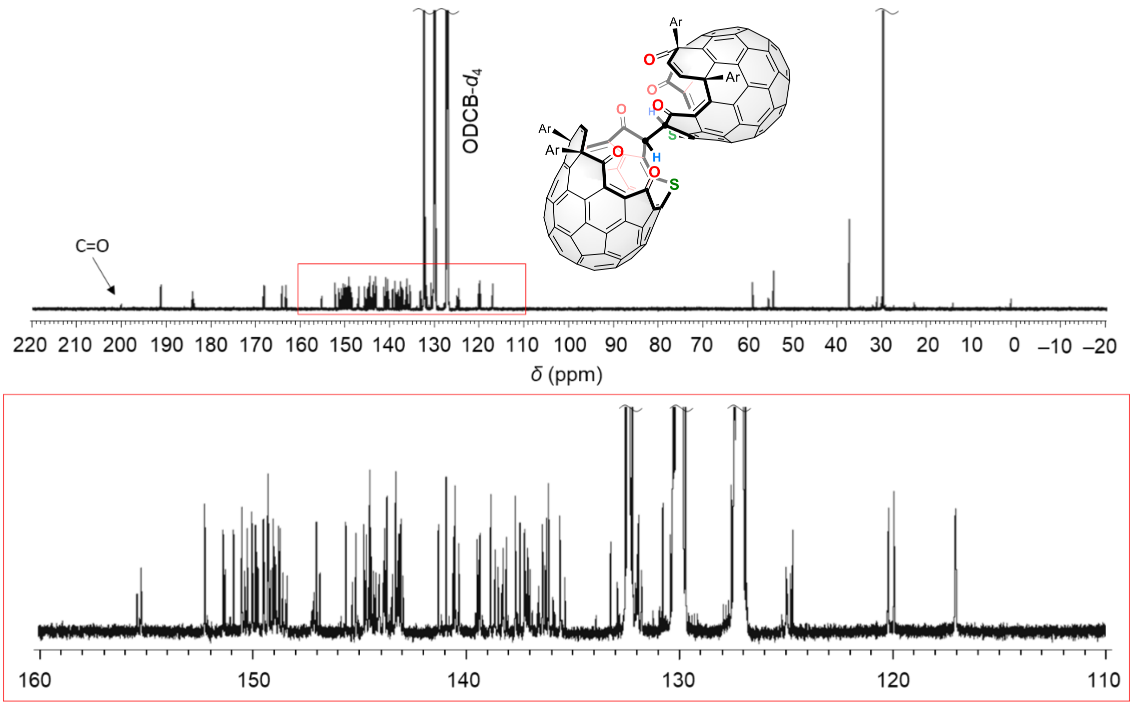

Supplementary Figure 6. ^{13}C NMR spectra (201 MHz, ODCB-d_4) of ($^{f,s}C$,$^{f,s}C$)-**5**.

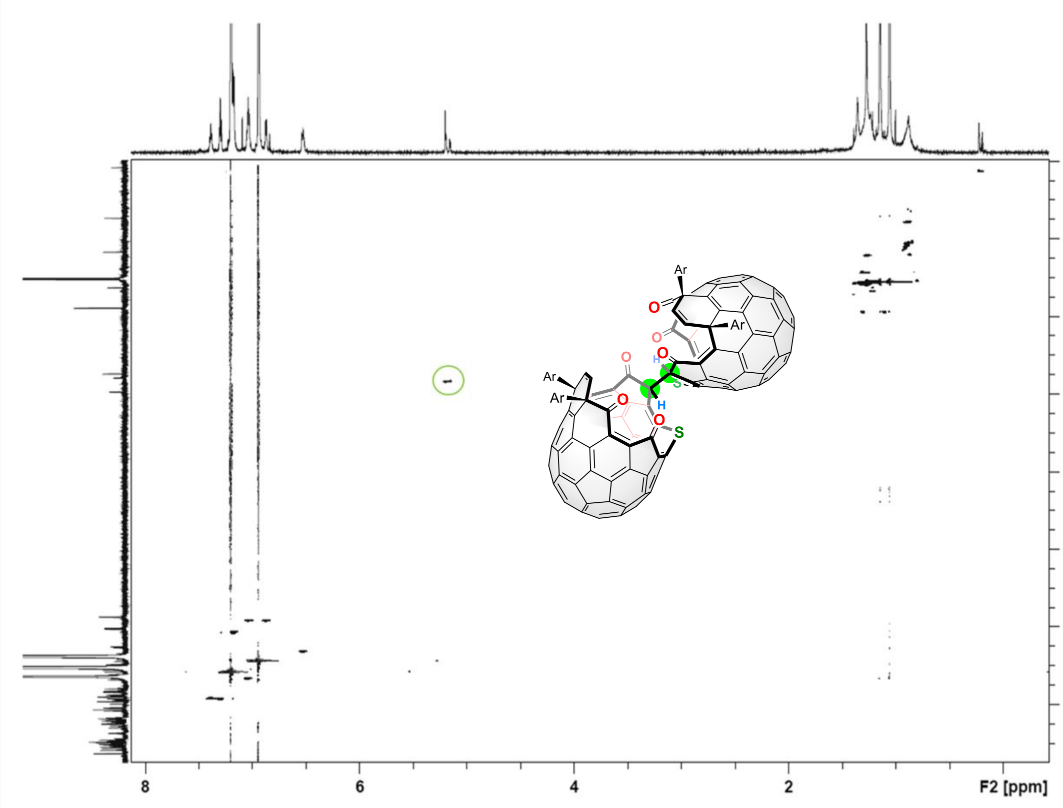

Supplementary Figure 7. HMQC spectrum (800 MHz, ODCB-d_4) of *rac*-**5**.

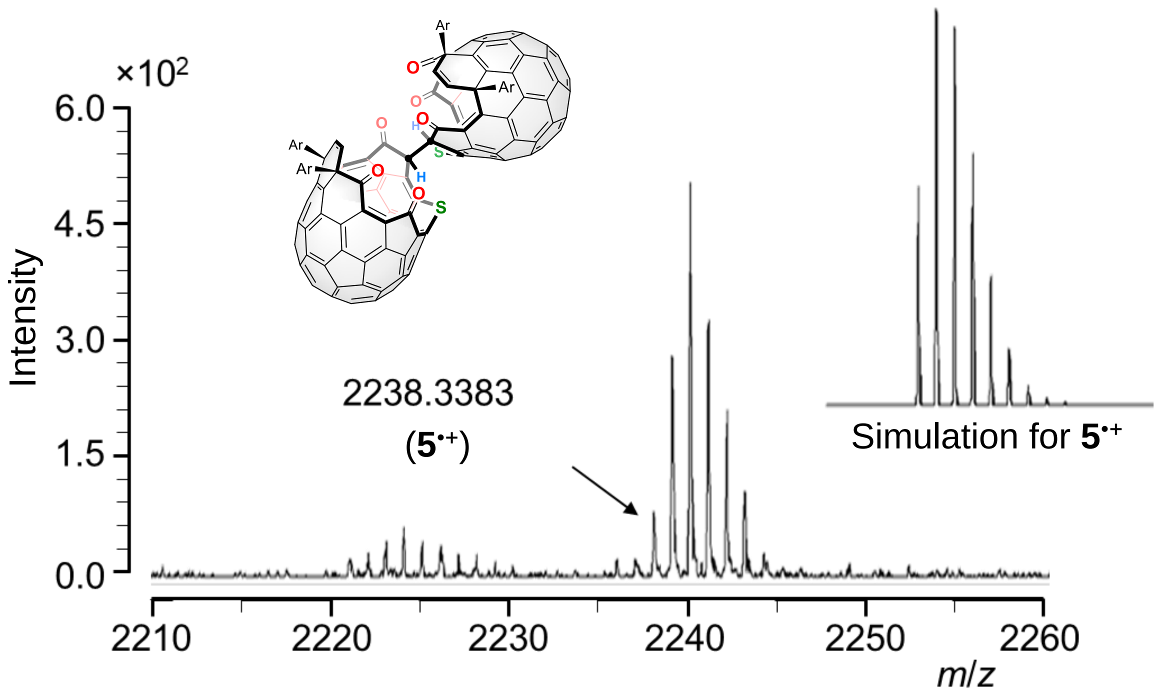

Supplementary Figure 8. APCI mass spectra (positive ion mode) of *rac*-**5**. The signals corresponding to $\mathbf{5}^{\bullet+}$ and $[\mathbf{5}+\mathrm{H}]^+$ were overlapped.

6: IR (KBr) *ν* 1734 (C=O) cm^{-1}; ^{1}H NMR (500 MHz, CS_2) δ 7.94 (t, *J* = 8.0 Hz, 1H), 7.80 (t, *J* = 8.0 Hz, 2H), 7.71 (d, *J* = 8.0 Hz, 2H), 7.54 (d, *J* = 8.0 Hz, 2H), 7.49 (d, *J* = 8.0 Hz, 2H), 7.40 (d, *J* = 8.0 Hz, 2H), 7.33–7.35 (m, 2H), 6.90–6.94 (m, 2H), 5.26–5.34 (m, 2H), 1.51 (s, 18H), 1.47 (s, 18H), –11.15 (s, 2.84H for encapsulated H_2O); ^{13}C NMR (201 MHz, ODCB-d_4) δ 196.87, 196.84, 196.77, 196.75, 190.99, 190.94, 190.87, 190.82, 183.45, 183.40, 183.40, 183.25, 168.20, 168.19, 168.03, 168.00, 163.97, 163.96, 163.90, 163.44, 163.43, 163.37, 163.36, 163.27, 163.26, 155.47, 155.45, 155.43, 152.20, 152.19, 152.09, 151.24, 151.16, 151.01, 150.81, 150.69, 150.46, 150.43, 150.33, 150.24, 150.18, 150.11, 150.07, 150.01, 149.87, 149.84, 149.80, 149.77, 149.73, 149.66, 149.52, 149.46, 149.42, 149.30, 149.26, 149.23, 149.22, 149.18, 149.05, 148.99, 148.94, 148.91, 148.90, 148.77, 148.70, 148.68, 148.59, 148.56, 148.44, 148.38, 148.37, 146.96, 146.90, 146.80, 146.45, 146.02, 145.72, 145.66, 145.53, 145.51, 145.11, 145.10, 145.00, 144.96, 144.85, 144.69, 144.67, 144.64, 144.62, 144.58, 144.54, 144.52, 144.45, 144.35, 143.81, 143.67, 143.49, 143.17, 143.09, 142.96, 142.89, 142.81, 141.52, 141.51, 141.41, 141.20, 141.06, 140.89, 140.68, 140.47, 140.34, 140.32, 140.25, 140.19, 139.88, 139.64, 139.60, 139.26, 139.20, 139.14, 139.02, 138.97, 138.95, 138.93, 138.92, 138.88, 138.85, 138.66, 138.63, 138.49, 138.01, 137.70, 137.56, 137.31, 137.30, 137.22, 137.15, 137.12, 137.06, 136.79, 136.76, 136.63, 136.56, 136.48, 136.38, 136.34, 136.27, 136.18, 136.16, 135.66, 135.50, 135.08, 135.06, 135.00, 133.57, 133.36, 132.91, 132.11, 131.97, 131.87, 131.82, 131.43, 131.25, 130.95, 130.77, 130.59, 130.41, 130.37, 129.38, 129.29, 129.25, 129.13, 128.39, 127.53, 126.81, 126.44, 125.27, 125.06, 124.86, 124.73, 120.38, 120.31, 119.96, 117.10, 117.07, 117.03, 58.99, 58.97, 58.96, 58.92, 55.34, 55.31, 55.27, 55.25, 54.46, 54.43, 54.40, 37.51, 37.50, 37.49, 37.47, 37.46, 37.45, 29.97, 29.90, 29.82 (The carbon signals of **6**, H_2O@**6**, and other encapsulated species (N_2 and Ar) were separately observed. The sum of carbon signals for each molecule must be 78 in theory (312 for four components). Observed 206. The 98 sp^2 and 8 sp^3 carbon signals are overlapped.); HRMS (APCI) *m*/*z*: $[M]^{\bullet+}$ Calcd for $C_{164}H_{54}N_4O_6S_2$ (**6**) 2238.3479; Found 2238.3430.

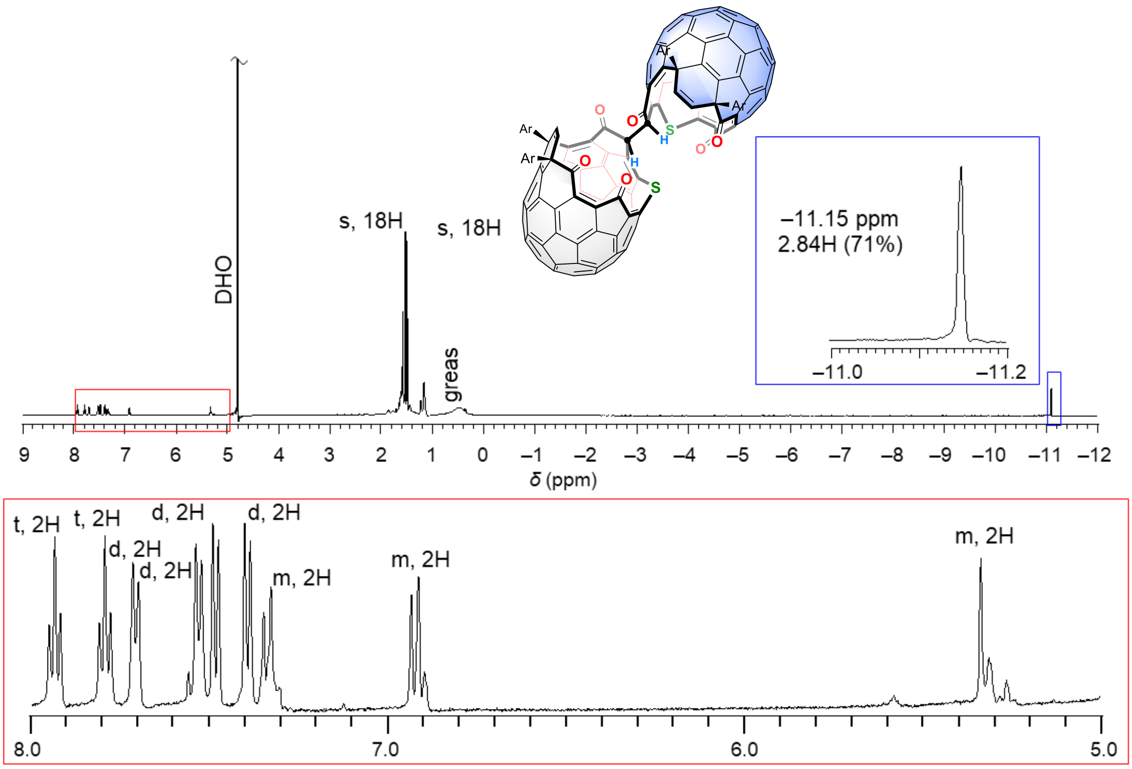

Supplementary Figure 9. ^{1}H NMR spectra (500 MHz, CS_2, NMR lock was established by D_2O filled in a glass capillary) of **6**.

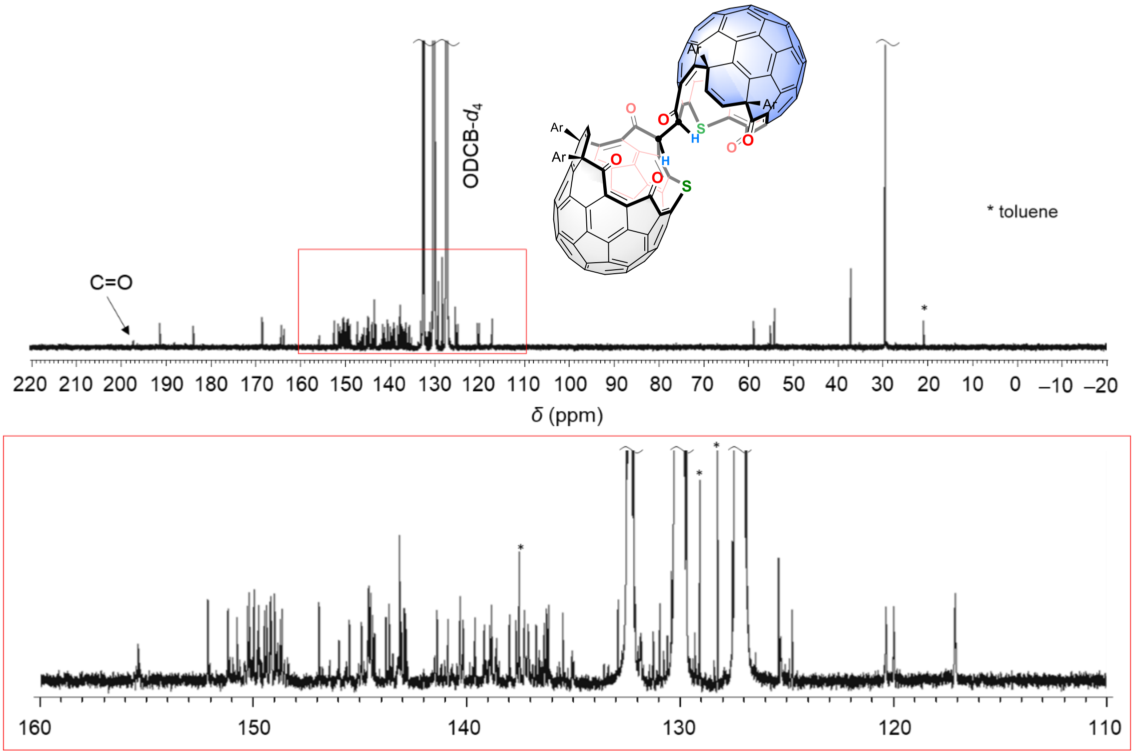

Supplementary Figure 10. ^{13}C NMR spectra (201 MHz, ODCB-d_4) of **6**.

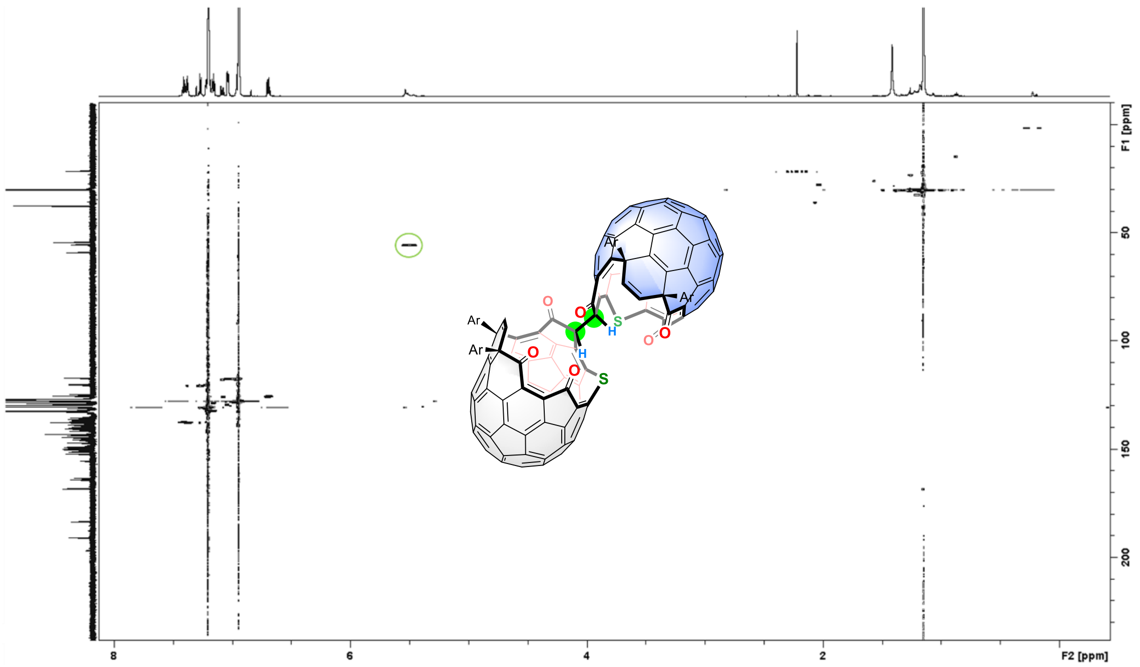

Supplementary Figure 11. HMQC spectrum (800 MHz, ODCB-d_4) of **6**.

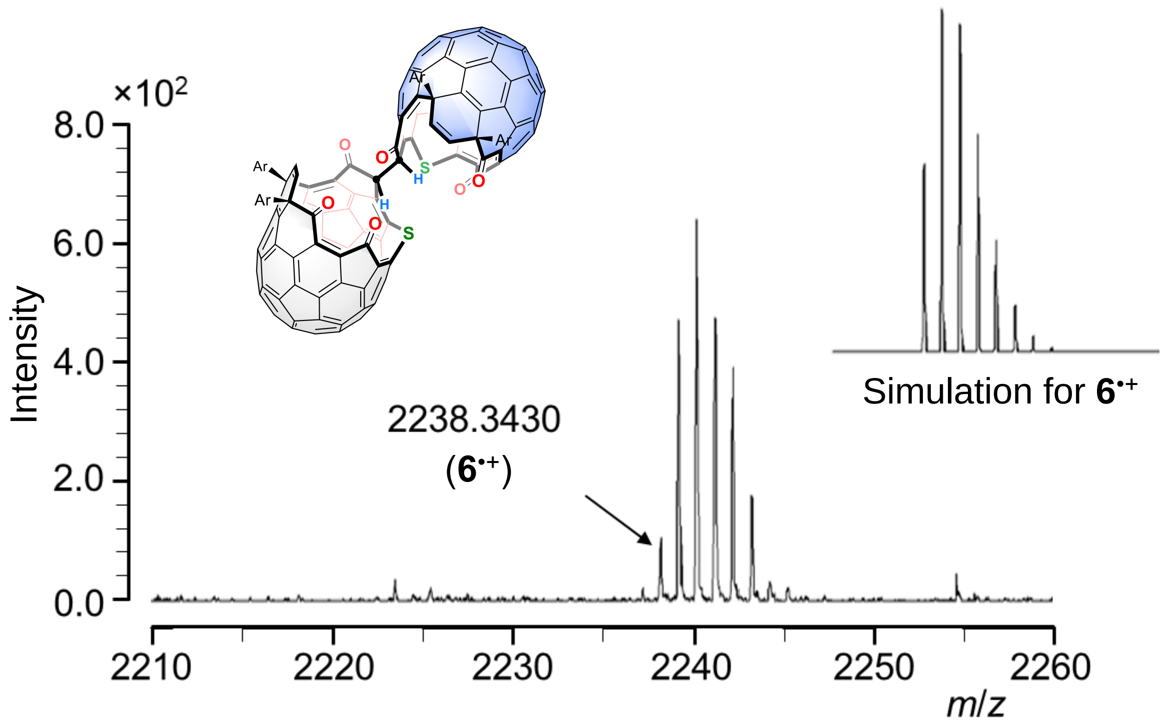

Supplementary Figure 12. APCI mass spectra (positive ion mode) of **6**.

2: ^{1}H NMR (500 MHz, $CDCl_3$) δ 7.55 (t, J = 7.8 Hz, 1H), 7.41 (t, J = 7.9 Hz, 1H), 7.36 (d, J = 7.8 Hz, 1H), 7.22–7.24 (m, 2H), 7.14 (d, J = 7.8 Hz, 1H), 7.04 (d, J = 7.9 Hz, 1H), 6.49 (d, J = 10.3 Hz, 1H), 2.46 (d, $^2J_{HP}$ = 13.7 Hz, 9H), 1.15 (s, 9H), 1.09 (s, 9H), –10.94 (s, 1.39H for encapsulated H_2O); ^{31}P NMR (202 MHz, $CDCl_3$) δ 7.16 (H_2O@**2**); HRMS (APCI) *m*/*z*: $[M]^{\bullet-}$ Calcd for $C_{85}H_{35}N_2O_3PS$ (**2**) 1194.2111; Found 1194.2130. (These data matched well with the reported ones[1].)

3: ^{1}H NMR (500 MHz, $CDCl_3$) δ 7.58 (t, J = 7.9 Hz, 1H), 7.47 (t, J = 7.9 Hz, 1H), 7.19 (d, J = 7.9 Hz, 1H), 7.16 (d, J = 7.9 Hz, 1H), 7.08 (d, J = 7.9 Hz, 1H), 7.02 (d, J = 10.3 Hz, 1H), 6.54 (d, J = 10.3 Hz, 1H), 4.08 (d, J = 21.8 Hz, 1H), 4.00 (d, J = 21.8 Hz, 1H), 1.18 (s, 9H), 1.08 (s, 9H), –11.47 (s, 1.43H for encapsulated H_2O) (One sp^2 signal is overlapped with the solvent peak.); HRMS (APCI) *m*/*z*: $[M]^{\bullet-}$ Calcd for $C_{82}H_{28}N_2O_3S$ (**3**) 1120.1826; Found 1120.1833. (These data matched well with the reported ones[1].)

Supplementary Note 2. Cyclic Voltammograms

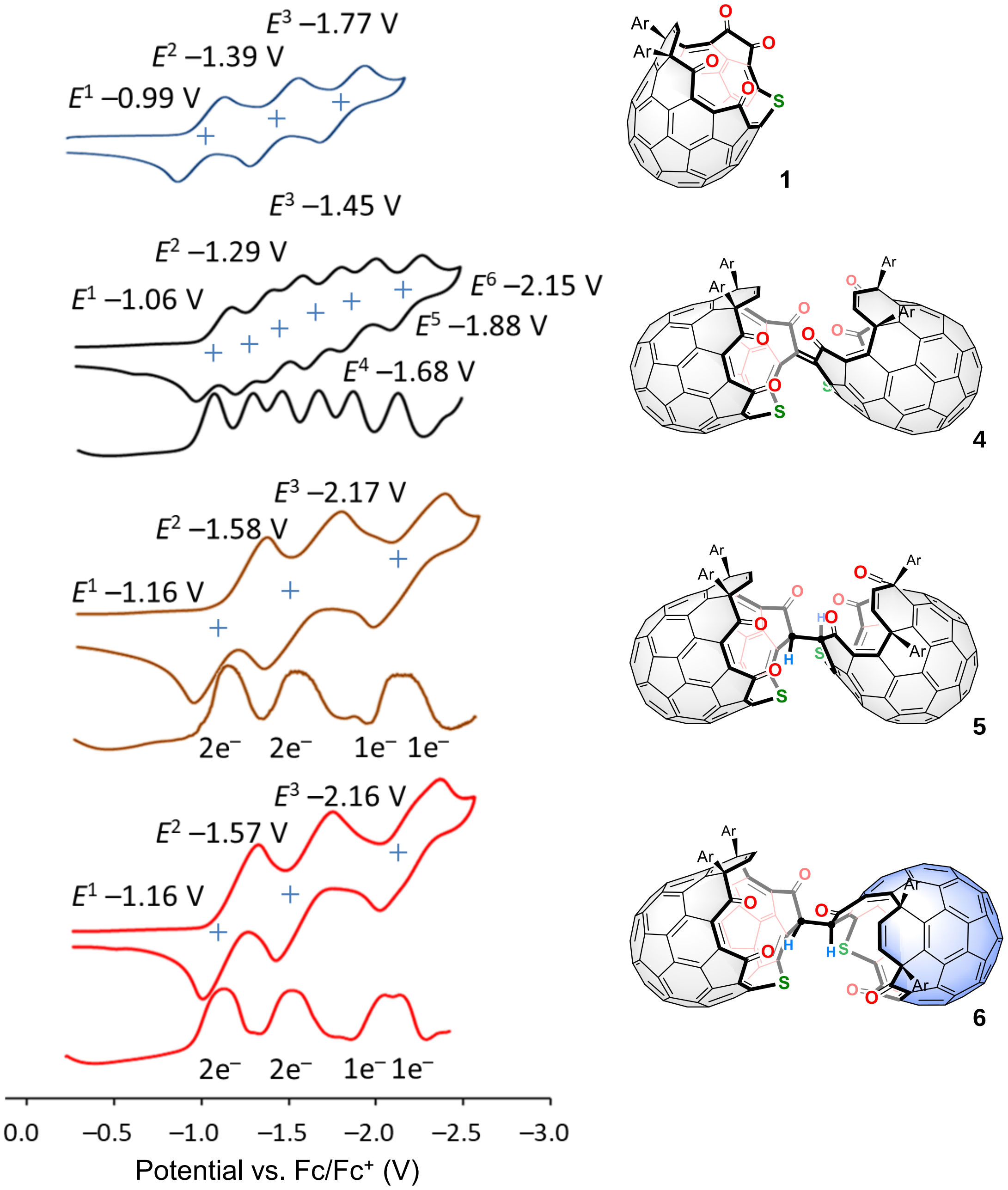

Supplementary Figure 13. Cyclic and differential pulse voltammograms of monomer **1** and dimers **4**–**6** (Ar = 6-*t*-butylpyridin-2-yl). Sample concentrations was set to 0.50 mM in ODCB except for **1** (1.0 mM). As a supporting electrolyte, *n*-$Bu_4N•BF_4$ (0.10 M) was used. The scan rate was set to 100 mV s^{-1}.

Supplementary Note 3. UV-Vis-NIR Spectra

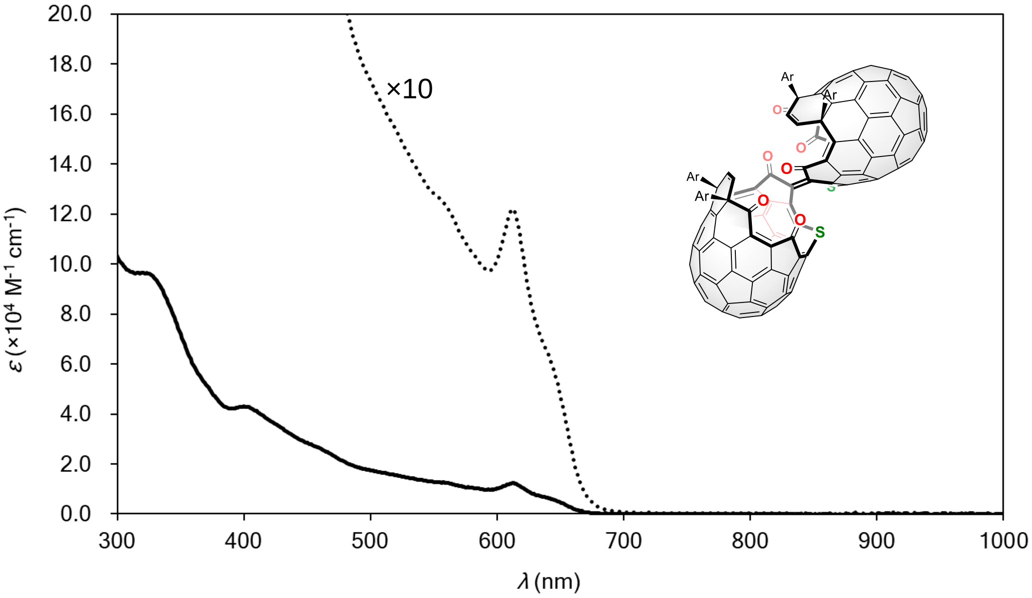

Supplementary Figure 14. UV-vis-NIR absorption spectrum of **4** (50 μM in toluene).

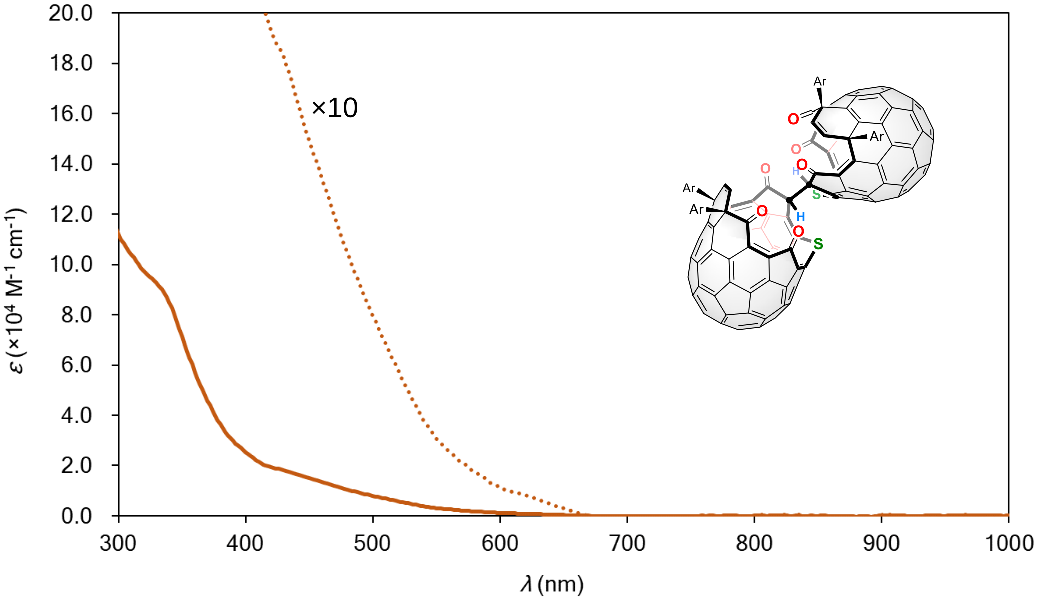

Supplementary Figure 15. UV-vis-NIR absorption spectrum of ($^{f,s}C$,$^{f,s}C$)-**5** (10 μM in toluene). Since *rac*-**5** was hardly dissolved in organic solvents due to self-association, the spectrum was recorded for enantiomerically pure ($^{f,s}C$,$^{f,s}C$)-**5**.

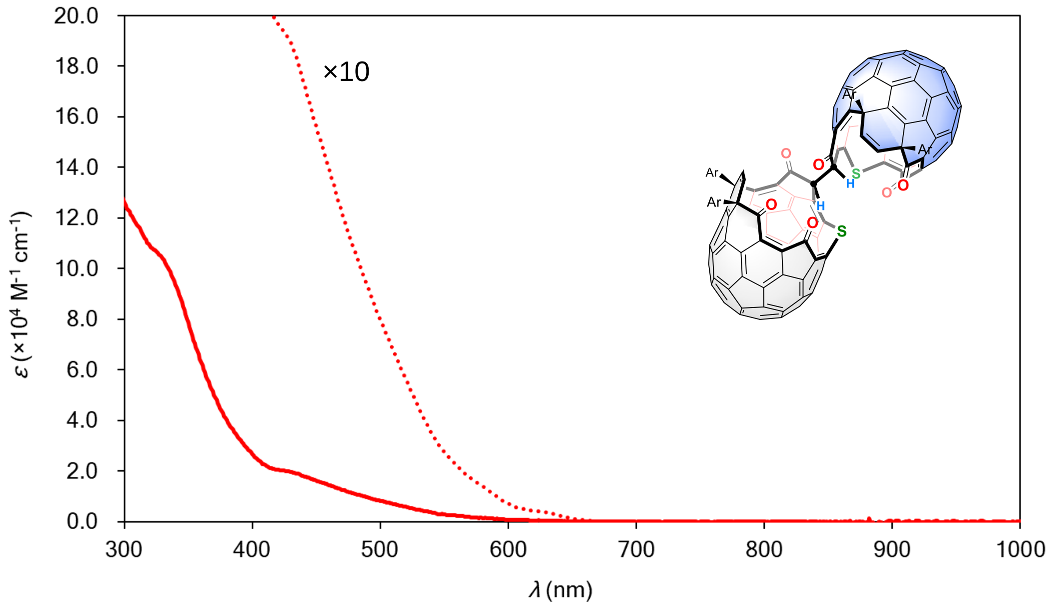

Supplementary Figure 16. UV-vis-NIR absorption spectrum of **6** (50 μM in toluene).

Supplementary Note 4. IR Spectra

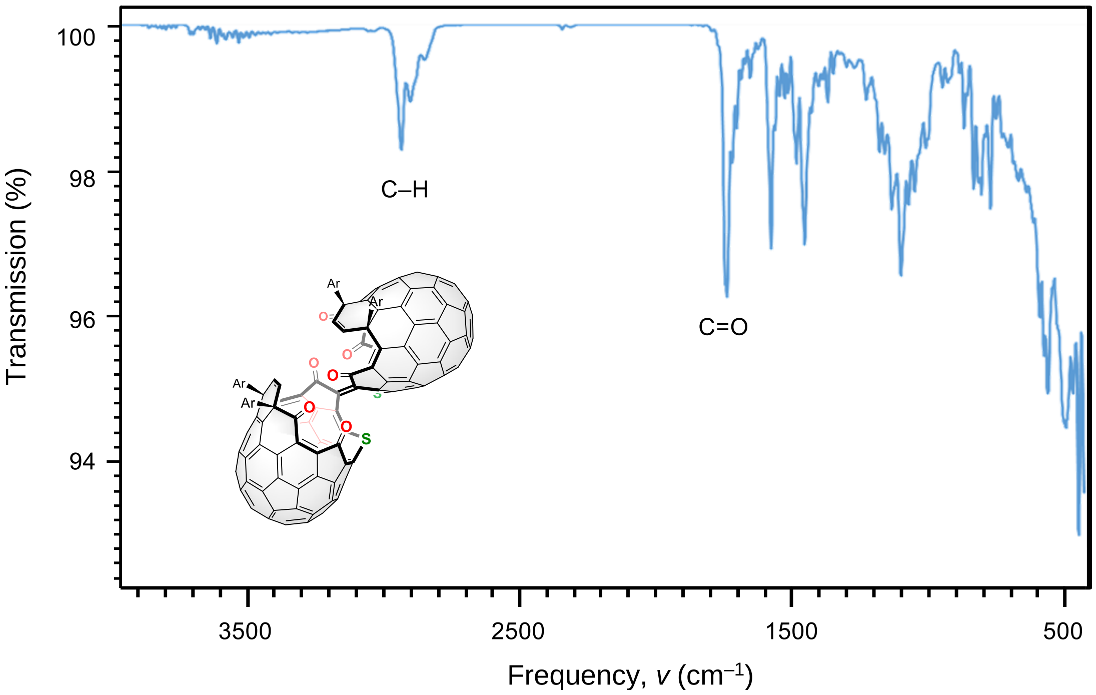

Supplementary Figure 17. IR spectra (KBr) of **4**.

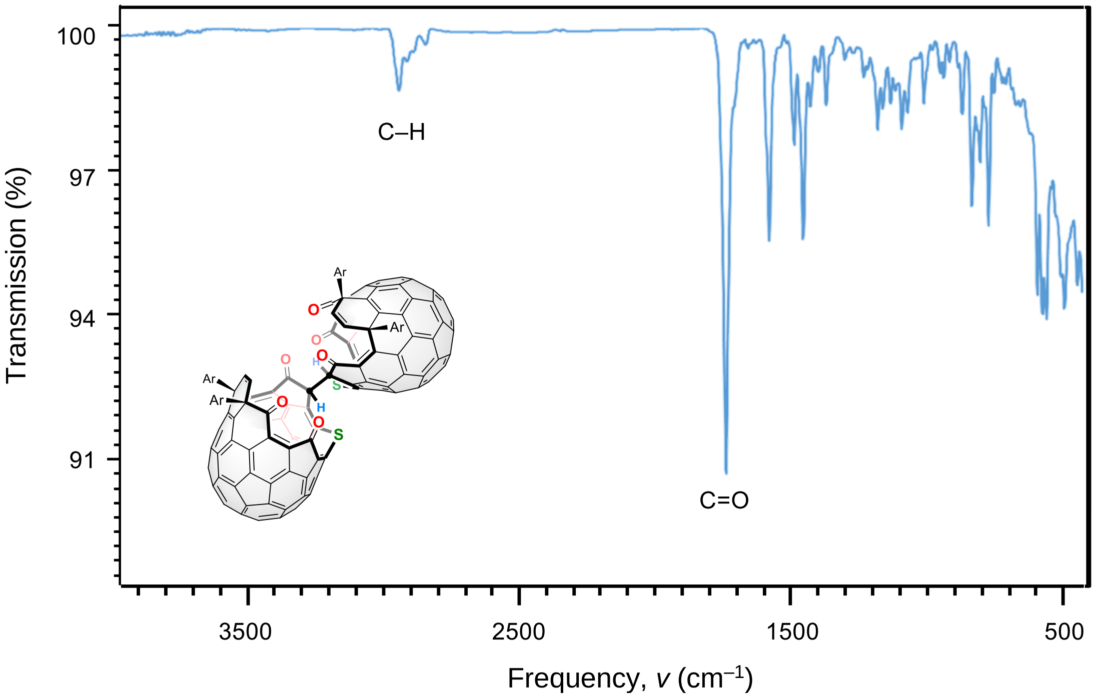

Supplementary Figure 18. IR spectra (KBr) of **5**.

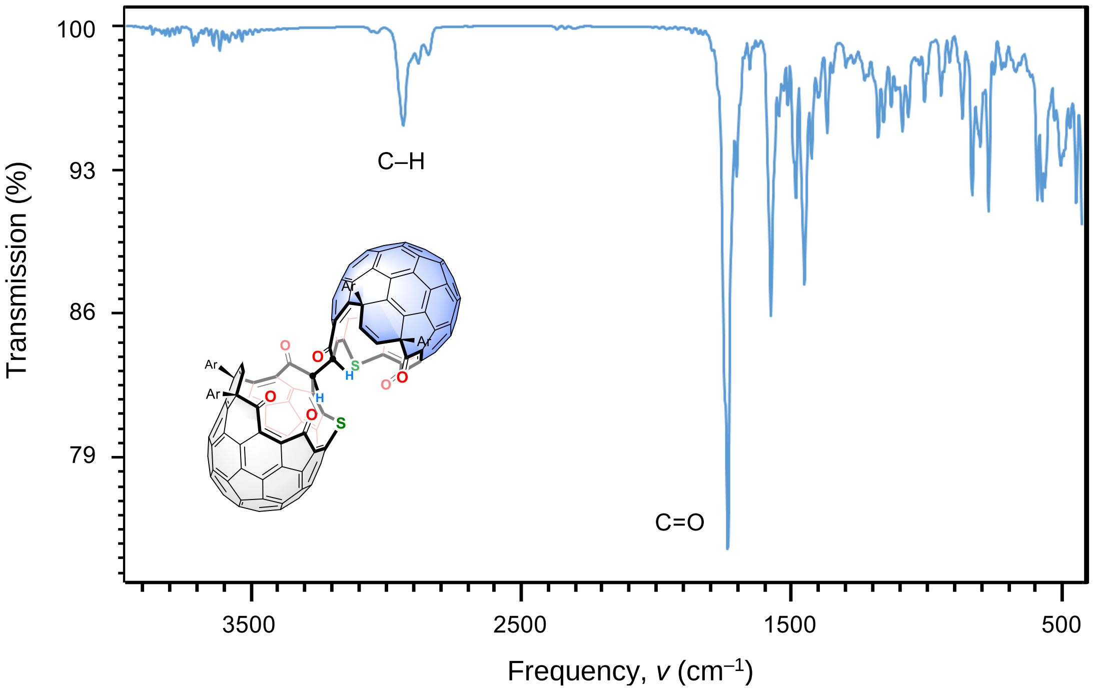

Supplementary Figure 19. IR spectra (KBr) of **6**.

Supplementary Note 5. Association Constant

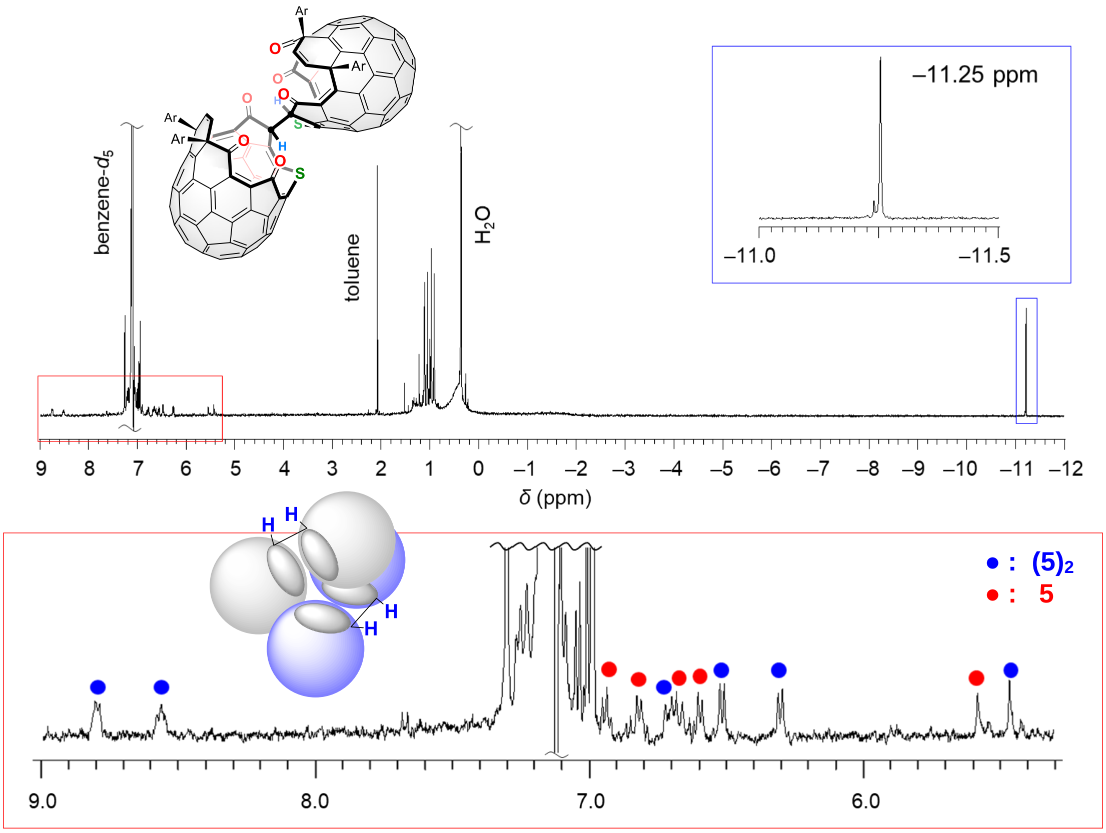

Supplementary Figure 20. ^{1}H NMR spectra (500 MHz, benzene-d_6) of *rac*-**5**.

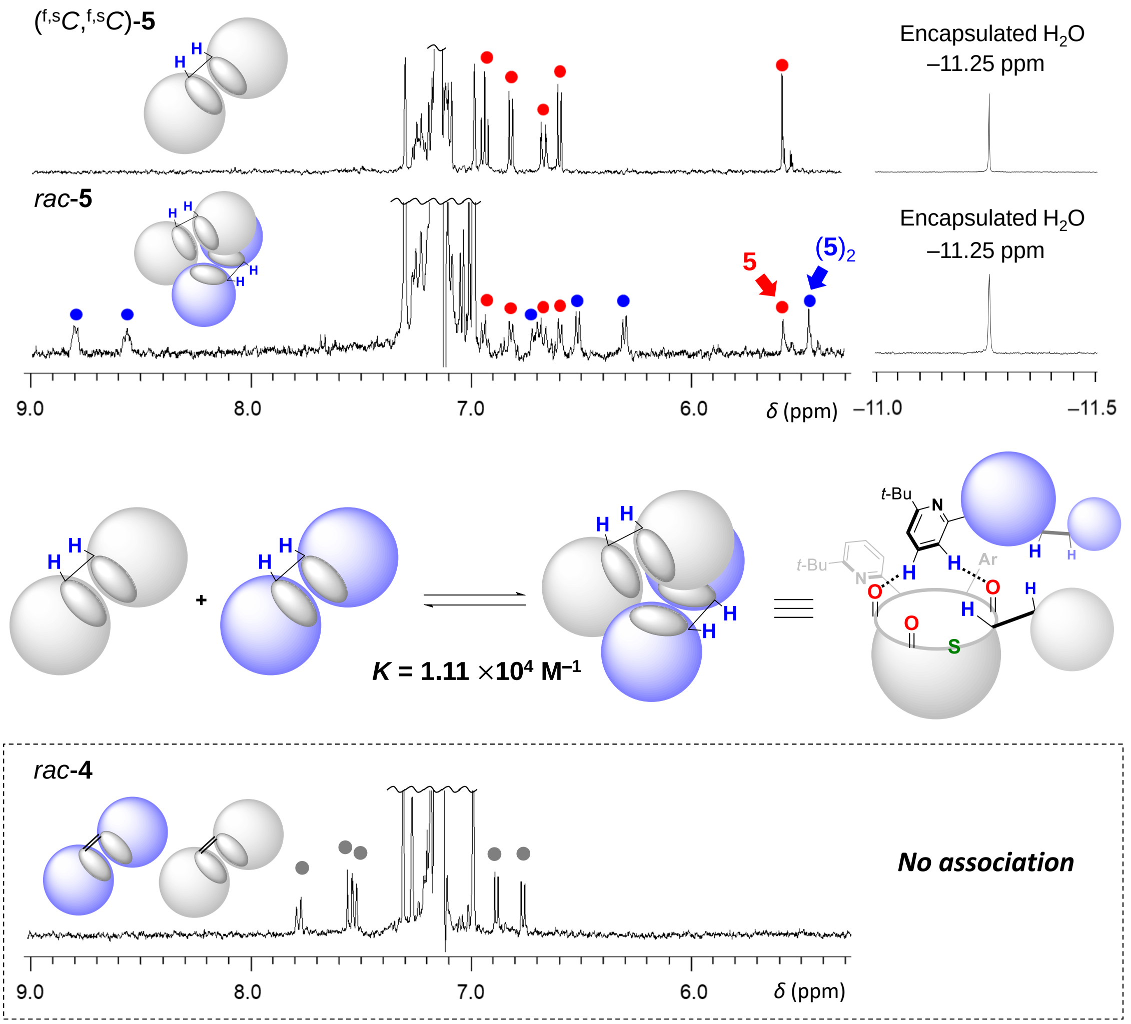

Supplementary Figure 21. ^{1}H NMR spectra (500 MHz, benzene-d_6) of ($^{f,s}C$,$^{f,s}C$)-**5**, *rac*-**5**, and *rac*-**4** with a predicted solution structure of **5**.

Supplementary Note 6. Chirality

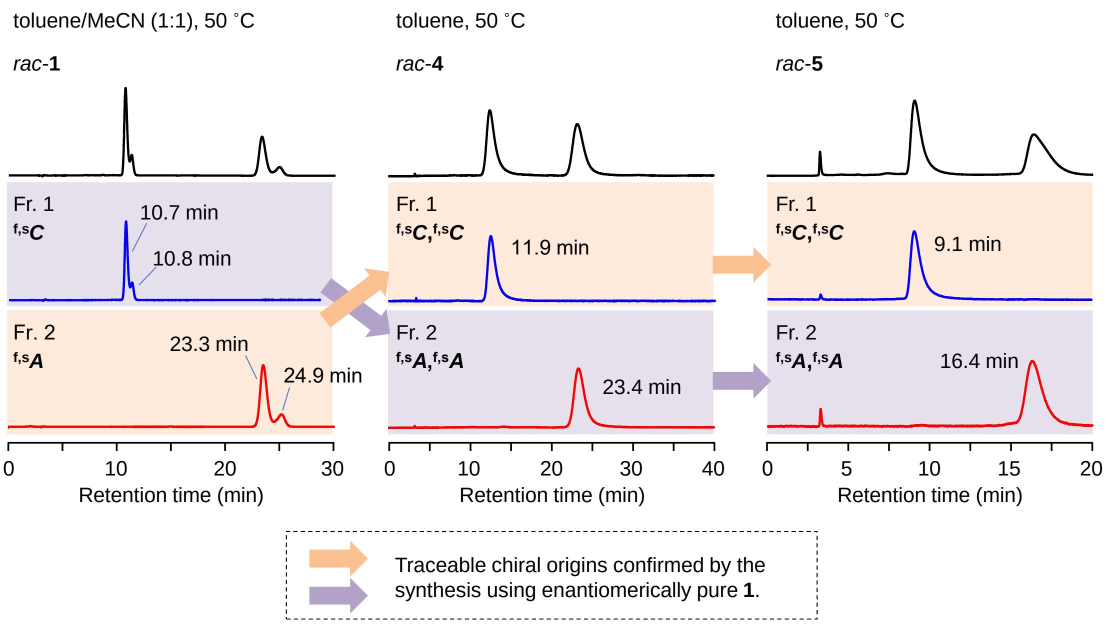

Supplementary Figure 22. Chiral HPLC charts (CHIRALPAK IF, 1 mL min^{-1}, 50 °C, 326 nm) of **1**, **4**, and **5**. In the HPLC charts of **1**, empty and encapsulated ones were separately observed.

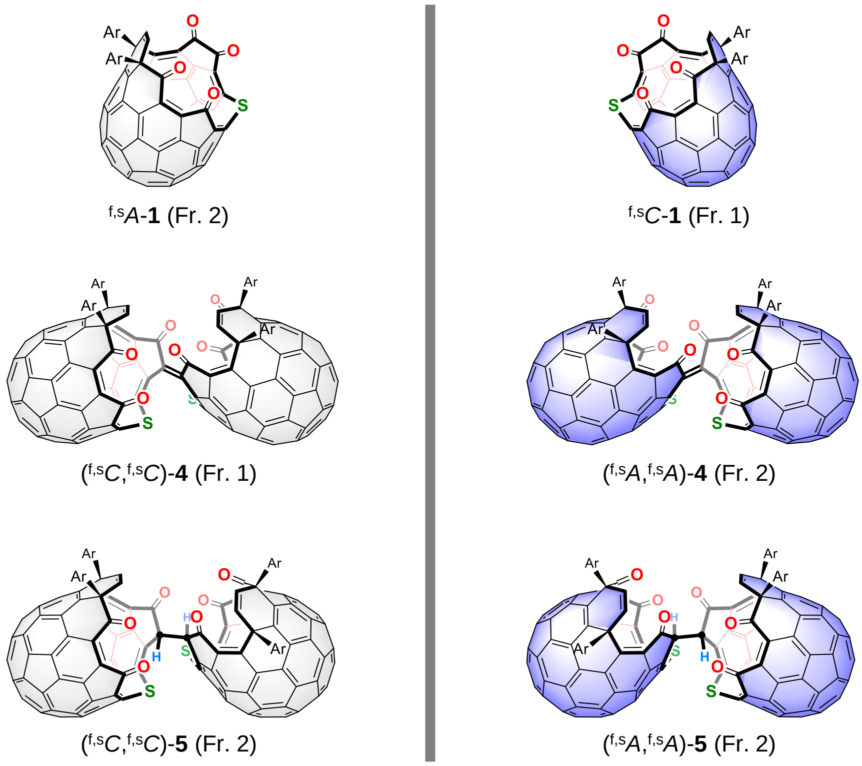

Supplementary Figure 23. Structures of **1**, **4**, and **5** with their chiral descriptors.

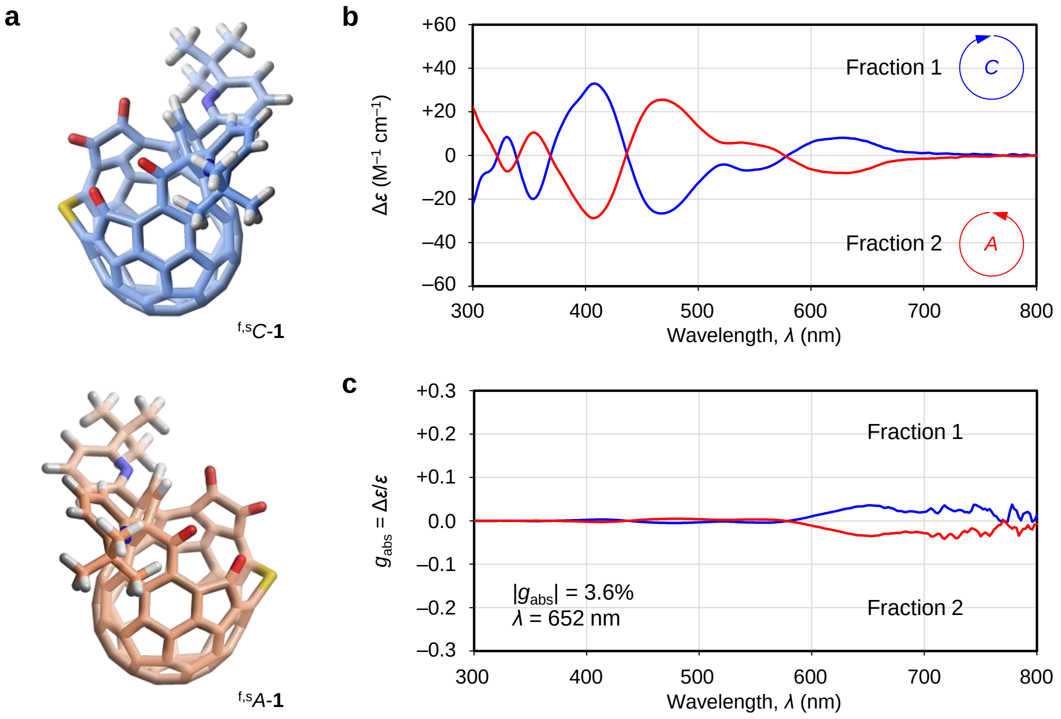

Supplementary Figure 24. Chiroptical properties of 1. a. Structures of two enantiomers. **b.** CD spectra (50 µM in toluene). **c.** Dissymmetry factor g_{abs} spectra.

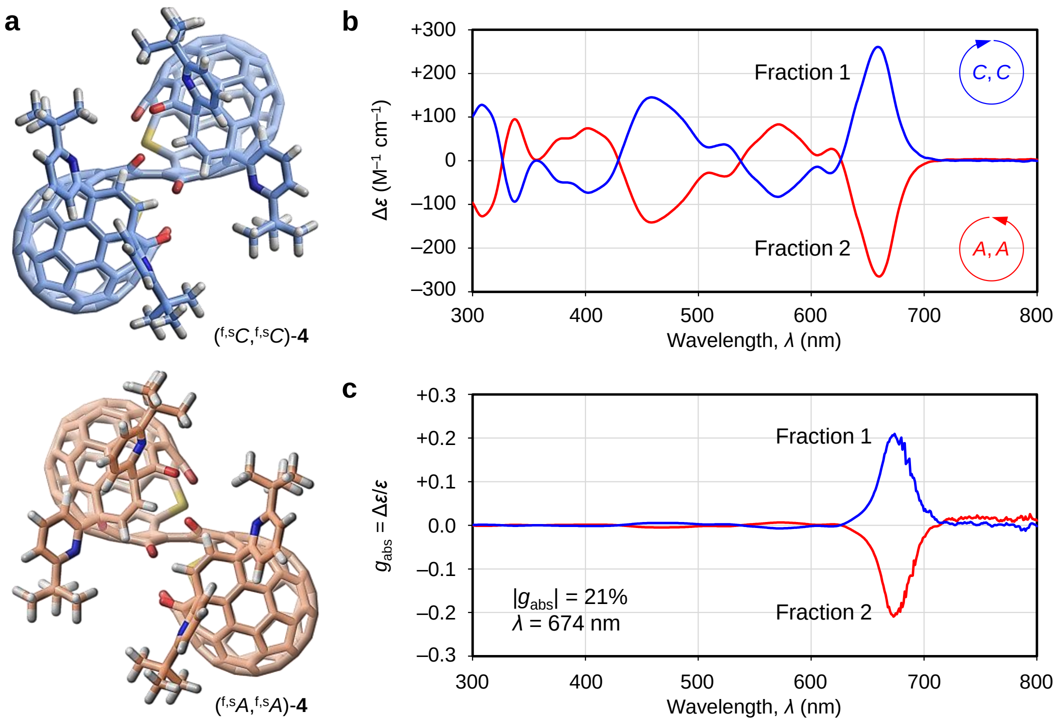

Supplementary Figure 25. Chiroptical properties of 4. a. Structures of two enantiomers. **b.** CD spectra (10 µM in toluene). **c.** Dissymmetry factor g_{abs} spectra.

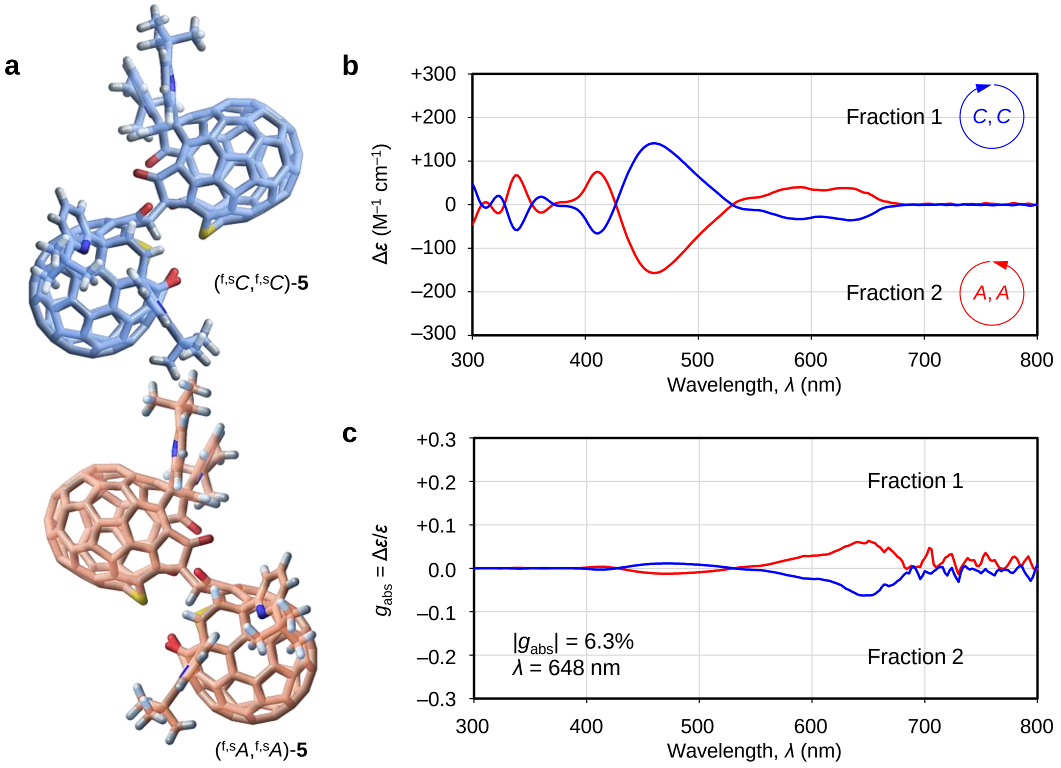

Supplementary Figure 26. Chiroptical properties of 5. a. Structures of two enantiomers. **b.** CD spectra (10 μM in toluene). **c.** Dissymmetry factor g_{abs} spectra.

Supplementary Note 7. Single Crystal X-Ray Structures

[{(Ar)$_{0.34(2)}$(N$_2$)$_{0.47(3)}$}{(Ar)$_{0.28(2)}$(N$_2$)$_{0.59(3)}$}@4]•(CS$_2$)$_{2.64}$•(CHCl$_3$)$_{5.84}$

Single crystals of **4** were obtained from a $CS_2/CHCl_3$ solution. Intensity data were collected at 100 K on a Bruker Single Crystal CCD X-ray Diffractometer (SMART APEX II) with Mo Kα radiation (λ = 0.71073 Å) and graphite monochromator. A total of 61500 reflections were measured at the maximum 2θ angle of 49.94°, of which 22596 were independent reflections (R_{int} = 0.0277). The structure was solved by direct methods (SHELXT-2014/5) and refined by the full-matrix least-squares on F^2 (SHELXL-2018/3)[1]. Within the two fullerene cages, the encapsulated species were disordered, which was solved using appropriate models. Thus, two sets of encapsulated species, i.e., [(Ar1) and (N7–N8)] and [(Ar2) and (N5–N9)] were placed and their occupancies were refined to be [0.34(2) and 0.47(3)] and [0.28(2) and 0.59(3)], respectively. This crystal contains several disordered molecules. Thus, [(C169–Cl15–Cl18–Cl40), (S14–C178–S15), and (S16–C180–S17)], [(C165–Cl2–Cl3–Cl6) and (C165–Cl23–Cl24–Cl25)], [{(S8–C173–S9) and (C168–Cl10–Cl12–Cl41)} and {(S5–C171–S6) and (C172–Cl42–Cl43–Cl44)}], and [{(C176–Cl11–Cl26–Cl27) and (C174–Cl16–Cl17–Cl28)}, {(C170–Cl19–Cl29–Cl38) and (C175–Cl32–Cl33–Cl34)}, and (S11–C177–S13)] were placed and their occupancies were refined to be [0.384(4), 0.163(6), and 0.490(8)], [0.81(2) and 0.19(2)], [0.745(4) and 0.100(3)], and [0.517(3), 0.287(4), and 0.143(6)], respectively. All non-hydrogen atoms were refined anisotropically except for one of the disordered CS_2 molecules. All hydrogen atoms were placed using AFIX instructions. The disordered molecules were refined using DIFIX and SIMU instructions. The crystal data are as follows: $C_{171.48}H_{57.84}Ar_{0.62}Cl_{17.51}N_{6.13}O_6S_{5.28}$; FW = 3114.49, crystal size 0.45 × 0.17 × 0.13 mm^3, triclinic, P-1, a = 14.482(2) Å, b = 20.564(3) Å, c = 23.960(3) Å, α = 68.9060(10)°, β = 78.266(2)°, γ = 80.022(2)°, V = 6479.0(15) Å^3, Z = 2, D_c = 1.596 g cm^{-3}. The refinement converged to R_1 = 0.0736, wR_2 = 0.1928 ($I > 2\sigma(I)$), GOF = 1.036. The data was deposited at the Cambridge Crystallographic Data Centre (CCDC 2211312) and can be obtained free of charge from CCDC at [http://www.ccdc.cam.ac.uk/data_request/cif].

Notes for B-level alerts were shown below:

PLAT234_ALERT_4_B Large Hirshfeld Difference Cl24 --C165 . 0.29 Ang.

PLAT245_ALERT_2_B U(iso) H183 Smaller than U(eq) C175 by 0.052 Ang**2

These alerts are unavoidable due to severely disordered solvent molecules ($CHCl_3$) even at a measured temperature of 100 K.

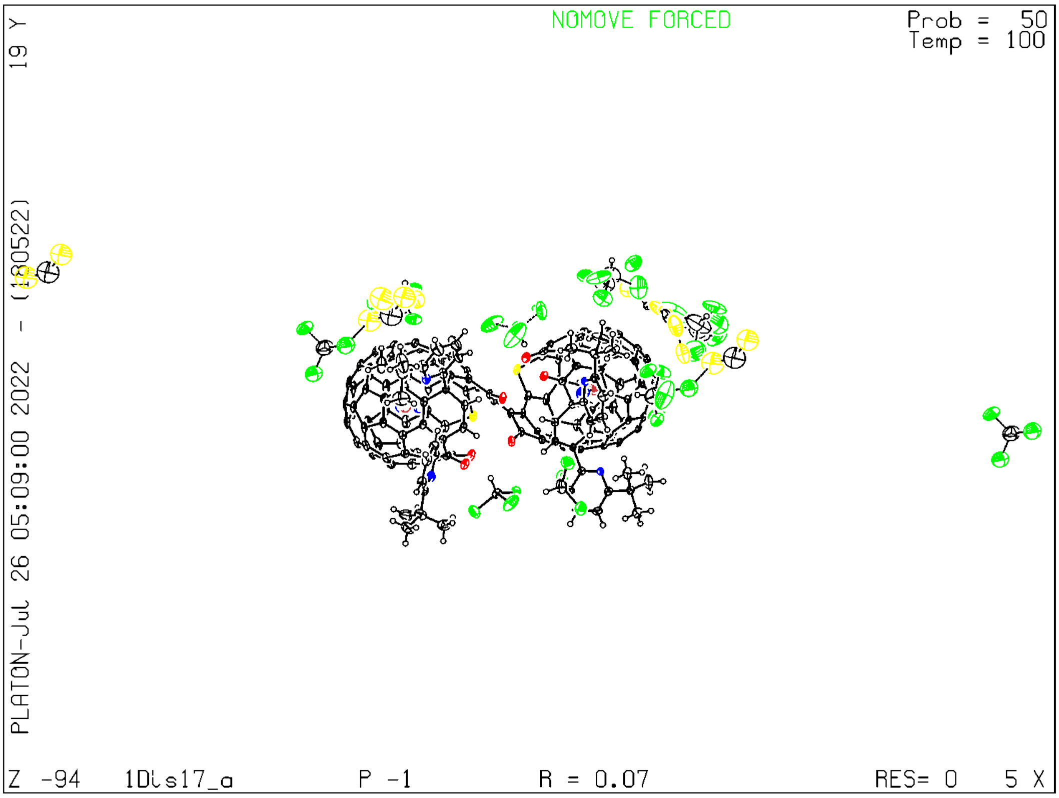

Supplementary Figure 27. Crystal structure of $[\{(Ar)_{0.34(2)}(N_2)_{0.47(3)}\}\{(Ar)_{0.28(2)}(N_2)_{0.59(3)}\}$@**4**]•$(CS_2)_{2.64}$•$(CHCl_3)_{5.84}$. Thermal ellipsoids are shown at 50% probability.

$(H_2O@5)_2•(CS_2)_{3.23}•(CH_3C_6H_5)_{9.02}$

Single crystals of **5** were obtained from a CS_2/toluene solution. Intensity data were collected at 100 K on a Bruker Single Crystal CCD X-ray Diffractometer (SMART APEX II) with Mo Kα radiation (λ = 0.71073 Å) and graphite monochromator. A total of 115079 reflections were measured at the maximum 2θ angle of 50.1°, of which 42951 were independent reflections (R_{int} = 0.0530). The structure was solved by direct methods (SHELXT-2014/5) and refined by the full-matrix least-squares on F^2 (SHELXL-2018/3)[1]. Three of 6-*t*-butylpyridin-2-yl groups were disordered, which were solved using appropriate models. Thus, [(C328–C152–C153–C154) and (C328–C364–C373–C374)], [(N8–C348–C376–C351–C354–C361–C363) and (N9–C231–C232–C233–C234–C235–C236)], and [(C238–C239–C240–C241–C242–C243–C244–C245) and (C362–C377–C381–C241–C379–C378–C380–C382)] were placed and their occupancies were refined to be [0.553(19) and 0.447(19)], [0.538(10) and 0.462(10)], and [0.523(8) and 0.477(8)], respectively. This crystal contains several disordered solvent molecules. Thus, [(C439 to C445) and (C414 to C420)], [(C407 to C413) and (C432 to C438)], [(S5–C346–S6) and (C366 to C372)], [(S11–C384–S14), (S12–C383–S13), and (C385–C387–C388–C389–C390–C391–C392)], [(S7–C350–S8), (S16–C427–S17), and (C386–C421–C422–C423–C424–C425–C426)], [{(S18–C428–S19) and (S9–C375–S10)} and {(C394–C401–C402–C403–C404–C405–C406) and (C332–C333–C334–C335–C337–C343–C349)}], and [(C395–C396–C397–C398–C399–C400–C453), (S15–C430–S22), (C447–C448–C449–C450–C451–C452–C455), and (S23–C431–S24)] were placed and their occupancies were refined to be [0.440(12) and 0.560(12)], [0.504(16) and 0.496(16)], [0.537(10) and 0.463(10)], [0.424(7), 0.424(7), and 0.576(7)], [0.209(6), 0.209(6), and 0.791(6)], [0.179(6) and 0.821(6)], and [0.215(9), 0.201(6), 0.315(12), and 0.269(11)], respectively. The occupancies of two CS_2 molecules, i.e., (S25–C454–S26) and (S20–C429–S21), were refined to be 0.299(6). All non-hydrogen atoms were refined anisotropically except for one of the disordered 6-*t*-butylpyridin-2-yl groups and some of the disordered solvent molecules. All hydrogen atoms were placed using AFIX instructions except for the encapsulated H_2O molecules which were refined using DFIX instructions. Some of the disordered solvent molecules including CS_2 and toluene, one of the encapsulated H_2O molecules, and some of the carbon atoms constituting the fullerene cages were refined using DFIX, SIMU and/or ISOR instructions. The crystal data are as follows: $C_{394.39}H_{188.01}N_8O_{16}S_{10.46}$; FW = 5629.52, crystal size 1.37 × 0.36 × 0.13 mm^3, triclinic, *P*-1, a = 19.364(2) Å, b = 24.487(3) Å, c = 28.535(3) Å, α = 104.4980(10)°, β = 103.212(9)°, γ = 102.8120(10)°, V = 12741(3) Å^3, Z = 2, D_c = 1.467 g cm^{-3}. The refinement converged to R_1 = 0.1252, wR_2 = 0.2710 ($I > 2\sigma(I)$), GOF = 1.176. The data

was deposited at the Cambridge Crystallographic Data Centre (CCDC 2211311) and can be obtained free of charge from CCDC at [http://www.ccdc.cam.ac.uk/data_request/cif].

Notes for B-level alerts were shown below:

_vrf_PLAT420_2sp3PP_a

PROBLEM: D-H Bond Without Acceptor O13 --H13 . Please Check

D-H Bond Without Acceptor O13 --H13A . Please Check

D-H Bond Without Acceptor O14 --H14 . Please Check

D-H Bond Without Acceptor O14 --H14A . Please Check

D-H Bond Without Acceptor O15 --H15 . Please Check

D-H Bond Without Acceptor O15 --H15A . Please Check

D-H Bond Without Acceptor O16 --H16 . Please Check

D-H Bond Without Acceptor O16 --H16A . Please Check

RESPONSE: The encapsulated H2O molecule inside fullerenes behaves like a gaseous single water molecule in vacuo without definitive interactions with H-bonding acceptors as discussed in *ChemPhysChem* **2017**, *18*, 1229–1233. Therefore, the absence of acceptors for the protons are reasonable.

_vrf_PLAT029_2sp3PP_a

PROBLEM: _diffrn_measured_fraction_theta_full value Low . 0.955 Why?

RESPONSE: Due to the fragility of the crystal caused by a continuous release of cocrystalized solvent molecules during measurements, collection at higher resolutions and better completeness may be possible only using a synchrotron which enables short-time measurements.

_vrf_PLAT097_2sp3PP_a

PROBLEM: Large Reported Max. (Positive) Residual Density 1.75 eA-3

RESPONSE: Due to the severe disorders of solvent molecules, large density still remained though they were solved using appropriate models with 11 (for CS2) and 14 (for toluene) dispositions.

_vrf_PLAT220_2sp3PP_a

PROBLEM: NonSolvent Resd 1 C Ueq(max)/Ueq(min) Range 10.0 Ratio

RESPONSE: This is due to the severe disorders of solvent molecules but not by main structures.

_vrf_PLAT221_2sp3PP_a

PROBLEM: Solv./Anion Resd 2 C Ueq(max)/Ueq(min) Range 10.0 Ratio

RESPONSE: This is due to the severe disorders of solvent molecules and inevitable for the crystal of this fullerene compound because many solvent molecules are filled in relatively large voids generated among fullerene cages.

_vrf_PLAT242_2sp3PP_a

PROBLEM: Low 'MainMol' Ueq as Compared to Neighbors of C70 Check

RESPONSE: This is due to the high flexibility of the *t*-butyl group where C70 is a quaternary carbon atom.

_vrf_PLAT332_2sp3PP_a

PROBLEM: Large Phenyl C-C Range C366 -C371 . 0.35 Ang.

RESPONSE: This is due to the severe disorders of toluene molecules and inevitable for the crystal of this fullerene compound.

_vrf_PLAT340_2sp3PP_a

PROBLEM: Low Bond Precision on C-C Bonds .... 0.01073 Ang.

RESPONSE: This is due to the severe disorders of solvent molecules and inevitable for the crystal of this fullerene compound because many solvent molecules are filled in relatively large voids generated among fullerene cages.

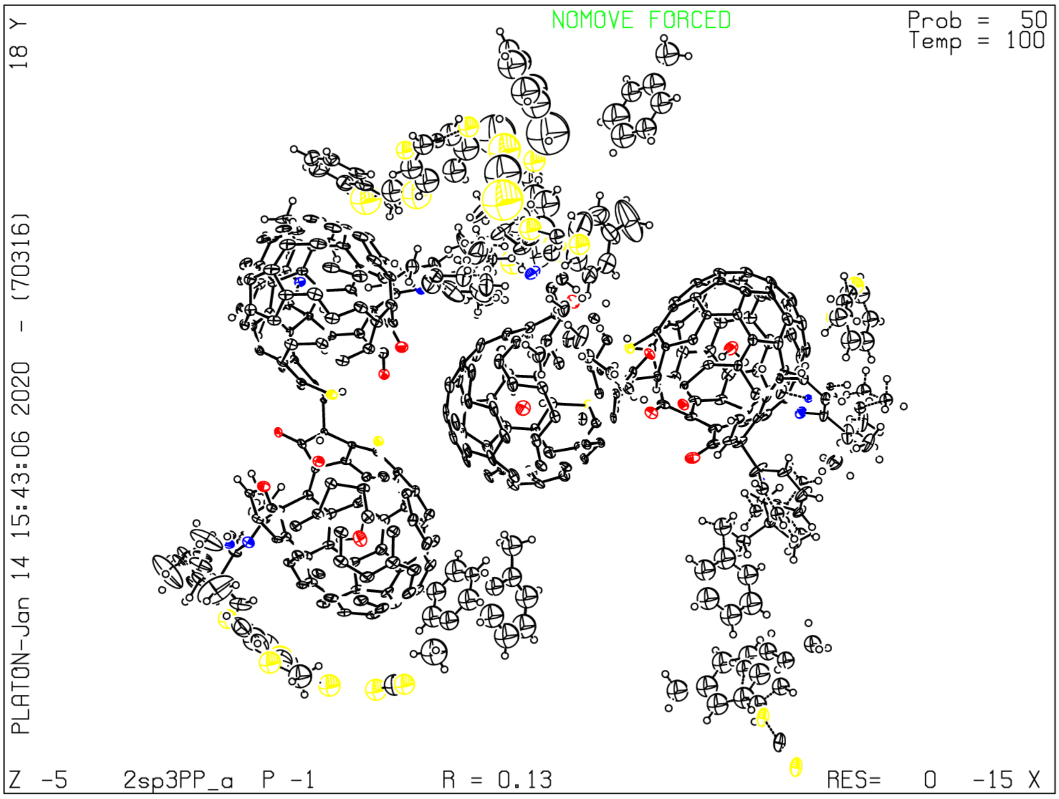

Supplementary Figure 28. Crystal structure of $(H_2O@\mathbf{5})_2$•$(CS_2)_{3.23}$•$(CH_3C_6H_5)_{9.02}$. Thermal ellipsoids are shown at 50% probability.

Supplementary Note 8. DFT Calculations

Plausible Mechanism

All structures were optimized at the M06-2X/6-31G(d,p) level of theory, in which *t*-butyl groups were replaced with hydrogen atoms for the calculations to be simplified. The plausible reaction mechanism was shown in Supplementary Figure 29. In the first step, betaine **INT1** is presumed to be formed by a nucleophilic addition of PMe_3 to **1'**. Then, **INT1** undergoes a C–C bond formation by a reaction with unreacted **1'**, which gives epoxide intermediate **INT2** with a release of $OPMe_3$. The further reaction with the second PMe_3 molecule furnishes conjugated dimer **4'** with releasing $OPMe_3$ again. These dimers are hydrogenated by the assistance of PMe_3 in the presence of water to yield **5'** and **6'**. According to theoretical calculations, the formation of homochiral dimer **4'** is more thermodynamically favorable than that of heterochiral dimer for which two possible conformations were obtained (**4'-hetero** and **4'-hetero-2**), while the hydrogenation is more preferred for the latter. These are matched well with the experimental observation.

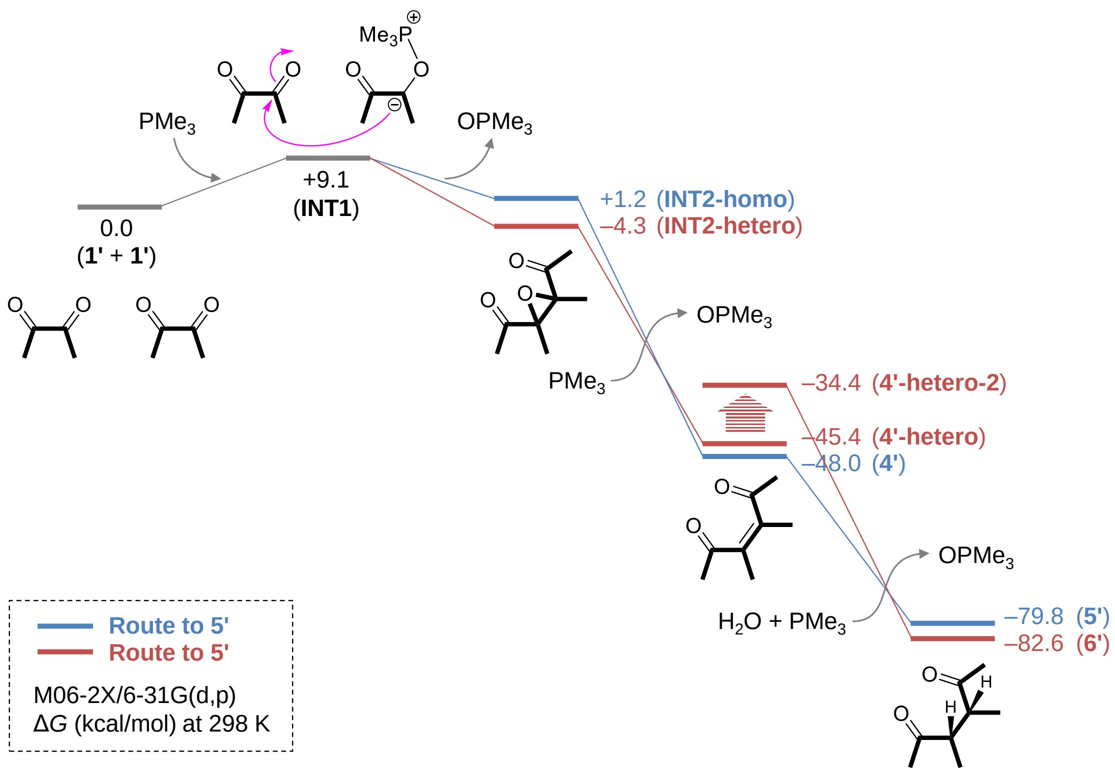

Supplementary Figure 29. Plausible mechanism to produce **4'–6'** (M06-2X/6-31G(d,p), ΔG (kcal mol^{-1}) at 298 K).

Supplementary Table 1. Optimized structure of PMe_3 (M06-2X/6-31G(d,p))

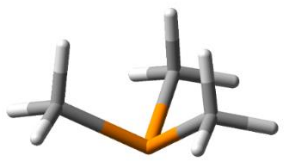

Standard orientation:

Center Number	Atomic Number	Atomic Type	Coordinates (Angstroms) X	Y	Z
1	15	0	0.000455	-0.000429	-0.614924
2	6	0	-0.559194	1.524653	0.283842
3	6	0	1.600411	-0.278319	0.284544
4	6	0	-1.041873	-1.245729	0.284196
5	1	0	-1.587087	1.762671	-0.001497
6	1	0	0.071591	2.370738	-0.000468
7	1	0	-0.513828	1.398839	1.370754
8	1	0	2.019243	-1.246327	-0.002100
9	1	0	2.319346	0.494406	0.000577
10	1	0	1.468888	-0.257224	1.371525
11	1	0	-0.731906	-2.255497	0.003128
12	1	0	-2.089006	-1.124718	-0.004715
13	1	0	-0.960136	-1.140086	1.371164

The total electronic energy was calculated to be –460.9904169 Hartree.

Supplementary Table 2. Optimized structure of $OPMe_3$ (M06-2X/6-31G(d,p))

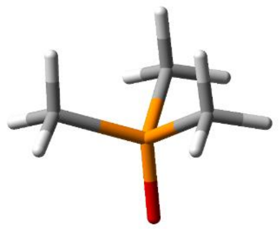

Standard orientation:

Center Number	Atomic Number	Atomic Type	Coordinates (Angstroms) X	Y	Z
1	8	0	0.002208	0.003276	1.684308
2	15	0	0.000360	0.000595	0.190915
3	6	0	1.486482	0.736386	-0.561513
4	6	0	-0.105273	-1.657040	-0.555960
5	6	0	-1.383396	0.917219	-0.558812
6	1	0	1.439158	0.713286	-1.653411
7	1	0	1.581269	1.771175	-0.224889
8	1	0	2.366249	0.182948	-0.225586
9	1	0	-0.104708	-1.608518	-1.648068
10	1	0	-1.022696	-2.142540	-0.215749
11	1	0	0.745329	-2.254191	-0.219919
12	1	0	-2.324710	0.478951	-0.219711
13	1	0	-1.346745	1.955692	-0.222306
14	1	0	-1.343090	0.888676	-1.650851

The total electronic energy was calculated to be –536.2181587 Hartree.

Supplementary Table 3. Optimized structure of H_2O (M06-2X/6-31G(d,p))

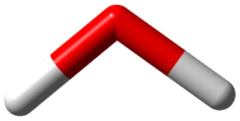

Standard orientation:

Center Number	Atomic Number	Atomic Type	Coordinates (Angstroms) X	Y	Z
1	8	0	-0.000000	0.000000	0.117725
2	1	0	0.000000	-0.760429	-0.470901
3	1	0	0.000000	0.760429	-0.470901

The total electronic energy was calculated to be –76.3839203 Hartree.

Supplementary Table 4. Optimized structure of **1'** (M06-2X/6-31G(d,p))

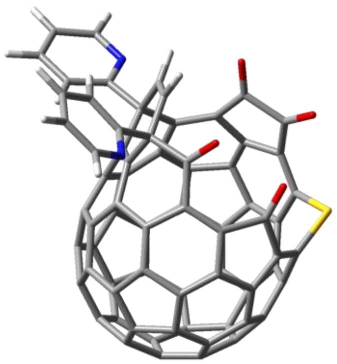

Standard orientation:

Center Number	Atomic Number	Atomic Type	X	Y	Z
1	6	0	3.819525	0.475660	1.389576
2	6	0	3.830525	-0.816832	1.081510
3	6	0	3.009973	-1.443476	-0.014520
4	6	0	1.844986	-2.279323	0.544858
5	6	0	1.502497	-2.345314	1.885992
6	6	0	2.021974	-1.793803	3.200563
7	6	0	0.749538	-1.500959	4.084212
8	6	0	-0.403543	-2.046995	3.319901
9	6	0	-1.732815	-1.675766	3.267334
10	6	0	-2.843306	0.656561	3.004249
11	6	0	-2.249844	1.884162	2.775981
12	6	0	-0.778404	2.244769	3.032317
13	6	0	-0.359955	2.952092	1.718321
14	6	0	0.787757	2.768893	0.958453
15	6	0	2.059787	2.248638	1.562140
16	6	0	3.063513	1.520834	0.618008
17	16	0	-2.259833	-0.360698	4.332598
18	7	0	3.614796	3.847400	0.105621
19	8	0	3.133206	-1.675803	3.635771
20	8	0	2.321652	2.399236	2.727754
21	7	0	4.068727	-3.597151	-0.403078
22	6	0	2.329696	0.942274	-0.566874
23	6	0	1.510745	-0.888860	-1.958895
24	8	0	0.792368	-0.967698	5.157027
25	6	0	1.452311	1.853429	-1.263790
26	6	0	0.909332	-2.787974	-0.462080
27	8	0	-0.104506	1.921347	3.966043
28	6	0	-0.276536	-2.429727	-2.614992
29	6	0	4.926997	-4.395467	-1.040261
30	6	0	-1.433882	-3.147507	-2.118613
31	6	0	3.948602	-2.341406	-0.832220
32	6	0	0.841264	-2.176031	-1.797116
33	6	0	0.996738	0.044595	-2.853094
34	6	0	2.345087	-0.391815	-0.890009
35	6	0	-1.415561	-3.620713	-0.830403
36	6	0	-0.154250	-0.228649	-3.671654
37	6	0	-0.648619	-3.369664	1.321444
38	6	0	-0.784119	-1.447951	-3.552432
39	6	0	0.958623	1.442889	-2.492774
40	6	0	0.697999	2.842723	-0.490759
41	6	0	-0.225873	-3.489620	-0.038755
42	6	0	5.291407	2.211420	-0.409176
43	6	0	4.043416	2.585905	0.091438
44	6	0	-3.657471	-2.724788	-0.414457
45	6	0	-0.454607	3.378070	-1.077782
46	6	0	-0.224306	2.018505	-3.084922
47	6	0	6.127715	3.203402	-0.904817
48	6	0	0.165441	-2.759071	2.224402
49	6	0	-2.237114	-1.500214	-3.563762
50	6	0	5.700882	-3.985228	-2.120988
51	6	0	-4.247901	-1.686044	0.411593
52	6	0	-2.980360	-0.330310	-3.663526
53	6	0	-2.061021	-3.130959	1.357067
54	6	0	5.691981	4.524668	-0.885156
55	6	0	4.685712	-1.828109	-1.901084
56	6	0	-2.590125	-2.128986	2.186966
57	6	0	4.428056	4.791907	-0.371174
58	6	0	-0.922620	0.989911	-3.818029
59	6	0	-4.810227	-0.722359	-0.510891
60	6	0	-4.338717	1.013377	1.113541
61	6	0	-2.554007	-3.399477	0.039711
62	6	0	-1.616275	3.642624	-0.277342
63	6	0	-2.637411	-2.538306	-2.643179
64	6	0	-4.129665	-0.127631	-2.801084
65	6	0	-3.696241	-1.311822	1.654913
66	6	0	5.575346	-2.669962	-2.556291
67	6	0	-1.567333	3.336774	1.067213
68	6	0	-3.776720	0.110550	2.029659
69	6	0	-4.474793	-1.101353	-1.872873
70	6	0	-3.733714	-2.336337	-1.806592
71	6	0	-2.694569	2.683131	1.690940
72	6	0	-2.309386	0.948411	-3.809542
73	6	0	-3.052661	1.944344	-3.062405
74	6	0	-0.924128	2.978883	-2.386085
75	6	0	-4.556366	1.636596	-1.146705
76	6	0	-2.374131	2.949054	-2.376289
77	6	0	-2.804102	3.349470	-1.057087
78	6	0	-3.800083	2.339864	0.947244
79	6	0	-4.855103	0.607713	-0.166724
80	6	0	-3.876571	2.703142	-0.453415
81	6	0	-4.172401	1.275699	-2.431834
82	1	0	4.388591	0.837856	2.240635
83	1	0	4.406909	-1.512548	1.681665
84	1	0	4.997791	-5.413507	-0.664899
85	1	0	5.590279	1.168329	-0.395619
86	1	0	7.107361	2.949346	-1.297369
87	1	0	6.381023	-4.678433	-2.602608
88	1	0	6.314284	5.330935	-1.256117
89	1	0	4.553487	-0.795538	-2.209085
90	1	0	4.047060	5.809446	-0.336410
91	1	0	6.160598	-2.305932	-3.394899

The total electronic energy was calculated to be –3633.172092 Hartree.

Supplementary Table 5. Optimized structure of **INT1** (M06-2X/6-31G(d,p))

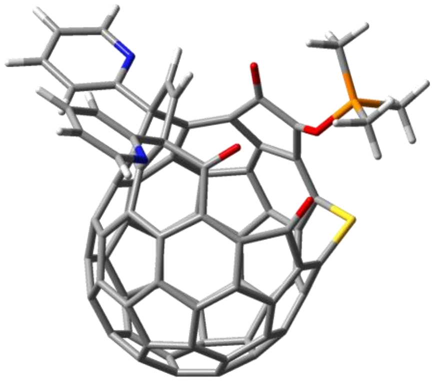

Standard orientation:

Center Number	Atomic Number	Atomic Type	X	Y	Z
1	6	0	3.328742	-1.938014	-1.105421
2	6	0	3.579547	-1.677747	0.172851
3	6	0	2.546180	-1.677021	1.267111
4	6	0	2.153598	-0.254619	1.715119
5	6	0	2.562159	0.908973	1.088959
6	6	0	3.388346	1.263072	-0.160192
7	6	0	2.821760	2.476052	-0.644168
8	6	0	1.607090	2.824748	0.037721
9	6	0	0.423951	3.541224	-0.153830
10	6	0	-0.660896	3.479884	0.805251
11	6	0	-2.076757	3.436983	0.390512
12	6	0	-2.405311	3.180429	-1.027287
13	6	0	-1.347243	3.122267	-2.030015
14	6	0	-1.371148	2.138462	-2.998397
15	6	0	-0.194493	1.342122	-3.562582
16	6	0	-0.673876	-0.127131	-3.402820
17	6	0	-0.051897	-1.236430	-2.842944
18	6	0	1.438603	-1.280172	-2.634029
19	6	0	1.985622	-2.347987	-1.638855
20	6	0	0.979922	-2.549918	-0.531455
21	6	0	1.242043	-2.291698	0.790294
22	6	0	0.176281	-2.353633	1.763368
23	6	0	0.115235	-1.336774	2.808886
24	6	0	1.035667	-0.202633	2.669393
25	6	0	0.538973	1.046708	3.060601
26	6	0	0.841462	2.198166	2.263326
27	6	0	1.747034	2.077033	1.271266
28	6	0	-0.354836	2.987018	2.087275
29	6	0	-1.329646	2.446680	2.970437
30	6	0	-0.781577	1.246524	3.584831
31	6	0	-1.591454	0.167633	3.831513
32	6	0	-1.128624	-1.147513	3.445184
33	6	0	-2.311297	-1.895009	3.065470
34	6	0	-2.235573	-2.835828	2.061056
35	6	0	-0.974772	-3.042430	1.399184
36	6	0	-1.248122	-3.301619	0.004276
37	6	0	-0.356758	-2.867706	-0.965811
38	6	0	-0.870612	-2.255295	-2.192249
39	6	0	-2.262453	-2.227495	-2.352369
40	6	0	-2.895359	-1.096165	-2.967422
41	6	0	-2.100556	-0.052241	-3.390093
42	6	0	-2.512894	1.304957	-3.120828
43	6	0	-3.664139	1.558966	-2.414341
44	6	0	-3.600938	2.514121	-1.337047
45	6	0	-4.533465	2.072442	-0.334309
46	6	0	-4.252193	2.325006	0.986524
47	6	0	-3.034698	3.026244	1.350798
48	6	0	-2.665453	2.521655	2.653469
49	6	0	-3.541216	1.420473	3.009499
50	6	0	-3.020690	0.266190	3.588309
51	6	0	-3.475243	-1.025734	3.139215
52	6	0	-4.489540	-1.128321	2.191371
53	6	0	-4.405135	-2.130293	1.144063
54	6	0	-3.299754	-2.967174	1.088996
55	6	0	-2.676000	-3.255001	-0.181276
56	6	0	-3.179968	-2.729962	-1.351424
57	6	0	-4.345369	-1.867394	-1.305467
58	6	0	-4.160259	-0.840924	-2.303749
59	6	0	-4.533598	0.468535	-2.024026
60	6	0	-5.101317	0.798893	-0.740050
61	6	0	-5.322465	-0.193024	0.206299
62	6	0	-5.043715	0.074744	1.605295
63	6	0	-4.552658	1.318716	1.989278
64	6	0	-4.933319	-1.559693	-0.080754
65	6	0	2.130073	-3.670062	-2.409749
66	6	0	2.754385	-4.759092	-1.795339
67	6	0	2.854553	-5.948490	-2.501255
68	6	0	2.334099	-6.011075	-3.792120
69	6	0	1.735166	-4.871827	-4.314209
70	6	0	3.143618	-2.455253	2.447410
71	6	0	2.917080	-3.823556	2.612943
72	6	0	3.548786	-4.481142	3.661184
73	6	0	4.386264	-3.756590	4.501894
74	6	0	4.555156	-2.399612	4.244930
75	16	0	0.068193	4.156413	-1.783823
76	7	0	1.629875	-3.721154	-3.642835
77	7	0	3.954434	-1.756955	3.243181
78	8	0	0.829229	1.770250	-4.018232
79	8	0	4.422525	0.763742	-0.598138
80	8	0	2.190096	-0.528413	-3.205536
81	8	0	3.614922	3.489962	-1.219651
82	15	0	4.093063	4.733981	-0.387415
83	6	0	5.058109	5.673240	-1.565754
84	6	0	2.658193	5.663131	0.180191
85	6	0	5.090966	4.270246	1.030236
86	1	0	4.120947	-1.835090	-1.840011
87	1	0	4.570536	-1.358062	0.474294
88	1	0	3.151284	-4.653497	-0.790241
89	1	0	3.331267	-6.815161	-2.053942
90	1	0	2.391127	-6.919216	-4.381404
91	1	0	1.318418	-4.875570	-5.318397
92	1	0	2.251094	-4.352928	1.939274
93	1	0	3.387100	-5.542866	3.819554
94	1	0	4.897349	-4.225231	5.335209
95	1	0	5.204875	-1.796090	4.874738
96	1	0	5.812862	5.000022	-1.980786
97	1	0	5.520543	6.539727	-1.089241
98	1	0	4.401204	5.972731	-2.384621
99	1	0	2.116847	6.044370	-0.688365
100	1	0	2.956149	6.486373	0.832925
101	1	0	2.011238	4.971938	0.728088
102	1	0	5.927487	3.662247	0.682332

103	1	0	5.435707	5.171638	1.544491
104	1	0	4.494335	3.641344	1.693645

The total electronic energy was calculated to be –4094.1726238 Hartree..

Supplementary Table 6. Optimized structure of **INT2-homo** (M06-2X/6-31G(d,p))

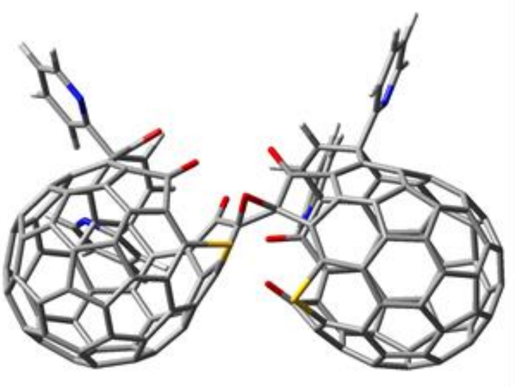

Standard orientation:

Center Number	Atomic Number	Atomic Type	Coordinates (Angstroms) X	Y	Z
1	6	0	3.105446	3.605638	-0.586575
2	16	0	1.883140	-2.764165	-2.509507
3	16	0	-1.075727	-3.019371	0.054103
4	8	0	-0.915060	2.150599	-2.444699
5	8	0	0.233817	2.105420	-0.015495
6	8	0	-1.915670	2.217534	2.138998
7	8	0	2.877550	-0.082226	-4.026589
8	8	0	-1.156984	-0.420795	1.816426
9	8	0	3.411331	2.470346	-3.096911
10	7	0	5.745334	4.611817	-2.681923
11	6	0	7.020841	1.351153	1.807534
12	7	0	4.325651	4.238291	3.105820
13	6	0	9.071773	-1.139610	-1.296068
14	6	0	-6.110594	0.216901	3.340077
15	6	0	-3.922240	-4.106517	-0.734283
16	6	0	-4.168298	-1.210547	3.524430
17	6	0	2.661172	3.317021	0.631441
18	6	0	3.952562	-0.503401	-3.719784
19	6	0	3.646592	-2.872940	-2.657640
20	6	0	9.137001	-1.433815	1.147134
21	6	0	-5.273832	0.457051	-2.954860
22	6	0	9.262056	-1.969623	-0.194240
23	6	0	-2.950369	3.492640	-0.117994
24	6	0	-8.594086	-0.482052	-1.449057
25	6	0	-3.097934	2.095089	1.941139
26	6	0	-3.953362	-2.783569	-2.801059
27	6	0	8.579486	0.749573	0.178287
28	6	0	-5.128855	3.593413	-2.938155
29	6	0	-5.504696	-0.999973	3.800770
30	6	0	-3.856550	3.067889	1.002105
31	6	0	1.903601	-2.425473	-0.763877
32	6	0	8.221981	-3.591026	1.152959
33	6	0	-7.474961	-0.098614	2.979762
34	6	0	2.732123	-2.954461	1.465742
35	6	0	-5.036920	-0.826939	-3.461173
36	6	0	-8.045290	0.540125	1.899182
37	6	0	-3.732821	-2.507111	3.060990
38	6	0	3.377013	3.309633	2.964498
39	6	0	5.364306	-1.923779	3.584382
40	6	0	-5.022706	6.438328	3.332256
41	6	0	4.359035	2.365828	-2.361553
42	6	0	4.292354	5.002694	4.198362
43	6	0	6.074667	6.681628	-0.842990
44	6	0	-9.095392	-1.546174	1.147899
45	6	0	-6.037718	-3.323604	3.213561
46	6	0	-2.598994	-2.362866	2.218728
47	6	0	3.318853	4.890807	5.186547
48	6	0	-8.391963	-3.650940	0.396476
49	6	0	-4.812646	5.408755	1.178390
50	6	0	6.682508	-4.416104	-1.072493
51	6	0	-3.901748	1.020783	2.613076
52	6	0	8.717373	0.252894	-1.100540
53	6	0	-5.336086	-3.093318	-3.014758
54	6	0	-3.212381	-3.243714	-1.704813
55	6	0	6.418475	5.721517	-3.003040
56	6	0	-7.696488	-1.519015	3.173483
57	6	0	-7.997987	-2.606566	-2.214024
58	6	0	-7.284759	-1.614483	-2.989797
59	6	0	6.606424	6.782920	-2.127136
60	6	0	-6.830182	-4.047667	2.249323
61	6	0	2.347694	3.128010	3.889069
62	6	0	-6.463242	-2.082006	3.671207
63	6	0	-9.059289	-2.427034	-0.005236
64	6	0	-6.011583	-1.879171	-3.427653
65	6	0	6.423282	-2.839241	3.218175
66	6	0	-5.962696	-3.985446	-2.185155
67	6	0	-5.197031	6.495033	1.951256
68	6	0	-8.036808	-3.528359	1.799166
69	6	0	7.946647	-3.733564	-1.283078
70	6	0	-4.921433	3.482404	-4.312180
71	6	0	-4.480427	5.282752	3.880048
72	6	0	2.322392	3.936086	5.019330
73	6	0	-5.346254	1.168558	2.652537
74	6	0	-7.348147	-3.775450	-1.822203
75	6	0	-8.479728	-2.230920	2.268136
76	6	0	-6.391415	5.481623	-3.204460
77	6	0	-5.495893	4.432495	-5.150066
78	6	0	-6.253332	5.453165	-4.589724
79	6	0	7.255909	-0.659190	3.147019
80	6	0	-5.450135	2.300329	-0.820111
81	6	0	7.835138	0.287012	2.329642
82	6	0	-5.135832	2.439543	0.506704
83	6	0	8.803841	-0.098339	1.324412
84	7	0	-4.106509	4.227697	3.149538
85	6	0	5.671430	1.477370	2.120411
86	7	0	-5.842096	4.581353	-2.390413
87	6	0	-1.478780	1.133589	-2.166225
88	6	0	5.268340	0.258251	-3.406889
89	6	0	3.893253	-4.027172	-0.417054
90	6	0	-7.612730	1.378394	-0.234016
91	6	0	4.790905	-4.143086	1.920925

92	6	0	5.847480	-0.560613	3.471132
93	6	0	1.266862	-1.302831	-0.268326
94	6	0	7.484488	1.641776	0.471006
95	6	0	-3.763212	-1.439120	-3.251769
96	6	0	6.693588	1.511280	-1.853234
97	6	0	4.336881	-1.964669	-3.436255
98	6	0	3.779033	-3.333965	2.367195
99	6	0	-1.684070	-1.293478	-1.989991
100	6	0	4.376840	-3.780425	-1.785645
101	6	0	-2.868415	0.708908	-2.565727
102	6	0	5.752875	-2.039254	-3.505870
103	6	0	-6.238194	-4.766663	-0.026540
104	6	0	3.612731	0.200632	2.698062
105	6	0	4.475583	3.244941	-1.085534
106	6	0	8.511765	-2.439649	1.987024
107	6	0	2.826965	1.100186	1.849428
108	6	0	-7.587670	-0.293288	-2.472636
109	6	0	-4.468451	2.638329	-1.932744
110	6	0	7.758904	0.639017	-2.112436
111	6	0	5.034240	0.471294	2.963800
112	6	0	6.581072	2.051155	-0.496604
113	6	0	6.139098	-3.935604	2.405443
114	6	0	-2.480419	-3.175267	1.106875
115	6	0	2.268433	-1.671794	1.900323
116	6	0	-8.607582	0.345119	-0.346797
117	6	0	7.042323	-4.299815	1.342656
118	6	0	-1.991023	-2.501860	-1.381486
119	6	0	-2.725622	-0.699640	-2.777007
120	6	0	7.565530	-0.499022	-2.965035
121	6	0	-3.335463	-0.193390	2.977149
122	6	0	7.613853	-2.058509	2.976487
123	6	0	5.301852	2.540480	-0.040246
124	6	0	-6.644181	1.586952	-1.210817
125	6	0	5.738366	-3.973320	-2.063669
126	6	0	-4.260899	4.302409	1.829731
127	6	0	6.233800	-4.685614	0.198460
128	6	0	4.069720	-2.199195	3.222556
129	6	0	5.471457	1.394082	-2.634122
130	6	0	6.448411	-3.089074	-2.953404
131	6	0	-4.129370	1.280348	-2.572374
132	6	0	1.639591	-0.844976	1.026978
133	6	0	0.836626	1.071139	0.028224
134	6	0	-8.879469	-0.186901	0.972223
135	6	0	0.552141	-0.155024	-0.912272
136	6	0	-6.600249	0.704730	-2.372414
137	6	0	1.789536	0.576018	1.095268
138	6	0	5.229300	4.534023	-1.458115
139	6	0	2.748755	-3.250192	0.094629
140	6	0	7.787717	-2.886765	-2.440360
141	6	0	-4.568098	-4.285682	1.654525
142	6	0	-3.581476	-4.021182	0.694751
143	6	0	4.854812	2.261546	1.224376
144	6	0	-4.642238	-3.523080	2.875607
145	6	0	-5.237431	-4.515116	-1.045450
146	6	0	3.417093	2.508797	1.655737
147	6	0	-8.840150	-1.904263	-1.274054
148	6	0	-7.268981	1.507582	1.162077
149	6	0	5.373335	5.536961	-0.494807
150	6	0	-5.911177	-4.656070	1.304322
151	6	0	3.192881	-1.128409	2.843262
152	6	0	8.341147	-1.611878	-2.448819
153	6	0	-2.135574	-0.905817	2.316256
154	6	0	4.831215	-4.515594	0.519241
155	6	0	-5.977824	1.858010	1.525376
156	6	0	-7.530022	-4.288206	-0.487310
157	6	0	6.312539	-0.707338	-3.504227
158	6	0	-3.244327	3.342411	-1.404063
159	6	0	8.687665	-3.300456	-0.191130
160	6	0	-0.714353	-0.166899	-1.715377
161	8	0	0.573166	-0.076268	-2.307838
162	1	0	2.474191	4.145652	-1.285330
163	1	0	1.666064	3.627657	0.929356
164	1	0	-2.015681	3.956378	0.181404
165	1	0	-5.307454	7.266197	3.971653
166	1	0	5.083775	5.743501	4.286198
167	1	0	6.209367	7.482518	-0.122844
168	1	0	3.342108	5.538265	6.055683
169	1	0	-4.948008	5.390628	0.099755
170	1	0	6.825351	5.755388	-4.010652
171	1	0	7.160911	7.660155	-2.440900
172	1	0	1.581544	2.378426	3.717558
173	1	0	-5.630466	7.374133	1.484789
174	1	0	-4.320807	2.673452	-4.713732
175	1	0	-4.339276	5.191163	4.954209
176	1	0	1.535180	3.819610	5.757251
177	1	0	-6.969204	6.268189	-2.724531
178	1	0	-5.351494	4.373416	-6.224131
179	1	0	-6.722894	6.213366	-5.203440
180	1	0	4.954796	5.396134	0.498694
181	1	0	-2.551022	3.697975	-2.157555

The total electronic energy was calculated to be –7191.1435704 Hartree.

Supplementary Table 7. Optimized structure of **INT2-hetero** (M06-2X/6-31G(d,p))

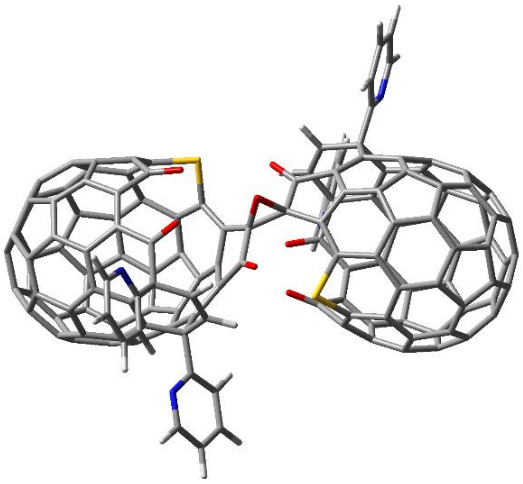

Standard orientation:

Center Number	Atomic Number	Atomic Type	X	Y	Z
1	6	0	-2.957278	3.650400	-0.021337
2	16	0	-1.238291	-2.981561	-0.070540
3	8	0	-0.971070	2.266554	-1.988456
4	8	0	-1.121771	-0.534153	1.922460
5	8	0	-1.863233	2.090298	1.985718
6	7	0	-3.894939	4.173538	3.325170
7	6	0	-7.646965	1.625985	-0.134665
8	7	0	-5.840128	4.814774	-2.246137
9	6	0	-7.771583	-1.379098	3.177967
10	6	0	-3.268941	3.542349	-1.307072
11	6	0	-2.172124	-0.955372	2.318815
12	6	0	-2.637780	-3.136628	0.992710
13	6	0	-9.199767	-1.295082	1.174033
14	6	0	-8.591440	-2.035299	2.262542
15	6	0	-8.072189	0.729607	1.976568
16	6	0	-2.158835	-2.408644	-1.484347
17	6	0	-8.575196	-3.395249	0.345077
18	6	0	-4.150343	-2.568755	-2.880358
19	6	0	-5.155979	3.822469	-2.822862
20	6	0	-7.443848	-1.285832	-2.986346
21	6	0	-3.066598	2.080469	1.921357
22	6	0	-6.388628	5.737012	-3.035312
23	6	0	-5.066582	6.512047	2.359955
24	6	0	-6.277973	5.727465	-4.423578
25	6	0	-6.117618	-4.511666	1.185576
26	6	0	-7.504102	0.038251	3.025044
27	6	0	-4.195542	5.185087	4.145701
28	6	0	-4.774437	6.372192	3.715241
29	6	0	-4.977278	3.728957	-4.201908
30	6	0	-8.180731	-2.279540	-2.235356
31	6	0	-7.004774	-3.905208	2.162107
32	6	0	-5.550720	4.702082	-5.014021
33	6	0	-8.698289	-0.162614	-1.394923
34	6	0	-8.673816	0.627871	-0.265963
35	6	0	-8.943612	0.061480	1.039298
36	6	0	-6.686764	1.836355	-1.118880
37	6	0	-3.364255	-0.200700	2.959722
38	6	0	-4.139192	-3.958510	-0.857672
39	6	0	-6.190641	-3.723975	-2.278222
40	6	0	-7.697189	0.027169	-2.424311
41	6	0	-1.823167	-1.203574	-2.074805
42	6	0	-7.276653	1.695062	1.259617
43	6	0	-5.334189	1.237665	2.697296
44	6	0	-2.716379	-2.374140	2.142137
45	6	0	-5.544787	-2.825020	-3.085349
46	6	0	-3.774542	-3.931325	0.567254
47	6	0	-3.847725	-2.501978	2.988293
48	6	0	-5.360335	0.718449	-2.905571
49	6	0	-3.826974	3.152829	1.096659
50	6	0	-9.207370	-2.138129	-0.007535
51	6	0	-4.181759	1.490210	-2.504451
52	6	0	-6.122813	0.295638	3.370763
53	6	0	-6.677021	0.990176	-2.305649
54	6	0	-5.968181	1.986984	1.610722
55	6	0	-7.563788	-3.480505	-1.890098
56	6	0	-3.922920	-1.215994	-3.288826
57	6	0	-7.746354	-4.031135	-0.570568
58	6	0	-5.554957	-0.954978	3.786830
59	6	0	-8.987486	-1.581611	-1.261838
60	6	0	-5.130837	2.584928	0.599735
61	6	0	-4.757859	-4.198776	1.530557
62	6	0	-6.465800	-4.567451	-0.143185
63	6	0	-6.185549	-1.576455	-3.449051
64	6	0	-3.893381	1.039593	2.623984
65	6	0	-4.794128	-3.477895	2.778910
66	6	0	-2.854918	-0.530167	-2.805269
67	6	0	-1.537756	1.208925	-2.049970
68	6	0	-0.786899	-0.135434	-1.867376
69	6	0	-2.934179	0.876119	-2.527410
70	6	0	-4.165120	4.326320	2.031369
71	6	0	-3.413516	-3.090376	-1.808255
72	6	0	-6.177005	-3.240634	3.139234
73	6	0	-5.468607	2.500998	-0.724261
74	6	0	-4.493479	2.844555	-1.839746
75	6	0	-4.760578	5.471243	1.495150
76	6	0	-5.176518	-0.556087	-3.455994
77	6	0	-6.552294	-1.999295	3.639840
78	6	0	-5.470328	-4.314893	-1.165937
79	6	0	-4.230094	-1.202517	3.490482
80	6	0	-8.198363	-3.331012	1.745784
81	16	0	1.778273	2.830505	-2.195913
82	8	0	1.084953	-2.492813	-1.521262
83	8	0	4.170673	-1.987877	-3.761477
84	8	0	3.243261	0.604454	-3.954241
85	6	0	8.062568	0.140524	-1.878185
86	6	0	3.405900	3.807936	0.355911
87	6	0	6.535736	1.577492	-3.080353
88	6	0	3.493001	-1.046322	2.352455
89	6	0	3.825487	-3.694187	-1.599574
90	6	0	6.916837	0.108347	3.435795
91	6	0	5.012484	-1.921472	-2.903287
92	6	0	2.255528	2.185270	1.799970
93	6	0	3.711896	-4.125036	1.928375
94	6	0	7.763803	1.412786	-2.471538
95	6	0	5.158900	-3.049701	-1.844843
96	6	0	8.863896	0.399603	-0.701674
97	6	0	2.873749	0.147353	2.747149
98	6	0	8.695659	-0.394457	0.413036
99	6	0	5.785280	2.790209	-2.851321

100	6	0	7.908356	-5.920854	-3.370829
101	6	0	8.832186	1.555042	1.895156
102	6	0	7.584496	3.621031	-1.414438
103	6	0	4.394546	2.523991	-2.951781
104	6	0	7.613825	3.521000	2.269479
105	6	0	6.345017	-5.278685	-1.672397
106	6	0	5.967792	-0.764853	-2.810774
107	6	0	3.164988	2.454862	2.872723
108	6	0	2.331421	2.818677	0.550937
109	6	0	9.017963	1.832864	-0.544444
110	6	0	5.772503	2.093454	3.891384
111	6	0	4.830077	0.995146	3.910937
112	6	0	7.524315	4.191407	-0.090866
113	6	0	8.320603	2.463855	-1.640151
114	6	0	7.996152	2.250686	2.857097
115	6	0	3.546405	1.190262	3.469924
116	6	0	4.083711	3.463987	2.742491
117	6	0	7.248547	-6.202721	-2.176412
118	6	0	8.227670	3.611176	0.957048
119	6	0	2.712006	-4.191673	2.897314
120	6	0	7.638058	-4.714114	-4.003468
121	6	0	7.131117	-0.902976	-1.946775
122	6	0	5.403499	3.308390	3.317073
123	6	0	8.990007	2.400533	0.727372
124	6	0	4.704459	-6.032262	2.711776
125	6	0	2.741743	-5.241156	3.810839
126	6	0	3.760464	-6.181034	3.724804
127	6	0	5.159530	-2.550424	0.641971
128	6	0	5.766530	-2.495161	-0.583916
129	7	0	6.770912	-3.813348	-3.530034
130	7	0	4.685001	-5.035866	1.829153
131	6	0	1.217110	-1.504518	-0.849696
132	6	0	7.105399	-1.547053	1.672070
133	6	0	1.956604	0.785696	1.862269
134	6	0	1.166190	0.874982	-0.411296
135	6	0	1.900016	-1.230666	0.477041
136	6	0	5.576038	4.563325	1.359559
137	6	0	5.513792	-0.232801	3.552713
138	6	0	3.734566	-3.051672	0.828010
139	6	0	3.516236	3.149407	-2.085721
140	6	0	7.702371	-0.541488	2.508667
141	6	0	1.658712	2.152260	-0.556599
142	6	0	1.531079	0.131369	0.750716
143	6	0	5.644738	0.489655	-3.311892
144	6	0	5.764110	-1.900618	1.778389
145	6	0	6.137025	-4.101244	-2.396099
146	6	0	2.930975	-1.798784	1.225166
147	6	0	8.688011	0.184941	1.735242
148	6	0	4.908707	-1.203011	2.731264
149	6	0	5.376135	4.334578	-1.043728
150	6	0	4.036477	3.926204	-0.969276
151	6	0	6.275289	3.767801	-2.016760
152	6	0	4.188519	4.164112	1.475783
153	6	0	7.080259	1.541313	3.625301
154	6	0	7.728961	-1.463600	0.371990
155	6	0	6.162485	4.649006	0.119114
156	6	0	4.243777	1.115849	-3.543371
157	6	0	6.991738	-1.755512	-0.764321
158	6	0	6.332695	4.018325	2.474089
159	6	0	3.217099	-3.714792	-0.420518
160	6	0	0.599099	-0.164361	-1.317283
161	8	0	0.380709	-0.101116	-2.690537
162	1	0	-2.028911	4.127670	0.276837
163	1	0	-2.596366	3.942386	-2.056892
164	1	0	-6.942490	6.526338	-2.532549
165	1	0	-5.527552	7.420317	1.984683
166	1	0	-6.745847	6.505336	-5.016044
167	1	0	-3.960447	5.030796	5.195821
168	1	0	-4.993695	7.162004	4.424816
169	1	0	-4.402368	2.914443	-4.629122
170	1	0	-5.429131	4.656361	-6.091551
171	1	0	-4.981346	5.517725	0.431191
172	1	0	3.352808	-4.147545	-2.464833
173	1	0	8.620956	-6.616414	-3.799579
174	1	0	5.816227	-5.436026	-0.735077
175	1	0	7.440168	-7.129866	-1.645290
176	1	0	1.932988	-3.437467	2.934934
177	1	0	8.138055	-4.450190	-4.932131
178	1	0	5.513944	-6.750462	2.603464
179	1	0	1.978732	-5.320307	4.578794
180	1	0	3.825102	-7.011980	4.417837
181	1	0	2.251543	-4.200036	-0.323959

The total electronic energy was calculated to be –7191.1520818 Hartree.

Supplementary Table 8. Optimized structure of **4'** (M06-2X/6-31G(d,p))

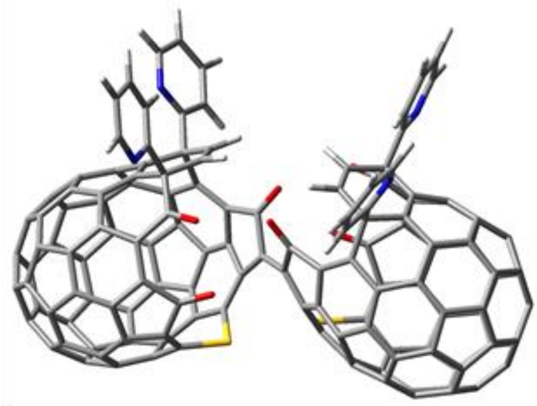

Standard orientation:

Center Number	Atomic Number	Atomic Type	Coordinates (Angstroms) X	Y	Z
1	6	0	2.723693	3.604188	-0.245761
2	16	0	2.862189	-2.541023	-3.622257
3	16	0	-1.176267	-3.322023	-0.478950
4	8	0	-0.787319	2.005469	-2.287928
5	8	0	2.089885	1.470523	-1.786604
6	8	0	-1.356567	1.463128	1.506984
7	8	0	3.864789	0.609522	-3.999153
8	8	0	-0.576455	-1.086802	1.964419
9	8	0	4.080313	3.228102	-2.725164
10	7	0	5.851210	5.142188	-0.987417
11	6	0	5.910729	1.035720	2.823999

12	7	0	2.437187	3.507683	3.509896
13	6	0	9.092202	-0.658032	0.176863
14	6	0	-5.485654	0.036741	3.598351
15	6	0	-4.191187	-4.107532	-0.982172
16	6	0	-3.684573	-1.569699	3.481976
17	6	0	1.943168	2.959657	0.611259
18	6	0	4.877890	0.178588	-3.516011
19	6	0	4.565095	-2.405944	-3.141958
20	6	0	8.430997	-1.444350	2.413742
21	6	0	-5.379540	0.718908	-2.691774
22	6	0	9.027101	-1.686652	1.113669
23	6	0	-2.529145	3.394591	0.008666
24	6	0	-8.577436	-0.071448	-0.868271
25	6	0	-2.522554	1.633984	1.769837
26	6	0	-4.349174	-2.636287	-2.933869
27	6	0	7.959726	0.863032	1.723410
28	6	0	-5.009166	3.800155	-2.519466
29	6	0	-4.956502	-1.260422	3.912821
30	6	0	-3.282346	2.848934	1.188852
31	6	0	2.234068	-2.575842	-1.947733
32	6	0	7.821160	-3.604719	1.741743
33	6	0	-6.909584	-0.131461	3.398040
34	6	0	2.458493	-3.405663	0.334951
35	6	0	-5.323015	-0.538631	-3.302306
36	6	0	-7.546895	0.633337	2.444815
37	6	0	-3.428426	-2.860771	2.883581
38	6	0	1.850367	2.428439	2.981599
39	6	0	4.171889	-2.625976	3.335357
40	6	0	-3.408188	5.860282	4.187909
41	6	0	4.636015	2.874002	-1.715869
42	6	0	1.921379	4.009355	4.631499
43	6	0	5.164534	6.795799	1.150359
44	6	0	-8.866888	-1.291613	1.691936
45	6	0	-5.767686	-3.477079	3.240529
46	6	0	-2.375758	-2.754305	1.935770
47	6	0	0.795857	3.485502	5.262352
48	6	0	-8.451647	-3.380316	0.717424
49	6	0	-4.049924	5.113900	2.004403
50	6	0	7.152701	-4.049777	-0.970152
51	6	0	-3.307945	0.674079	2.622741
52	6	0	8.544172	0.648137	0.493195
53	6	0	-5.765053	-2.806608	-2.991035
54	6	0	-3.520960	-3.239107	-1.972485
55	6	0	6.460146	6.317378	-0.797962
56	6	0	-7.241054	-1.538541	3.525586
57	6	0	-8.270521	-2.171407	-1.843607
58	6	0	-7.563142	-1.179002	-2.630522
59	6	0	6.151080	7.181116	0.245201
60	6	0	-6.726115	-4.050719	2.329300
61	6	0	0.727250	1.820426	3.540163
62	6	0	-6.020176	-2.243747	3.831888
63	6	0	-9.048412	-2.080325	0.486484
64	6	0	-6.376680	-1.509212	-3.233165
65	6	0	5.392520	-3.390802	3.194083
66	6	0	-6.374480	-3.715263	-2.164176
67	6	0	-4.075346	6.092857	2.986049
68	6	0	-7.924404	-3.398167	2.069888
69	6	0	8.325506	-3.260260	-0.636046
70	6	0	-4.997824	3.743538	-3.912766
71	6	0	-2.746195	4.650467	4.350787
72	6	0	0.184519	2.374495	4.694863
73	6	0	-4.717501	0.958716	2.870446
74	6	0	-7.683270	-3.414354	-1.615831
75	6	0	-8.187461	-2.109626	2.678178
76	6	0	-6.160906	5.777057	-2.538438
77	6	0	-5.611016	4.767154	-4.627323
78	6	0	-6.211145	5.807710	-3.929706
79	6	0	5.939889	-1.196375	3.776820
80	6	0	-5.163653	2.411286	-0.451153
81	6	0	6.634767	-0.072948	3.385530
82	6	0	-4.678303	2.409771	0.829270
83	6	0	7.907869	-0.193084	2.706506
84	7	0	-2.715863	3.695567	3.415891
85	6	0	4.527405	1.020006	2.673412
86	7	0	-5.572997	4.805323	-1.842525
87	6	0	-1.420574	0.937698	-2.212332
88	6	0	5.957704	0.939357	-2.723044
89	6	0	4.261893	-4.010395	-1.211778
90	6	0	-7.304373	1.606297	0.338971
91	6	0	4.413112	-4.518616	1.238042
92	6	0	4.500399	-1.241454	3.619789
93	6	0	1.342722	-1.617558	-1.514871
94	6	0	6.739786	1.628903	1.800518
95	6	0	-4.095843	-1.266318	-3.272636
96	6	0	6.731602	1.952420	-0.633310
97	6	0	5.325309	-1.285850	-3.441369
98	6	0	3.222971	-3.872715	1.452865
99	6	0	-1.878299	-1.366676	-2.239784
100	6	0	5.111952	-3.434362	-2.269233
101	6	0	-2.916913	0.741177	-2.536707
102	6	0	6.698395	-1.262967	-3.072370
103	6	0	-6.466046	-4.627204	-0.044657
104	6	0	2.567350	-0.470596	2.264254
105	6	0	4.217779	3.427790	-0.321285
106	6	0	7.698127	-2.632404	2.810697
107	6	0	2.056413	0.521237	1.322883
108	6	0	-7.684639	0.117117	-1.993856
109	6	0	-4.294599	2.754270	-1.651688
110	6	0	7.904681	1.187215	-0.686663
111	6	0	3.777173	-0.175006	3.051476
112	6	0	6.151785	2.206959	0.686113
113	6	0	5.510160	-4.321510	2.163260
114	6	0	-2.450839	-3.451723	0.744626
115	6	0	1.761175	-2.234669	0.785247
116	6	0	-8.391684	0.665655	0.281211
117	6	0	6.735726	-4.406516	1.409888
118	6	0	-2.212216	-2.630894	-1.748626
119	6	0	-2.971527	-0.651258	-2.848136
120	6	0	8.094402	0.229610	-1.737536
121	6	0	-2.820660	-0.596906	2.899802
122	6	0	6.495609	-2.510603	3.497099
123	6	0	4.749038	2.531747	0.770268
124	6	0	-6.449428	1.821192	-0.736426
125	6	0	6.504163	-3.466931	-2.113189
126	6	0	-3.353979	3.931476	2.271208
127	6	0	6.372290	-4.602854	0.016570
128	6	0	3.104342	-2.897922	2.519755
129	6	0	5.814287	1.934485	-1.761801
130	6	0	7.326366	-2.364878	-2.543931
131	6	0	-4.119741	1.414327	-2.384580
132	6	0	1.350574	-1.306951	-0.115337
133	6	0	1.392391	0.646534	-1.266668
134	6	0	-8.550933	0.054793	1.583666
135	6	0	0.637106	-0.422190	-2.136316
136	6	0	-6.609024	1.027288	-1.950745
137	6	0	1.451863	0.093942	0.147867
138	6	0	4.896511	4.795915	-0.127381
139	6	0	2.915310	-3.454922	-0.988857
140	6	0	8.420549	-2.200094	-1.608332
141	6	0	-4.573266	-4.423840	1.448163

142	6	0	-3.678439	-4.156794	0.400142
143	6	0	3.957179	1.940503	1.716923
144	6	0	-4.445635	-3.769129	2.725917
145	6	0	-5.570848	-4.379096	-1.158245
146	6	0	2.442390	1.971857	1.635518
147	6	0	-8.927895	-1.479733	-0.762196
148	6	0	-6.780204	1.588505	1.684612
149	6	0	4.522610	5.579935	0.968274
150	6	0	-5.977370	-4.651255	1.238778
151	6	0	2.283363	-1.824117	2.040339
152	6	0	8.801317	-0.923095	-1.211896
153	6	0	-1.702511	-1.411324	2.211550
154	6	0	4.928466	-4.602867	-0.114542
155	6	0	-5.425492	1.795170	1.898414
156	6	0	-7.756092	-4.012749	-0.307765
157	6	0	7.079809	0.065469	-2.657150
158	6	0	-2.982912	3.363641	-1.236836
159	6	0	8.640323	-3.018842	0.695542
160	6	0	-0.847756	-0.360028	-2.015704
161	8	0	1.071582	-0.267016	-3.514190
162	15	0	0.831798	0.822271	-4.637681
163	6	0	-0.897597	0.945138	-5.167385
164	6	0	1.675399	0.066718	-6.040414
165	6	0	1.449183	2.482177	-4.360601
166	1	0	2.297128	4.271410	-0.988797
167	1	0	0.862440	3.082722	0.579928
168	1	0	-1.552777	3.818138	0.220205
169	1	0	-3.399697	6.599457	4.981045
170	1	0	2.429316	4.878728	5.043718
171	1	0	4.903384	7.432957	1.989487
172	1	0	0.416196	3.941581	6.170400
173	1	0	-4.545687	5.237811	1.044377
174	1	0	7.233664	6.572911	-1.518280
175	1	0	6.676034	8.124076	0.348857
176	1	0	0.270731	0.957490	3.066499
177	1	0	-4.603654	7.026277	2.819100
178	1	0	-4.523432	2.910645	-4.420392
179	1	0	-2.208855	4.430771	5.270714
180	1	0	-0.710855	1.943054	5.132360
181	1	0	-6.615938	6.574088	-1.954664
182	1	0	-5.621886	4.748194	-5.712604
183	1	0	-6.706242	6.624074	-4.443045
184	1	0	3.764214	5.216537	1.657700
185	1	0	-2.365568	3.745991	-2.041590
186	1	0	-1.417528	1.705573	-4.585476
187	1	0	-1.396916	-0.019796	-5.046242
188	1	0	-0.895019	1.220935	-6.225887
189	1	0	1.622046	0.737923	-6.901169
190	1	0	2.712599	-0.119232	-5.766063
191	1	0	1.179256	-0.876804	-6.278387
192	1	0	1.229351	3.045835	-5.274138
193	1	0	2.520158	2.457403	-4.156297
194	1	0	0.914878	2.903516	-3.508343

The total electronic energy was calculated to be –7115.9942043 Hartree.

Supplementary Table 9. Optimized structure of **4'-hetero** (M06-2X/6-31G(d,p))

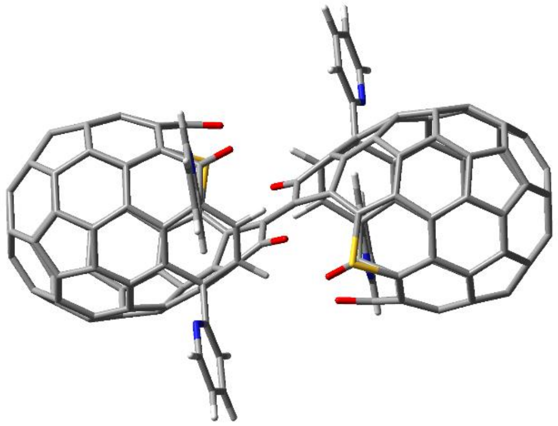

Standard orientation:

Center Number	Atomic Number	Atomic Type	Coordinates (Angstroms) X	Y	Z
1	6	0	-3.476063	3.725920	0.829064
2	6	0	-3.299168	3.645665	-0.483668
3	6	0	-4.221344	2.931655	-1.436410
4	6	0	-3.633986	1.617915	-1.990111
5	6	0	-2.422964	1.043574	-1.610320
6	6	0	-1.294112	1.358794	-0.637810
7	6	0	-0.602787	0.035135	-0.305290
8	6	0	-1.451006	-1.017075	-0.890304
9	6	0	-1.926620	-2.255584	-0.482478
10	6	0	-2.961434	-2.952603	-1.236172
11	6	0	-3.934400	-3.873873	-0.618622
12	6	0	-4.085587	-3.883673	0.844657
13	6	0	-3.206073	-3.063548	1.661191
14	6	0	-3.718164	-2.357478	2.734085
15	6	0	-3.341429	-0.929294	3.145302
16	6	0	-4.721261	-0.252583	3.352094
17	6	0	-5.161624	0.983309	2.896032
18	6	0	-4.200008	2.092852	2.577600
19	6	0	-4.664503	3.161639	1.551275
20	6	0	-5.676122	2.564700	0.608762
21	6	0	-5.515128	2.516998	-0.750558
22	6	0	-6.485344	1.832581	-1.568655
23	6	0	-6.022290	1.038954	-2.700902
24	6	0	-4.567848	0.834306	-2.809620
25	6	0	-4.151648	-0.404708	-3.313020
26	6	0	-3.003274	-1.034964	-2.750339
27	6	0	-2.201487	-0.340154	-1.905897
28	6	0	-3.300503	-2.410224	-2.485230
29	6	0	-4.525926	-2.698383	-3.166388
30	6	0	-5.055267	-1.454082	-3.690044
31	6	0	-6.407223	-1.223010	-3.682986
32	6	0	-6.895928	0.054395	-3.201939
33	6	0	-8.184655	-0.210738	-2.595413
34	6	0	-8.592033	0.529592	-1.507136
35	6	0	-7.720881	1.551544	-0.993689
36	6	0	-7.872430	1.575639	0.442234
37	6	0	-6.788285	1.899244	1.241839

38	6	0	-6.543149	1.125934	2.461617
39	6	0	-7.469238	0.125862	2.783701
40	6	0	-7.021065	-1.122495	3.332691
41	6	0	-5.665010	-1.309077	3.508805
42	6	0	-5.065579	-2.566964	3.126802
43	6	0	-5.831444	-3.565829	2.572480
44	6	0	-5.328435	-4.228512	1.395646
45	6	0	-6.468118	-4.575768	0.590477
46	6	0	-6.332755	-4.589277	-0.777376
47	6	0	-5.057538	-4.262607	-1.381694
48	6	0	-5.371653	-3.650542	-2.658876
49	6	0	-6.803737	-3.467034	-2.765380
50	6	0	-7.314622	-2.271318	-3.267455
51	6	0	-8.439616	-1.642273	-2.617183
52	6	0	-9.058612	-2.260293	-1.536839
53	6	0	-9.502561	-1.470393	-0.402721
54	6	0	-9.277642	-0.101473	-0.398589
55	6	0	-8.823447	0.553697	0.804543
56	6	0	-8.626289	-0.164101	1.964967
57	6	0	-8.861200	-1.595208	1.971216
58	6	0	-7.851855	-2.193431	2.812651
59	6	0	-7.263570	-3.395269	2.436103
60	6	0	-7.667602	-4.044850	1.212932
61	6	0	-8.669375	-3.495053	0.423026
62	6	0	-8.532810	-3.510664	-1.022605
63	6	0	-7.408819	-4.078685	-1.609212
64	6	0	-9.277889	-2.237550	0.807429
65	6	0	-5.371302	4.289508	2.322473
66	6	0	-5.759528	5.442678	1.634678
67	6	0	-6.413407	6.437187	2.347444
68	6	0	-6.656934	6.246332	3.706071
69	6	0	-6.241519	5.055795	4.288831
70	6	0	-4.531311	3.931526	-2.562864
71	6	0	-3.899581	3.878307	-3.804292
72	6	0	-4.184934	4.867806	-4.739472
73	6	0	-5.084857	5.870952	-4.404155
74	6	0	-5.653953	5.841358	-3.133496
75	16	0	-1.531746	-2.807352	1.157733
76	8	0	-3.114166	2.164129	3.093981
77	8	0	-0.931680	2.420459	-0.195887
78	8	0	-2.245421	-0.447442	3.165456
79	7	0	-5.386229	4.902454	-2.227648
80	7	0	-5.611363	4.087999	3.615663
81	6	0	3.476055	-3.726040	-0.828764
82	6	0	3.299122	-3.645624	0.483953
83	6	0	4.221283	-2.931518	1.436639
84	6	0	3.633945	-1.617696	1.990171
85	6	0	2.422946	-1.043364	1.610299
86	6	0	1.294143	-1.358620	0.637733
87	6	0	0.602813	-0.034980	0.305163
88	6	0	1.451026	1.017252	0.890140
89	6	0	4.151625	0.405015	3.312935
90	6	0	1.926668	2.255719	0.482223
91	6	0	2.961490	2.952771	1.235867
92	6	0	3.934484	3.873964	0.618248
93	6	0	4.085687	3.883625	-0.845031
94	6	0	3.206159	3.063447	-1.661499
95	6	0	3.718249	2.357249	-2.734308
96	6	0	3.341488	0.929029	-3.145381
97	6	0	4.721308	0.252267	-3.352101
98	6	0	5.161648	-0.983595	-2.895931
99	6	0	4.200009	-2.093089	-2.577400
100	6	0	4.664501	-3.161818	-1.551009
101	6	0	5.676113	-2.564810	-0.608532
102	6	0	5.515097	-2.516979	0.750781
103	6	0	6.485310	-1.832493	1.568825
104	6	0	6.022246	-1.038744	2.700982
105	6	0	4.567806	-0.834048	2.809646
106	6	0	2.201483	0.340387	1.905786
107	6	0	6.895896	-0.054158	3.201945
108	6	0	6.407211	1.223300	3.682870
109	6	0	5.055260	1.454402	3.689887
110	6	0	4.525954	2.698671	3.166120
111	6	0	3.300531	2.410483	2.484971
112	6	0	3.003270	1.035248	2.750187
113	6	0	5.371708	3.650768	2.658538
114	6	0	5.057624	4.262732	1.381298
115	6	0	6.332856	4.589315	0.776965
116	6	0	6.468234	4.575678	-0.590886
117	6	0	5.328552	4.228378	-1.396037
118	6	0	5.831556	3.565565	-2.572800
119	6	0	5.065673	2.566663	-3.127029
120	6	0	5.665080	1.308726	-3.508906
121	6	0	7.021132	1.122133	-3.332769
122	6	0	7.469277	-0.126182	-2.783665
123	6	0	6.543169	-1.126209	-2.461499
124	6	0	6.788289	-1.899421	-1.241656
125	6	0	7.872429	-1.575759	-0.442068
126	6	0	7.720860	-1.551530	0.993852
127	6	0	8.592021	-0.529544	1.507215
128	6	0	8.184639	0.210895	2.595416
129	6	0	8.439629	1.642426	2.617059
130	6	0	7.314639	2.271552	3.267261
131	6	0	6.803786	3.467235	2.765075
132	6	0	7.408896	4.078771	1.608861
133	6	0	8.532881	3.510675	1.022316
134	6	0	8.669464	3.494928	-0.423312
135	6	0	7.667711	4.044674	-1.213279
136	6	0	7.263677	3.394986	-2.436395
137	6	0	7.851940	2.193101	-2.812824
138	6	0	8.861267	1.594935	-1.971326
139	6	0	8.626328	0.163834	-1.964949
140	6	0	8.823463	-0.553863	-0.804458
141	6	0	9.277655	0.101409	0.398619
142	6	0	9.502598	1.470327	0.402628
143	6	0	9.058652	2.260339	1.536668
144	6	0	9.277956	2.237377	-0.807593
145	6	0	5.371301	-4.289733	-2.322134
146	6	0	5.759499	-5.442874	-1.634275
147	6	0	6.413377	-6.437429	-2.346977
148	6	0	6.656932	-6.246648	-3.705609
149	6	0	6.241542	-5.056135	-4.288437
150	6	0	4.531133	-3.931241	2.563255
151	6	0	3.899026	-3.877980	3.804494
152	6	0	4.184220	-4.867350	4.739853
153	6	0	5.084371	-5.870415	4.404896
154	6	0	5.653843	-5.840870	3.134408
155	16	0	1.531815	2.807367	-1.158034
156	8	0	0.931770	-2.420287	0.195768
157	8	0	3.114169	-2.164398	-3.093780
158	8	0	2.245470	0.447200	-3.165499
159	7	0	5.611386	-4.088296	-3.615330
160	7	0	5.386272	-4.902086	2.228382
161	1	0	-2.734205	4.217519	1.450221
162	1	0	-2.417110	4.087507	-0.932828
163	1	0	-5.558633	5.528333	0.569292
164	1	0	-6.732018	7.348898	1.851700
165	1	0	-7.162777	6.999105	4.300060
166	1	0	-6.420493	4.862025	5.343498
167	1	0	-3.200846	3.081087	-4.032719

168	1	0	-3.709087	4.851353	-5.714904
169	1	0	-5.340236	6.660795	-5.101302
170	1	0	-6.358304	6.611563	-2.827389
171	1	0	2.734206	-4.217701	-1.449882
172	1	0	2.417038	-4.087396	0.933137
173	1	0	5.558582	-5.528477	-0.568890
174	1	0	6.731965	-7.349119	-1.851178
175	1	0	7.162777	-6.999457	-4.299549
176	1	0	6.420538	-4.862421	-5.343111
177	1	0	3.200115	-3.080830	4.032631
178	1	0	3.708078	-4.850865	5.715139
179	1	0	5.339637	-6.660161	5.102194
180	1	0	6.358383	-6.611017	2.828588

The total electronic energy was calculated to be –7115.9870402 Hartree.

Supplementary Table 10. Optimized structure of **4'-hetero-2** (M06-2X/6-31G(d,p))

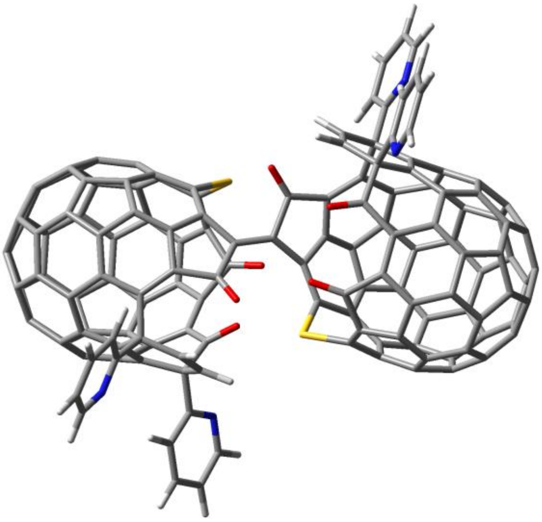

Standard orientation:

Center Number	Atomic Number	Atomic Type	Coordinates (Angstroms) X	Y	Z
1	6	0	-2.669215	-3.454204	1.034740
2	6	0	-2.960091	-3.017259	2.253905
3	6	0	-4.195706	-2.227736	2.604350
4	6	0	-3.931053	-0.726503	2.807497
5	6	0	-2.735351	-0.091629	2.526489
6	6	0	-1.364636	-0.510087	2.028755
7	6	0	-0.815623	0.645077	1.186946
8	6	0	-1.756918	1.762638	1.393214
9	6	0	-2.170002	2.791788	0.547369
10	6	0	-3.429094	3.497447	0.751124
11	6	0	-4.236122	4.046482	-0.359098
12	6	0	-3.920917	3.651867	-1.740962
13	6	0	-2.744500	2.842331	-2.003791
14	6	0	-2.814679	1.789238	-2.892325
15	6	0	-2.153143	0.416235	-2.756375
16	6	0	-3.352367	-0.551164	-2.953940
17	6	0	-3.785425	-1.632171	-2.195313
18	6	0	-2.896270	-2.244956	-1.140521
19	6	0	-3.555242	-3.247081	-0.160379
20	6	0	-4.898810	-2.676615	0.215684
21	6	0	-5.215300	-2.258007	1.479312
22	6	0	-6.452369	-1.554935	1.717602
23	6	0	-6.449166	-0.398660	2.608344
24	6	0	-5.128037	0.087805	3.036056
25	6	0	-4.980946	1.473379	3.166678
26	6	0	-3.771335	2.096161	2.726377
27	6	0	-2.708668	1.326768	2.372991
28	6	0	-4.090030	3.267475	1.967601
29	6	0	-5.484360	3.519984	2.171548
30	6	0	-6.041982	2.406784	2.916076
31	6	0	-7.302511	1.946183	2.626586
32	6	0	-7.509998	0.518099	2.483640
33	6	0	-8.549985	0.358662	1.487522
34	6	0	-8.517072	-0.722238	0.632342
35	6	0	-7.442340	-1.670801	0.748601
36	6	0	-7.098896	-2.107745	-0.585573
37	6	0	-5.780662	-2.408700	-0.891335
38	6	0	-5.210597	-1.950840	-2.161423
39	6	0	-6.082280	-1.306089	-3.051003
40	6	0	-5.619144	-0.207522	-3.848901
41	6	0	-4.304305	0.185947	-3.723495
42	6	0	-3.993201	1.597270	-3.657541
43	6	0	-4.996850	2.536769	-3.673617
44	6	0	-4.958598	3.584253	-2.683200
45	6	0	-6.322220	3.908331	-2.363373
46	6	0	-6.621883	4.319480	-1.086268
47	6	0	-5.573342	4.411250	-0.089201
48	6	0	-6.212424	4.126877	1.181077
49	6	0	-7.584625	3.724341	0.952348
50	6	0	-8.118344	2.648113	1.657367
51	6	0	-8.920038	1.665211	0.967031
52	6	0	-9.216211	1.826240	-0.381311
53	6	0	-9.196089	0.679459	-1.271333
54	6	0	-8.854608	-0.566057	-0.766973
55	6	0	-7.968510	-1.428295	-1.511519
56	6	0	-7.470650	-1.039922	-2.736211
57	6	0	-7.837077	0.253672	-3.280389
58	6	0	-6.679463	0.777572	-3.964545
59	6	0	-6.372852	2.129785	-3.867818
60	6	0	-7.205634	3.003442	-3.077988
61	6	0	-8.342971	2.511222	-2.451552
62	6	0	-8.673942	2.957347	-1.110000
63	6	0	-7.850676	3.867006	-0.457016
64	6	0	-8.668922	1.103194	-2.554089
65	6	0	-3.761056	-4.590737	-0.864548
66	6	0	-4.415245	-5.619876	-0.181663
67	6	0	-4.575027	-6.835277	-0.829635
68	6	0	-4.079154	-6.978176	-2.124461
69	6	0	-3.444183	-5.891549	-2.712395
70	6	0	-4.790315	-2.873523	3.863764
71	6	0	-4.506298	-2.392654	5.141317
72	6	0	-5.023934	-3.073779	6.237481
73	6	0	-5.801693	-4.204367	6.019838
74	6	0	-6.017285	-4.608730	4.705218
75	16	0	-1.312607	3.035846	-0.984709

76	8	0	-1.742788	-1.916917	-0.999911
77	8	0	-0.704789	-1.426331	2.436607
78	8	0	-0.997800	0.183550	-2.546232
79	7	0	-5.523397	-3.968056	3.646169
80	7	0	-3.283139	-4.713223	-2.100514
81	6	0	3.746623	3.266811	2.308024
82	6	0	3.449334	3.738050	1.102736
83	6	0	4.172613	3.371102	-0.167689
84	6	0	3.347117	2.434903	-1.068064
85	6	0	2.120895	1.878394	-0.724163
86	6	0	1.071683	2.032548	0.348187
87	6	0	0.338669	0.682872	0.461752
88	6	0	1.028884	-0.240321	-0.478086
89	6	0	3.402435	0.956046	-3.037609
90	6	0	1.302974	-1.589650	-0.674628
91	6	0	2.107144	-2.053671	-1.809471
92	6	0	2.980548	-3.245301	-1.751547
93	6	0	3.291158	-3.843021	-0.444174
94	6	0	2.622811	-3.331626	0.738123
95	6	0	3.347014	-3.110437	1.894020
96	6	0	3.220016	-1.899307	2.829467
97	6	0	4.695546	-1.466422	3.037654
98	6	0	5.246896	-0.192594	2.982865
99	6	0	4.419724	1.037291	3.227483
100	6	0	4.914962	2.367365	2.595056
101	6	0	5.737767	2.084577	1.364893
102	6	0	5.443568	2.580595	0.122487
103	6	0	6.215691	2.161034	-1.022975
104	6	0	5.526359	1.895309	-2.280729
105	6	0	4.057391	1.891535	-2.225654
106	6	0	1.710670	0.671073	-1.359209
107	6	0	6.180012	1.079952	-3.222648
108	6	0	5.452147	0.120862	-4.034006
109	6	0	4.088806	0.036438	-3.899654
110	6	0	3.447147	-1.257460	-3.764423
111	6	0	2.375672	-1.126808	-2.823267
112	6	0	2.262689	0.266038	-2.527818
113	6	0	4.195168	-2.406106	-3.777945
114	6	0	3.940941	-3.419877	-2.773491
115	6	0	5.216078	-4.070388	-2.542051
116	6	0	5.508866	-4.588085	-1.302935
117	6	0	4.528352	-4.475464	-0.258068
118	6	0	5.246952	-4.344839	0.984141
119	6	0	4.691785	-3.559920	1.968695
120	6	0	5.496902	-2.585368	2.668407
121	6	0	6.837634	-2.466282	2.363081
122	6	0	7.389488	-1.148598	2.229428
123	6	0	6.580010	-0.022349	2.425162
124	6	0	6.801355	1.125424	1.542335
125	6	0	7.743282	1.027503	0.530088
126	6	0	7.445755	1.559749	-0.779171
127	6	0	8.097084	0.722134	-1.750192
128	6	0	7.471496	0.482142	-2.954944
129	6	0	7.508472	-0.857230	-3.519778
130	6	0	6.236528	-1.088919	-4.163075
131	6	0	5.616629	-2.331159	-4.042411
132	6	0	6.253596	-3.385755	-3.294055
133	6	0	7.507793	-3.185267	-2.729538
134	6	0	7.807201	-3.723293	-1.414561
135	6	0	6.833771	-4.437411	-0.727485
136	6	0	6.663948	-4.259162	0.694853
137	6	0	7.450239	-3.338089	1.377394
138	6	0	8.433890	-2.562845	0.658790
139	6	0	8.401113	-1.216751	1.197528
140	6	0	8.572296	-0.139368	0.354839
141	6	0	8.797461	-0.338415	-1.056841
142	6	0	8.822879	-1.621776	-1.583110
143	6	0	8.147591	-1.888003	-2.840728
144	6	0	8.626105	-2.760363	-0.706629
145	6	0	5.843232	3.056365	3.611354
146	6	0	6.328734	4.336866	3.331412
147	6	0	7.181552	4.925611	4.253876
148	6	0	7.518698	4.222354	5.408724
149	6	0	6.991301	2.949020	5.581896
150	6	0	4.547399	4.686783	-0.866176
151	6	0	3.882644	5.162184	-1.994816
152	6	0	4.265723	6.391237	-2.523935
153	6	0	5.291673	7.098225	-1.910920
154	6	0	5.884345	6.542257	-0.779710
155	16	0	0.952308	-2.768813	0.598235
156	8	0	0.776838	3.010881	0.991087
157	8	0	3.418054	1.011700	3.896096
158	8	0	2.215313	-1.358309	3.186622
159	7	0	6.169246	2.368151	4.702630
160	7	0	5.525141	5.370048	-0.261841
161	1	0	-1.739689	-3.985613	0.852968
162	1	0	-2.263284	-3.188123	3.067472
163	1	0	-4.779744	-5.447795	0.828105
164	1	0	-5.077331	-7.660606	-0.335093
165	1	0	-4.181037	-7.911122	-2.667126
166	1	0	-3.042474	-5.962892	-3.719957
167	1	0	-3.894802	-1.505628	5.269696
168	1	0	-4.822510	-2.723552	7.244886
169	1	0	-6.231103	-4.763697	6.843198
170	1	0	-6.615159	-5.491574	4.491248
171	1	0	3.142800	3.543008	3.166622
172	1	0	2.603747	4.404675	0.980994
173	1	0	6.046854	4.830460	2.403962
174	1	0	7.582680	5.918544	4.075701
175	1	0	8.181419	4.646302	6.154702
176	1	0	7.237063	2.361804	6.463101
177	1	0	3.086498	4.582202	-2.448379
178	1	0	3.768545	6.786760	-3.404048
179	1	0	5.626992	8.056353	-2.291103
180	1	0	6.686936	7.065456	-0.265100

The total electronic energy was calculated to be –7115.9729722 Hartree.

Supplementary Table 11. Optimized structure of **5'** (M06-2X/6-31G(d,p))

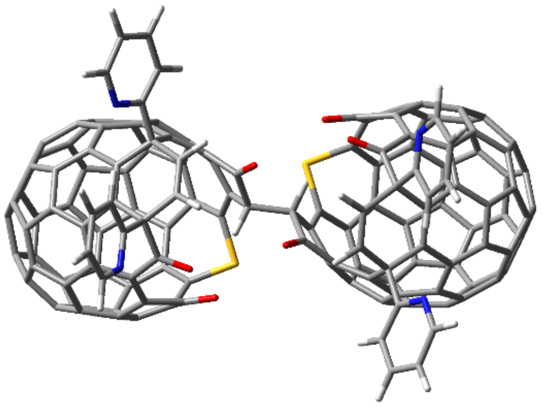

Standard orientation:

Center Number	Atomic Number	Atomic Type	Coordinates (Angstroms) X	Y	Z
1	6	0	-3.296506	3.675203	-0.251172
2	6	0	-3.190411	3.296838	-1.519669
3	6	0	-4.158025	2.382728	-2.226274
4	6	0	-3.557940	0.987222	-2.460556
5	6	0	-2.335738	0.580317	-1.954467
6	6	0	-1.185253	1.197464	-1.165020
7	6	0	-0.763724	0.156263	-0.091483
8	6	0	-1.404262	-1.118522	-0.592692
9	6	0	-4.028789	-1.343961	-3.126730
10	6	0	-1.734335	-2.279678	0.075561
11	6	0	-2.711554	-3.217432	-0.457333
12	6	0	-3.628275	-3.978303	0.414021
13	6	0	-3.737870	-3.613762	1.839563
14	6	0	-2.858715	-2.600293	2.404241
15	6	0	-3.365662	-1.648914	3.268434
16	6	0	-3.010896	-0.159576	3.275696
17	6	0	-4.399050	0.527331	3.312060
18	6	0	-4.875851	1.579669	2.539080
19	6	0	-3.938346	2.554824	1.884631
20	6	0	-4.456033	3.318079	0.636028
21	6	0	-5.499657	2.496659	-0.076487
22	6	0	-5.400072	2.117395	-1.388994
23	6	0	-6.377994	1.220571	-1.958896
24	6	0	-5.933834	0.164676	-2.864441
25	6	0	-4.480611	-0.024373	-2.982434
26	6	0	-2.087699	-0.819621	-1.803983
27	6	0	-6.786512	-0.941437	-3.039850
28	6	0	-6.266410	-2.288706	-3.174181
29	6	0	-4.907215	-2.478680	-3.163995
30	6	0	-4.325958	-3.518121	-2.335702
31	6	0	-3.097550	-3.021432	-1.791700
32	6	0	-2.855112	-1.758511	-2.422640
33	6	0	-5.125057	-4.325245	-1.568414
34	6	0	-4.759621	-4.573102	-0.185345
35	6	0	-6.009630	-4.758676	0.523999
36	6	0	-6.110255	-4.390314	1.844223
37	6	0	-4.959711	-3.824154	2.497087
38	6	0	-5.453142	-2.887685	3.475951
39	6	0	-4.700189	-1.766238	3.733657
40	6	0	-5.316936	-0.459999	3.777919
41	6	0	-6.679372	-0.349251	3.590878
42	6	0	-7.167913	0.696992	2.738797
43	6	0	-6.270863	1.582841	2.126547
44	6	0	-6.569879	2.001109	0.754708
45	6	0	-7.675661	1.466508	0.111784
46	6	0	-7.579128	1.071239	-1.274248
47	6	0	-8.436663	-0.068089	-1.463964
48	6	0	-8.043990	-1.063710	-2.331821
49	6	0	-8.253614	-2.453721	-1.960393
50	6	0	-7.126650	-3.207255	-2.458716
51	6	0	-6.564961	-4.211751	-1.673161
52	6	0	-7.121113	-4.508731	-0.377113
53	6	0	-8.246495	-3.830979	0.075148
54	6	0	-8.344368	-3.435049	1.468339
55	6	0	-7.308650	-3.738406	2.342542
56	6	0	-6.891221	-2.782776	3.338929
57	6	0	-7.497485	-1.533615	3.400858
58	6	0	-8.543442	-1.197062	2.463869
59	6	0	-8.342312	0.183561	2.068449
60	6	0	-8.589774	0.563270	0.766393
61	6	0	-9.066584	-0.394798	-0.201851
62	6	0	-9.255675	-1.719190	0.165138
63	6	0	-8.823508	-2.774290	-0.734045
64	6	0	-8.976495	-2.132443	1.526997
65	6	0	-5.139172	4.610833	1.113947
66	6	0	-5.617643	5.515927	0.161856
67	6	0	-6.240233	6.669499	0.615271
68	6	0	-6.364670	6.875487	1.988044
69	6	0	-5.867159	5.904311	2.847557
70	6	0	-4.556190	3.073761	-3.537417
71	6	0	-3.961304	2.752905	-4.756365
72	6	0	-4.332574	3.473466	-5.886996
73	6	0	-5.279912	4.481555	-5.761209
74	6	0	-5.806947	4.731309	-4.496801
75	6	0	3.296622	3.675290	0.251121
76	6	0	3.190437	3.296888	1.519599
77	6	0	4.157970	2.382712	2.226229
78	6	0	3.557836	0.987213	2.460450
79	6	0	2.335646	0.580327	1.954302
80	6	0	1.185179	1.197480	1.164826
81	6	0	0.763677	0.156277	0.091278
82	6	0	1.404204	-1.118502	0.592496
83	6	0	1.734279	-2.279665	-0.075741
84	6	0	2.711460	-3.217434	0.457194
85	6	0	3.628207	-3.978315	-0.414122
86	6	0	3.737870	-3.613763	-1.839656
87	6	0	2.858750	-2.600276	-2.404365
88	6	0	3.365764	-1.648872	-3.268491
89	6	0	3.011045	-0.159523	-3.275680
90	6	0	4.399209	0.527369	-3.312039
91	6	0	4.876015	1.579714	-2.539072
92	6	0	3.938521	2.554916	-1.884673
93	6	0	4.456175	3.318131	-0.636032
94	6	0	5.499729	2.496659	0.076521
95	6	0	5.400057	2.117365	1.389013
96	6	0	6.377928	1.220511	1.958953
97	6	0	5.933695	0.164617	2.864462
98	6	0	4.480460	-0.024404	2.982373
99	6	0	4.028610	-1.343984	3.126648
100	6	0	2.854959	-1.758513	2.422506
101	6	0	2.087597	-0.819607	1.803811
102	6	0	3.097400	-3.021440	1.791578

103	6	0	4.325774	-3.518151	2.335634
104	6	0	4.907011	-2.478721	3.163956
105	6	0	6.266208	-2.288773	3.174209
106	6	0	6.786342	-0.941513	3.039910
107	6	0	8.043855	-1.063807	2.331946
108	6	0	8.436594	-0.068188	1.464117
109	6	0	7.579096	1.071161	1.274368
110	6	0	7.675711	1.466448	-0.111654
111	6	0	6.569978	2.001091	-0.754627
112	6	0	6.271012	1.582843	-2.126483
113	6	0	7.168060	0.696965	-2.738693
114	6	0	6.679521	-0.349266	-3.590790
115	6	0	5.317088	-0.459982	-3.777870
116	6	0	4.700312	-1.766209	-3.733652
117	6	0	5.453232	-2.887676	-3.475944
118	6	0	4.959741	-3.824158	-2.497123
119	6	0	6.110248	-4.390342	-1.844212
120	6	0	6.009558	-4.758718	-0.523996
121	6	0	4.759518	-4.573132	0.185292
122	6	0	5.124895	-4.325287	1.568379
123	6	0	6.564796	-4.211816	1.673193
124	6	0	7.126465	-3.207336	2.458781
125	6	0	8.253468	-2.453819	1.960518
126	6	0	8.823415	-2.774390	0.734195
127	6	0	9.255647	-1.719293	-0.164960
128	6	0	9.066571	-0.394899	0.202032
129	6	0	8.589830	0.563190	-0.766227
130	6	0	8.342416	0.183497	-2.068297
131	6	0	8.543527	-1.197129	-2.463717
132	6	0	7.497600	-1.533648	-3.400751
133	6	0	6.891307	-2.782798	-3.338861
134	6	0	7.308675	-3.738447	-2.342469
135	6	0	8.344361	-3.435121	-1.468217
136	6	0	8.246418	-3.831061	-0.075034
137	6	0	7.121004	-4.508798	0.377169
138	6	0	8.976518	-2.132528	-1.526834
139	6	0	5.139386	4.610866	-1.113896
140	6	0	5.617789	5.515950	-0.161762
141	6	0	6.240438	6.669513	-0.615119
142	6	0	6.365015	6.875493	-1.987881
143	6	0	5.867571	5.904323	-2.847439
144	6	0	4.556091	3.073700	3.537409
145	6	0	3.961124	2.752848	4.756318
146	6	0	4.332368	3.473369	5.886983
147	6	0	5.279765	4.481411	5.761268
148	6	0	5.806881	4.731164	4.496894
149	16	0	1.215024	-2.429152	-1.768262
150	16	0	-1.215020	-2.429169	1.768064
151	8	0	2.824038	2.742386	-2.302566
152	8	0	0.569519	2.199500	1.401408
153	8	0	-0.569676	2.199547	-1.401551
154	8	0	1.918982	0.321328	-3.154904
155	8	0	-2.823925	2.742437	2.302624
156	8	0	-1.918882	0.321242	3.154340
157	7	0	5.455978	4.052788	3.405373
158	7	0	5.266215	4.787177	-2.426405
159	7	0	-5.265875	4.787148	2.426467
160	7	0	-5.456020	4.052892	-3.405313
161	1	0	-2.521996	4.283478	0.205431
162	1	0	-2.324942	3.598409	-2.100604
163	1	0	-1.226080	0.465226	0.858757
164	1	0	-5.507791	5.294334	-0.897519
165	1	0	-6.626149	7.398527	-0.090319
166	1	0	-6.840935	7.764808	2.385069
167	1	0	-5.952662	6.020738	3.925134
168	1	0	-3.223757	1.959811	-4.815562
169	1	0	-3.886325	3.248331	-6.850462
170	1	0	-5.601996	5.065996	-6.615470
171	1	0	-6.546774	5.515338	-4.353502
172	1	0	2.522173	4.283630	-0.205498
173	1	0	2.324959	3.598492	2.100501
174	1	0	1.226025	0.465247	-0.858962
175	1	0	5.507823	5.294365	0.897602
176	1	0	6.626288	7.398541	0.090507
177	1	0	6.841326	7.764808	-2.384862
178	1	0	5.953187	6.020743	-3.925007
179	1	0	3.223536	1.959787	4.815459
180	1	0	3.886056	3.248236	6.850421
181	1	0	5.601832	5.065818	6.615558
182	1	0	6.546754	5.515159	4.353650

The total electronic energy was calculated to be –7117.2240598 Hartree.

Supplementary Table 12. Optimized structure of **6'** (M06-2X/6-31G(d,p))

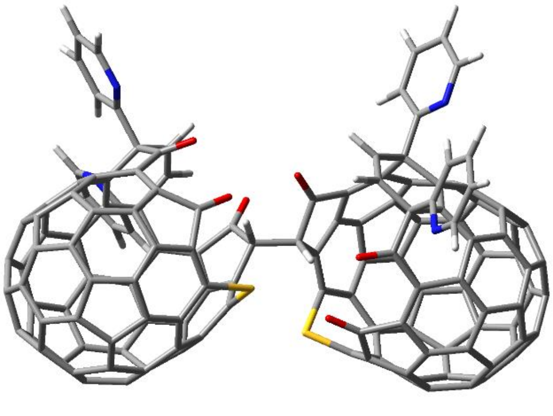

Standard orientation:

Center Number	Atomic Number	Atomic Type	Coordinates (Angstroms) X	Y	Z
1	6	0	-3.509415	3.446146	-1.528946
2	6	0	-3.380927	2.660302	-2.591446
3	6	0	-4.296936	1.512531	-2.927150
4	6	0	-3.627900	0.149498	-2.684142
5	6	0	-2.383020	-0.012805	-2.094921
6	6	0	-1.270984	0.881845	-1.564791
7	6	0	-0.723125	0.231032	-0.270066
8	6	0	-1.342687	-1.143685	-0.284202
9	6	0	-3.991356	-2.288828	-2.533317
10	6	0	-1.630062	-2.013205	0.746935
11	6	0	-2.573840	-3.105575	0.573360
12	6	0	-3.453646	-3.561843	1.665970
13	6	0	-3.574430	-2.735856	2.881187

14	6	0	-2.740167	-1.551205	3.044257
15	6	0	-3.292609	-0.389119	3.549171
16	6	0	-3.017577	1.029215	3.045221
17	6	0	-4.439113	1.628882	2.894391
18	6	0	-4.972783	2.343957	1.829229
19	6	0	-4.094318	3.099338	0.873845
20	6	0	-4.648768	3.359542	-0.554013
21	6	0	-5.646521	2.293351	-0.925843
22	6	0	-5.526872	1.493282	-2.031183
23	6	0	-6.459742	0.413995	-2.248303
24	6	0	-5.964794	-0.862666	-2.754335
25	6	0	-4.503772	-1.017093	-2.825102
26	6	0	-2.060181	-1.278689	-1.505990
27	6	0	-6.764159	-2.000213	-2.537239
28	6	0	-6.182110	-3.291265	-2.224053
29	6	0	-4.815438	-3.409037	-2.177258
30	6	0	-4.181234	-4.087832	-1.063354
31	6	0	-2.972498	-3.388221	-0.742556
32	6	0	-2.792774	-2.398505	-1.761729
33	6	0	-4.939580	-4.621152	-0.053536
34	6	0	-4.557904	-4.372668	1.325304
35	6	0	-5.793839	-4.361040	2.080232
36	6	0	-5.904930	-3.571972	3.200021
37	6	0	-4.780715	-2.766632	3.597021
38	6	0	-5.314569	-1.578161	4.214368
39	6	0	-4.616524	-0.403860	4.058961
40	6	0	-5.301249	0.811565	3.681960
41	6	0	-6.669377	0.791743	3.501231
42	6	0	-7.214862	1.469984	2.359878
43	6	0	-6.367728	2.141853	1.468748
44	6	0	-6.690612	2.059665	0.042199
45	6	0	-7.767966	1.286550	-0.362710
46	6	0	-7.653081	0.451195	-1.534853
47	6	0	-8.453337	-0.723757	-1.315851
48	6	0	-8.014007	-1.933668	-1.807284
49	6	0	-8.155445	-3.126305	-0.988144
50	6	0	-6.996141	-3.953719	-1.227333
51	6	0	-6.383529	-4.609923	-0.161348
52	6	0	-6.919806	-4.478393	1.170115
53	6	0	-8.074448	-3.737880	1.390827
54	6	0	-8.185154	-2.900669	2.572199
55	6	0	-7.131096	-2.844352	3.475041
56	6	0	-6.757108	-1.590879	4.082427
57	6	0	-7.427475	-0.423205	3.736410
58	6	0	-8.492466	-0.470044	2.762210
59	6	0	-8.363018	0.706057	1.923026
60	6	0	-8.634587	0.614740	0.574499
61	6	0	-9.064777	-0.634819	-0.006616
62	6	0	-9.185538	-1.766261	0.787005
63	6	0	-8.705025	-3.041439	0.285661
64	6	0	-8.881097	-1.684754	2.202498
65	6	0	-5.402952	4.700487	-0.539975
66	6	0	-5.905323	5.208872	-1.741487
67	6	0	-6.598728	6.409482	-1.701747
68	6	0	-6.767720	7.052659	-0.477075
69	6	0	-6.240212	6.453054	0.659421
70	6	0	-4.731821	1.699387	-4.388025
71	6	0	-4.192374	0.957012	-5.436035
72	6	0	-4.605317	1.240799	-6.734774
73	6	0	-5.536421	2.249855	-6.939712
74	6	0	-6.008469	2.940646	-5.825677
75	16	0	-1.099411	-1.553534	2.378107
76	8	0	-0.743866	1.829703	-2.090914
77	8	0	-3.003446	3.500542	1.190302
78	8	0	-1.960207	1.490350	2.722731
79	7	0	-5.570779	5.296277	0.637958
80	7	0	-5.618711	2.682205	-4.579779
81	6	0	3.092491	2.180285	2.942847
82	6	0	3.124016	3.082226	1.968818
83	6	0	4.200310	3.174543	0.917961
84	6	0	3.718032	2.671659	-0.453049
85	6	0	2.492183	2.064386	-0.668584
86	6	0	1.253809	1.741624	0.153233
87	6	0	0.809118	0.301064	-0.234839
88	6	0	1.574303	0.046519	-1.520603
89	6	0	4.403920	2.024882	-2.734262
90	6	0	1.928467	-1.126378	-2.152083
91	6	0	3.010409	-1.179648	-3.128214
92	6	0	3.903569	-2.348516	-3.251380
93	6	0	3.869676	-3.395364	-2.212337
94	6	0	2.873145	-3.337062	-1.154047
95	6	0	3.236358	-3.605528	0.151550
96	6	0	2.776923	-2.822346	1.379854
97	6	0	4.098748	-2.546748	2.137548
98	6	0	4.558474	-1.358990	2.696431
99	6	0	3.610613	-0.260336	3.089670
100	6	0	4.184005	1.178398	3.193223
101	6	0	5.341345	1.335913	2.240784
102	6	0	5.382862	2.274367	1.244084
103	6	0	6.465253	2.266893	0.288693
104	6	0	6.171331	2.516789	-1.119739
105	6	0	4.749105	2.566239	-1.488428
106	6	0	2.332098	1.217934	-1.809502
107	6	0	7.111057	2.071227	-2.068284
108	6	0	6.699872	1.512716	-3.342525
109	6	0	5.360727	1.449998	-3.637052
110	6	0	4.789773	0.227067	-4.168229
111	6	0	3.489860	0.053692	-3.591451
112	6	0	3.209733	1.246304	-2.849520
113	6	0	5.579497	-0.870892	-4.390579
114	6	0	5.121218	-2.173414	-3.944641
115	6	0	6.319514	-2.919541	-3.615281
116	6	0	6.285320	-3.869583	-2.622964
117	6	0	5.046797	-4.105657	-1.930371
118	6	0	5.387439	-4.481564	-0.580137
119	6	0	4.534146	-4.104151	0.429998
120	6	0	5.045655	-3.487323	1.632661
121	6	0	6.406960	-3.315725	1.777624
122	6	0	6.885654	-2.064738	2.292240
123	6	0	5.980812	-1.054214	2.644633
124	6	0	6.367411	0.327160	2.345720
125	6	0	7.560101	0.565997	1.680551
126	6	0	7.611438	1.558379	0.632010
127	6	0	8.558953	1.106646	-0.351261
128	6	0	8.311386	1.356634	-1.683839
129	6	0	8.587096	0.318188	-2.663196
130	6	0	7.562022	0.398570	-3.677196
131	6	0	7.011361	-0.770588	-4.198275
132	6	0	7.479904	-2.054348	-3.740653
133	6	0	8.512331	-2.134230	-2.814053
134	6	0	8.466249	-3.132185	-1.760180
135	6	0	7.387044	-4.003315	-1.686333
136	6	0	6.820209	-4.355795	-0.407720
137	6	0	7.324484	-3.788419	0.756420
138	6	0	8.416966	-2.847176	0.680111
139	6	0	8.148186	-1.793927	1.640287
140	6	0	8.477248	-0.491006	1.333267
141	6	0	9.101807	-0.166465	0.073248
142	6	0	9.354763	-1.165786	-0.854922
143	6	0	9.074921	-0.918707	-2.258056

144	6	0	8.993954	-2.536842	-0.548986
145	6	0	4.729717	1.373001	4.617784
146	6	0	5.226764	2.625014	4.990607
147	6	0	5.726239	2.769764	6.276640
148	6	0	5.714465	1.671444	7.134447
149	6	0	5.208302	0.468700	6.658377
150	6	0	4.662147	4.637750	0.877384
151	6	0	4.228112	5.530745	-0.100718
152	6	0	4.641937	6.856788	-0.021078
153	6	0	5.470625	7.240888	1.025075
154	6	0	5.840754	6.273921	1.956326
155	16	0	1.281151	-2.646859	-1.502132
156	8	0	0.615244	2.456975	0.874310
157	8	0	2.443727	-0.473147	3.306317
158	8	0	1.667700	-2.419916	1.599397
159	7	0	4.724495	0.312890	5.421859
160	7	0	5.447065	5.003138	1.895413
161	1	0	-2.770538	4.214751	-1.324679
162	1	0	-2.534309	2.790176	-3.257043
163	1	0	-1.074620	0.822598	0.590511
164	1	0	-5.760979	4.654966	-2.666344
165	1	0	-7.006066	6.837980	-2.612173
166	1	0	-7.301721	7.993233	-0.402075
167	1	0	-6.358667	6.914666	1.636519
168	1	0	-3.466661	0.175429	-5.240965
169	1	0	-4.202160	0.678626	-7.571261
170	1	0	-5.889960	2.503150	-7.932602
171	1	0	-6.735255	3.741227	-5.942760
172	1	0	2.246383	2.141244	3.621720
173	1	0	2.299649	3.778169	1.855316
174	1	0	1.161936	-0.370728	0.562626
175	1	0	5.225396	3.442635	4.273201
176	1	0	6.121486	3.724755	6.608255
177	1	0	6.093811	1.742999	8.147492
178	1	0	5.188985	-0.415191	7.290728
179	1	0	3.579536	5.194509	-0.902146
180	1	0	4.317786	7.576220	-0.766392
181	1	0	5.820721	8.261765	1.126048
182	1	0	6.481250	6.534653	2.795656

The total electronic energy was calculated to be –7117.2267101 Hartree.

Molecular Orbitals and Optical Transitions

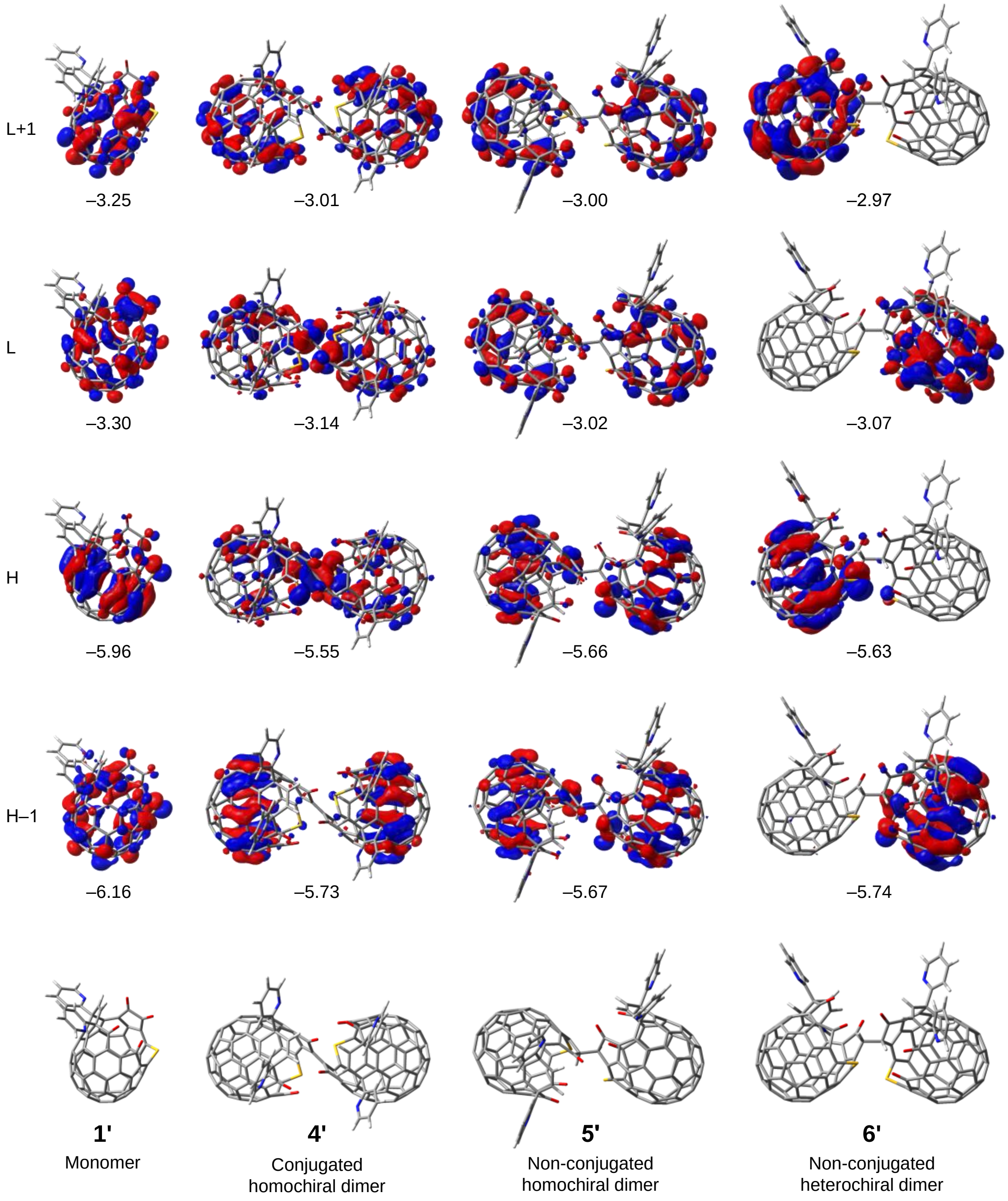

Supplementary Figure 30. Molecular orbitals (MOs) of **1'**, **4'**, **5'**, and **6'** (B3LYP-D3/6-31G(d,p)). Energy levels were shown with units in eV.

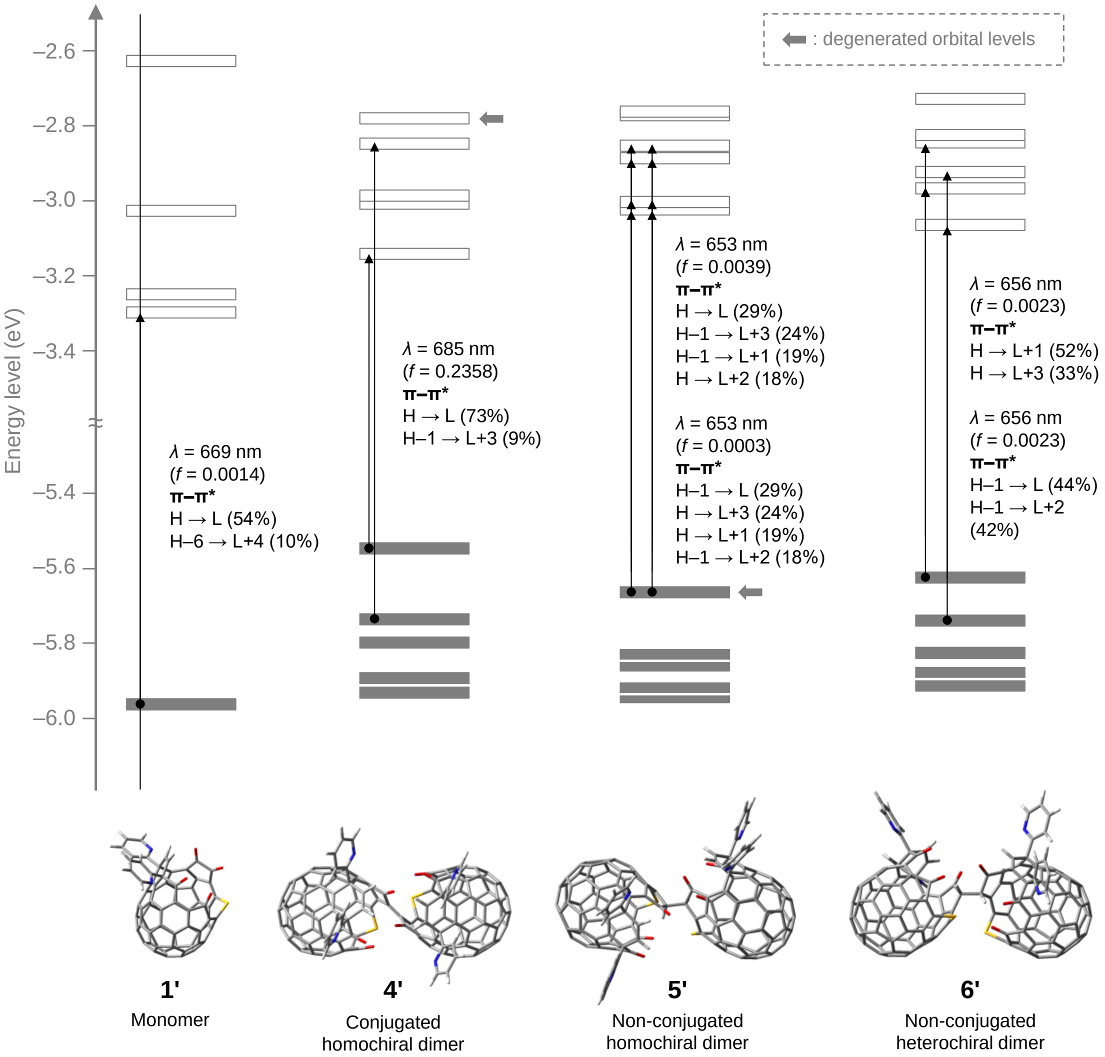

Supplementary Figure 31. Optical transitions of **1'**, **4'**, **5'**, and **6'** (TD CAM-B3LYP-D3/6-31G(d,p)) with the Kohn-Sham MO levels (B3LYP-D3/6-31G(d,p)). The transition energies were calibrated with a factor of 0.72 (*26*).

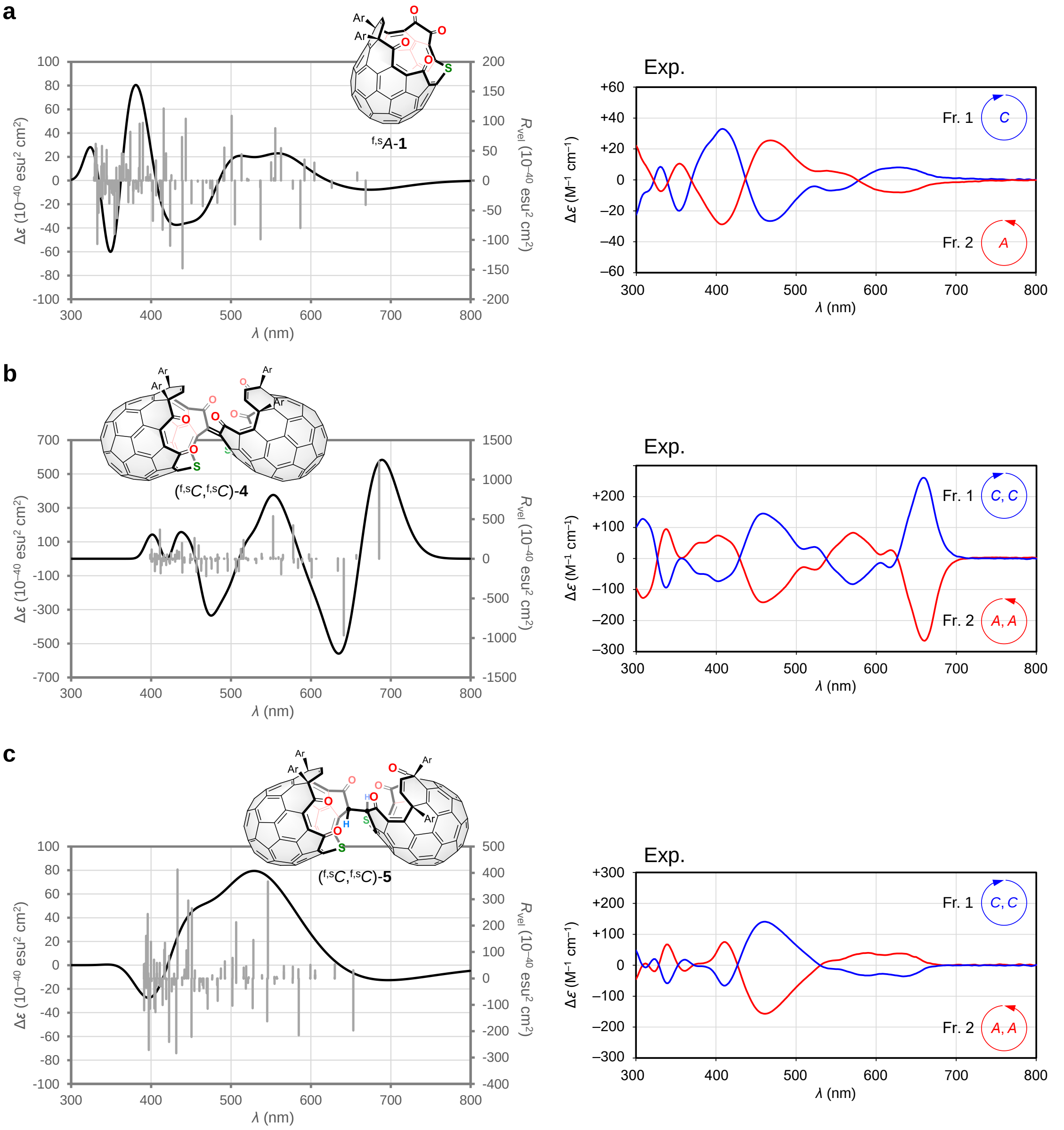

Supplementary Figure 32. Simulated and measured CD spectra. a. 1' (calc.) and **1** (exp.). **b. 4'** (calc.) and **4** (exp.). **c. 5'** (calc.) and **5** (exp.). The calculations were conducted at the TD CAM-B3LYP-D3/6-31G(d,p)//B3LYP-D3/6-31G(d,p) level of theory.

Supplementary Table 13. Optimized structure of **1'** (B3LYP-D3/6-31G(d,p))

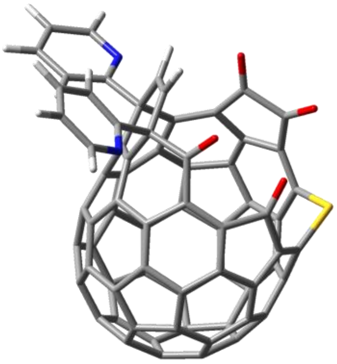

Standard orientation:

Center Number	Atomic Number	Atomic Type	X	Y	Z
1	6	0	3.797387	0.310062	1.477656
2	6	0	3.806230	-0.941116	1.024364
3	6	0	3.001555	-1.438814	-0.151358
4	6	0	1.831353	-2.352310	0.284154
5	6	0	1.477380	-2.590987	1.611571
6	6	0	1.999092	-2.235777	2.986455
7	6	0	0.720767	-2.044770	3.902347
8	6	0	-0.432439	-2.461195	3.066351
9	6	0	-1.767851	-2.072916	3.052502
10	6	0	-2.863730	0.303712	3.060047
11	6	0	-2.262187	1.556218	2.982748
12	6	0	-0.786830	1.874999	3.275452
13	6	0	-0.369801	2.746936	2.066085
14	6	0	0.791505	2.655771	1.294091
15	6	0	2.073773	2.104131	1.835571
16	6	0	3.067504	1.445412	0.813448
17	16	0	-2.290274	-0.874585	4.263840
18	7	0	3.670316	3.803646	0.493796
19	8	0	3.120042	-2.195460	3.433391
20	8	0	2.365321	2.171928	3.009329
21	7	0	4.059199	-3.530833	-0.848328
22	6	0	2.326270	1.005921	-0.428703
23	6	0	1.508743	-0.652622	-2.031358
24	8	0	0.760376	-1.660166	5.046035
25	6	0	1.455663	1.994948	-1.019656
26	6	0	0.897794	-2.724309	-0.784291
27	8	0	-0.099044	1.417477	4.151645
28	6	0	-0.282825	-2.101277	-2.890171
29	6	0	4.940123	-4.228585	-1.577074
30	6	0	-1.445303	-2.873520	-2.491795
31	6	0	3.972416	-2.213747	-1.063041
32	6	0	0.835049	-1.948527	-2.032050
33	6	0	1.002170	0.386772	-2.819574
34	6	0	2.336457	-0.288282	-0.904982
35	6	0	-1.434720	-3.505614	-1.265688
36	6	0	-0.143363	0.217376	-3.676040
37	6	0	-0.679384	-3.522863	0.907177
38	6	0	-0.781692	-1.010995	-3.706869
39	6	0	0.966680	1.734777	-2.298695
40	6	0	0.701536	2.890130	-0.140341
41	6	0	-0.248299	-3.477418	-0.456892
42	6	0	5.329045	2.182183	-0.135917
43	6	0	4.079364	2.534742	0.386364
44	6	0	-3.683006	-2.651027	-0.755346
45	6	0	-0.452194	3.500156	-0.668581
46	6	0	-0.210880	2.383366	-2.827476
47	6	0	6.184418	3.197769	-0.556019
48	6	0	0.133533	-3.031156	1.886901
49	6	0	-2.236505	-1.057119	-3.732803
50	6	0	5.768466	-3.655450	-2.541077
51	6	0	-4.271299	-1.714080	0.187806
52	6	0	-2.980065	0.125615	-3.696273
53	6	0	-2.093208	-3.283962	0.965054
54	6	0	5.767125	4.523942	-0.441132
55	6	0	4.767668	-1.542158	-1.999876
56	6	0	-2.620712	-2.378485	1.914760
57	6	0	4.502022	4.771588	0.090145
58	6	0	-0.908460	1.449576	-3.681630
59	6	0	-4.828235	-0.642690	-0.613833
60	6	0	-4.354549	0.890889	1.212210
61	6	0	-2.580679	-3.387107	-0.378312
62	6	0	-1.618905	3.671914	0.154402
63	6	0	-2.645549	-2.200205	-2.946545
64	6	0	-4.135190	0.227056	-2.819756
65	6	0	-3.715106	-1.491389	1.475589
66	6	0	5.678225	-2.279045	-2.751890
67	6	0	-1.575209	3.204356	1.459328
68	6	0	-3.790090	-0.122248	2.017316
69	6	0	-4.488688	-0.856580	-2.012229
70	6	0	-3.752804	-2.096453	-2.092610
71	6	0	-2.705161	2.480087	1.997880
72	6	0	-2.303627	1.412138	-3.685313
73	6	0	-3.049569	2.315172	-2.826592
74	6	0	-0.916037	3.260546	-2.018455
75	6	0	-4.566334	1.783921	-0.958773
76	6	0	-2.368607	3.233955	-2.018161
77	6	0	-2.805085	3.477248	-0.660446
78	6	0	-3.813273	2.229820	1.209815
79	6	0	-4.870076	0.643088	-0.109099
80	6	0	-3.886203	2.759948	-0.138838
81	6	0	-4.177110	1.578095	-2.284814
82	1	0	4.353986	0.571638	2.370904
83	1	0	4.369151	-1.701697	1.550038
84	1	0	4.981874	-5.297319	-1.377231
85	1	0	5.615413	1.138392	-0.201481
86	1	0	7.161677	2.957474	-0.964367
87	1	0	6.461973	-4.269771	-3.105696
88	1	0	6.402671	5.346111	-0.752876
89	1	0	4.665842	-0.470285	-2.133385
90	1	0	4.135621	5.790130	0.198531
91	1	0	6.305155	-1.788448	-3.490632

The total electronic energy was calculated to be –3634.4502866 Hartree.

Supplementary Table 14. Optimized structure of **4'** (B3LYP-D3/6-31G(d,p))

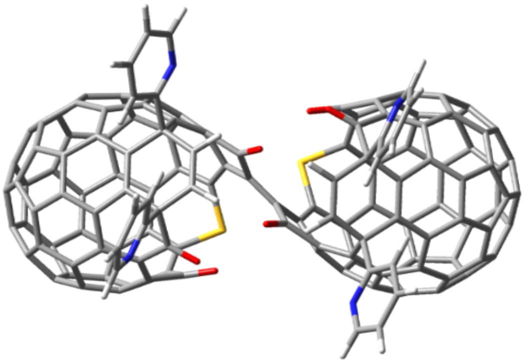

Standard orientation:

Center Number	Atomic Number	Atomic Type	Coordinates (Angstroms) X	Y	Z
1	6	0	-0.267586	-2.576059	3.322425
2	6	0	-1.556784	-2.326673	3.104572
3	6	0	-2.527566	-3.286718	2.460178
4	6	0	-2.928064	-2.828932	1.037694
5	6	0	-2.366999	-1.735955	0.387988
6	6	0	-1.381984	-0.635852	0.726292
7	6	0	-0.547281	-0.404564	-0.546982
8	6	0	-1.203309	-1.157487	-1.609817
9	6	0	-4.005464	-3.492965	-1.089774
10	6	0	-0.721346	-1.782063	-2.770191
11	6	0	-1.491724	-2.810305	-3.458529
12	6	0	-0.863819	-3.938637	-4.178983
13	6	0	0.572839	-4.212400	-3.990044
14	6	0	1.387503	-3.309562	-3.190410
15	6	0	2.317162	-3.813917	-2.287426
16	6	0	2.576097	-3.299497	-0.861936
17	6	0	2.578273	-4.595376	-0.020625
18	6	0	1.931730	-4.832299	1.194287
19	6	0	1.645045	-3.745942	2.182772
20	6	0	0.381632	-3.914822	3.098921
21	6	0	-0.576186	-4.931132	2.513443
22	6	0	-1.909334	-4.670597	2.270308
23	6	0	-2.733864	-5.653863	1.604859
24	6	0	-3.730050	-5.213655	0.629461
25	6	0	-3.704540	-3.793082	0.255157
26	6	0	-2.413396	-1.681978	-1.042132
27	6	0	-4.191610	-6.171033	-0.308505
28	6	0	-4.470284	-5.809552	-1.686368
29	6	0	-4.320225	-4.493429	-2.070196
30	6	0	-3.591189	-4.168809	-3.286842
31	6	0	-2.834653	-2.976667	-3.047854
32	6	0	-3.243035	-2.485026	-1.762654
33	6	0	-3.054094	-5.170911	-4.065190
34	6	0	-1.686599	-5.042037	-4.536651
35	6	0	-1.173971	-6.395354	-4.637457
36	6	0	0.169952	-6.642420	-4.434255
37	6	0	1.037847	-5.538795	-4.119557
38	6	0	2.078478	-6.050218	-3.258872
39	6	0	2.592383	-5.208583	-2.289682
40	6	0	2.760191	-5.675753	-0.932293
41	6	0	2.426702	-6.983113	-0.611117
42	6	0	1.686599	-7.217554	0.599601
43	6	0	1.335565	-6.137750	1.432289
44	6	0	0.017412	-6.170456	2.067923
45	6	0	-0.845183	-7.225437	1.773385
46	6	0	-2.248479	-6.965116	1.547544
47	6	0	-2.722802	-7.922013	0.580701
48	6	0	-3.676883	-7.526669	-0.341538
49	6	0	-3.552450	-7.959783	-1.726097
50	6	0	-4.015025	-6.873243	-2.560594
51	6	0	-3.322017	-6.557507	-3.737303
52	6	0	-2.162963	-7.328315	-4.120046
53	6	0	-1.756877	-8.423025	-3.352297
54	6	0	-0.341935	-8.674644	-3.134049
55	6	0	0.607214	-7.815532	-3.693345
56	6	0	1.782401	-7.435370	-2.943676
57	6	0	1.956224	-7.898814	-1.635768
58	6	0	0.952384	-8.750692	-1.035867
59	6	0	0.797694	-8.330786	0.346236
60	6	0	-0.459152	-8.334153	0.930769
61	6	0	-1.616491	-8.771274	0.183518
62	6	0	-1.480094	-9.173634	-1.146162
63	6	0	-2.465087	-8.742904	-2.124354
64	6	0	-0.169951	-9.149622	-1.773112
65	6	0	0.879036	-4.485497	4.449576
66	6	0	0.158167	-4.272959	5.630289
67	6	0	0.619854	-4.858417	6.806898
68	6	0	1.779976	-5.631635	6.768234
69	6	0	2.424806	-5.786441	5.541519
70	6	0	-3.767305	-3.361806	3.372283
71	6	0	-3.765086	-4.179691	4.509889
72	6	0	-4.880324	-4.168456	5.342010
73	6	0	-5.955475	-3.340197	5.016276
74	6	0	-5.857216	-2.557462	3.866908
75	6	0	0.267586	2.576059	3.322425
76	6	0	1.556784	2.326673	3.104572
77	6	0	2.527566	3.286718	2.460178
78	6	0	2.928064	2.828932	1.037694
79	6	0	2.366999	1.735955	0.387988
80	6	0	1.381984	0.635852	0.726292
81	6	0	0.547281	0.404564	-0.546982
82	6	0	1.203309	1.157487	-1.609817
83	6	0	0.721346	1.782063	-2.770191
84	6	0	1.491724	2.810305	-3.458529
85	6	0	0.863819	3.938637	-4.178983
86	6	0	-0.572839	4.212400	-3.990044
87	6	0	-1.387503	3.309562	-3.190410
88	6	0	-2.317162	3.813917	-2.287426
89	6	0	-2.576097	3.299497	-0.861936
90	6	0	-2.578273	4.595376	-0.020625
91	6	0	-1.931730	4.832299	1.194287
92	6	0	-1.645045	3.745942	2.182772
93	6	0	-0.381632	3.914822	3.098921
94	6	0	0.576186	4.931132	2.513443
95	6	0	1.909334	4.670597	2.270308
96	6	0	2.733864	5.653863	1.604859
97	6	0	3.730050	5.213655	0.629461
98	6	0	3.704540	3.793082	0.255157
99	6	0	4.005464	3.492965	-1.089774
100	6	0	3.243035	2.485026	-1.762654
101	6	0	2.413396	1.681978	-1.042132
102	6	0	2.834653	2.976667	-3.047854
103	6	0	3.591189	4.168809	-3.286842
104	6	0	4.320225	4.493429	-2.070196

105	6	0	4.470284	5.809552	-1.686368
106	6	0	4.191610	6.171033	-0.308505
107	6	0	3.676883	7.526669	-0.341538
108	6	0	2.722802	7.922013	0.580701
109	6	0	2.248479	6.965116	1.547544
110	6	0	0.845183	7.225437	1.773385
111	6	0	-0.017412	6.170456	2.067923
112	6	0	-1.335565	6.137750	1.432289
113	6	0	-1.686599	7.217554	0.599601
114	6	0	-2.426702	6.983113	-0.611117
115	6	0	-2.760191	5.675753	-0.932293
116	6	0	-2.592383	5.208583	-2.289682
117	6	0	-2.078478	6.050218	-3.258872
118	6	0	-1.037847	5.538795	-4.119557
119	6	0	-0.169952	6.642420	-4.434255
120	6	0	1.173971	6.395354	-4.637457
121	6	0	1.686599	5.042037	-4.536651
122	6	0	3.054094	5.170911	-4.065190
123	6	0	3.322017	6.557507	-3.737303
124	6	0	4.015025	6.873243	-2.560594
125	6	0	3.552450	7.959783	-1.726097
126	6	0	2.465087	8.742904	-2.124354
127	6	0	1.480094	9.173634	-1.146162
128	6	0	1.616491	8.771274	0.183518
129	6	0	0.459152	8.334153	0.930769
130	6	0	-0.797694	8.330786	0.346236
131	6	0	-0.952384	8.750692	-1.035867
132	6	0	-1.956224	7.898814	-1.635768
133	6	0	-1.782401	7.435370	-2.943676
134	6	0	-0.607214	7.815532	-3.693345
135	6	0	0.341935	8.674644	-3.134049
136	6	0	1.756877	8.423025	-3.352297
137	6	0	2.162963	7.328315	-4.120046
138	6	0	0.169951	9.149622	-1.773112
139	6	0	-0.879036	4.485497	4.449576
140	6	0	-0.158167	4.272959	5.630289
141	6	0	-0.619854	4.858417	6.806898
142	6	0	-1.779976	5.631635	6.768234
143	6	0	-2.424806	5.786441	5.541519
144	6	0	3.767305	3.361806	3.372283
145	6	0	3.765086	4.179691	4.509889
146	6	0	4.880324	4.168456	5.342010
147	6	0	5.955475	3.340197	5.016276
148	6	0	5.857216	2.557462	3.866908
149	16	0	-1.001974	1.579092	-3.180102
150	16	0	1.001974	-1.579092	-3.180102
151	8	0	-2.377108	2.791129	2.317715
152	8	0	1.370452	-0.091311	1.691620
153	8	0	-1.370452	0.091311	1.691620
154	8	0	-2.616075	2.153068	-0.488650
155	8	0	2.377108	-2.791129	2.317715
156	8	0	2.616075	-2.153068	-0.488650
157	7	0	4.790489	2.561805	3.056965
158	7	0	-1.988575	5.232169	4.404749
159	7	0	1.988575	-5.232169	4.404749
160	7	0	-4.790489	-2.561805	3.056965
161	1	0	0.385248	-1.798992	3.703943
162	1	0	-1.959932	-1.345252	3.317953
163	1	0	-0.736090	-3.660465	5.617419
164	1	0	0.082479	-4.711044	7.739257
165	1	0	2.176692	-6.102973	7.661370
166	1	0	3.332296	-6.381590	5.463636
167	1	0	-2.907892	-4.808911	4.725760
168	1	0	-4.911357	-4.795881	6.228070
169	1	0	-6.846214	-3.300309	5.634629
170	1	0	-6.669991	-1.894155	3.578189
171	1	0	-0.385248	1.798992	3.703943
172	1	0	1.959932	1.345252	3.317953
173	1	0	0.736090	3.660465	5.617419
174	1	0	-0.082479	4.711044	7.739257
175	1	0	-2.176692	6.102973	7.661370
176	1	0	-3.332296	6.381590	5.463636
177	1	0	2.907892	4.808911	4.725760
178	1	0	4.911357	4.795881	6.228070
179	1	0	6.846214	3.300309	5.634629
180	1	0	6.669991	1.894155	3.578189

The total electronic energy was calculated to be –7118.4961326 Hartree.

Supplementary Table 15. Optimized structure of **5'** (B3LYP-D3/6-31G(d,p))

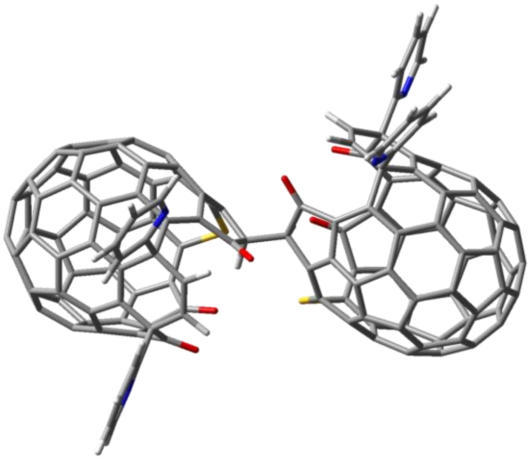

Standard orientation:

Center Number	Atomic Number	Atomic Type	Coordinates (Angstroms) X	Y	Z
1	6	0	-2.908807	3.797646	1.775374
2	6	0	-2.025133	3.604899	2.751474
3	6	0	-0.789921	4.442709	2.973011
4	6	0	0.509340	3.689897	2.602385
5	6	0	0.548547	2.437190	1.991214
6	6	0	-0.453979	1.389405	1.552378
7	6	0	0.062622	0.771825	0.215847
8	6	0	1.468088	1.318716	0.105975
9	6	0	2.947855	3.913624	2.234122
10	6	0	2.263433	1.555642	-1.002115
11	6	0	3.413959	2.449162	-0.935501
12	6	0	3.807895	3.313875	-2.064436
13	6	0	2.877335	3.488680	-3.197722
14	6	0	1.638744	2.719549	-3.254606
15	6	0	0.452275	3.343731	-3.632747

16	6	0	-0.908455	3.166570	-2.956739
17	6	0	-1.421180	4.614092	-2.791556
18	6	0	-2.019429	5.194270	-1.668266
19	6	0	-2.780501	4.388270	-0.662564
20	6	0	-2.857614	4.944426	0.802339
21	6	0	-1.693814	5.872633	1.066164
22	6	0	-0.783713	5.683314	2.084254
23	6	0	0.371465	6.543884	2.194720
24	6	0	1.657494	5.966918	2.578855
25	6	0	1.729568	4.498313	2.639247
26	6	0	1.740241	2.045658	1.297935
27	6	0	2.823065	6.708194	2.262240
28	6	0	4.046567	6.053412	1.835318
29	6	0	4.078177	4.676051	1.779009
30	6	0	4.621485	4.006439	0.608000
31	6	0	3.832164	2.836830	0.355956
32	6	0	2.921931	2.712090	1.458617
33	6	0	5.107986	4.740853	-0.451563
34	6	0	4.713314	4.378150	-1.802124
35	6	0	4.697789	5.616504	-2.556497
36	6	0	3.811029	5.776148	-3.604201
37	6	0	2.908046	4.700747	-3.919497
38	6	0	1.693814	5.301822	-4.421695
39	6	0	0.496592	4.668242	-4.148353
40	6	0	-0.633898	5.425265	-3.659764
41	6	0	-0.521229	6.797807	-3.494223
42	6	0	-1.058514	7.384863	-2.296299
43	6	0	-1.699059	6.576275	-1.337141
44	6	0	-1.476520	6.898407	0.073725
45	6	0	-0.594379	7.926859	0.400983
46	6	0	0.340411	7.752424	1.490021
47	6	0	1.536464	8.485102	1.163636
48	6	0	2.763560	7.965337	1.540510
49	6	0	3.884753	8.040822	0.614650
50	6	0	4.663637	6.833562	0.780305
51	6	0	5.188181	6.185026	-0.346105
52	6	0	4.963476	6.732900	-1.662870
53	6	0	4.269057	7.935696	-1.819067
54	6	0	3.332713	8.097777	-2.919256
55	6	0	3.129400	7.044166	-3.813746
56	6	0	1.802905	6.743553	-4.300900
57	6	0	0.707398	7.486339	-3.848625
58	6	0	0.907356	8.548588	-2.886868
59	6	0	-0.192197	8.488278	-1.938830
60	6	0	0.039686	8.754796	-0.598287
61	6	0	1.363017	9.106740	-0.135358
62	6	0	2.427173	9.161037	-1.036465
63	6	0	3.713877	8.602860	-0.654102
64	6	0	2.196756	8.864080	-2.439574
65	6	0	-4.154342	5.777550	0.915279
66	6	0	-4.703356	6.068357	2.169817
67	6	0	-5.844873	6.863176	2.228095
68	6	0	-6.398468	7.338995	1.038810
69	6	0	-5.775098	6.996441	-0.160076
70	6	0	-0.777081	4.823514	4.466594
71	6	0	-1.455612	5.958099	4.928627
72	6	0	-1.468088	6.217771	6.296337
73	6	0	-0.810829	5.336737	7.155410
74	6	0	-0.174676	4.227813	6.598114
75	6	0	2.908807	-3.797646	1.775374
76	6	0	2.025133	-3.604899	2.751474
77	6	0	0.789921	-4.442709	2.973011
78	6	0	-0.509340	-3.689897	2.602385
79	6	0	-0.548547	-2.437190	1.991214
80	6	0	-0.062622	-0.771825	0.215847
81	6	0	0.453979	-1.389405	1.552378
82	6	0	-1.468088	-1.318716	0.105975
83	6	0	-2.263433	-1.555642	-1.002115
84	6	0	-3.413959	-2.449162	-0.935501
85	6	0	-3.807895	-3.313875	-2.064436
86	6	0	-2.877335	-3.488680	-3.197722
87	6	0	-1.638744	-2.719549	-3.254606
88	6	0	-0.452275	-3.343731	-3.632747
89	6	0	0.908455	-3.166570	-2.956739
90	6	0	1.421180	-4.614092	-2.791556
91	6	0	2.019429	-5.194270	-1.668266
92	6	0	2.780501	-4.388270	-0.662564
93	6	0	2.857614	-4.944426	0.802339
94	6	0	1.693814	-5.872633	1.066164
95	6	0	0.783713	-5.683314	2.084254
96	6	0	-0.371465	-6.543884	2.194720
97	6	0	-1.657494	-5.966918	2.578855
98	6	0	-1.729568	-4.498313	2.639247
99	6	0	-2.947855	-3.913624	2.234122
100	6	0	-2.921931	-2.712090	1.458617
101	6	0	-1.740241	-2.045658	1.297935
102	6	0	-3.832164	-2.836830	0.355956
103	6	0	-4.621485	-4.006439	0.608000
104	6	0	-4.078177	-4.676051	1.779009
105	6	0	-4.046567	-6.053412	1.835318
106	6	0	-2.823065	-6.708194	2.262240
107	6	0	-2.763560	-7.965337	1.540510
108	6	0	-1.536464	-8.485102	1.163636
109	6	0	-0.340411	-7.752424	1.490021
110	6	0	0.594379	-7.926859	0.400983
111	6	0	1.476520	-6.898407	0.073725
112	6	0	1.699059	-6.576275	-1.337141
113	6	0	1.058514	-7.384863	-2.296299
114	6	0	0.521229	-6.797807	-3.494223
115	6	0	0.633898	-5.425265	-3.659764
116	6	0	-0.496592	-4.668242	-4.148353
117	6	0	-1.693814	-5.301822	-4.421695
118	6	0	-2.908046	-4.700747	-3.919497
119	6	0	-3.811029	-5.776148	-3.604201
120	6	0	-4.697789	-5.616504	-2.556497
121	6	0	-4.713314	-4.378150	-1.802124
122	6	0	-5.107986	-4.740853	-0.451563
123	6	0	-5.188181	-6.185026	-0.346105
124	6	0	-4.663637	-6.833562	0.780305
125	6	0	-3.884753	-8.040822	0.614650
126	6	0	-3.713877	-8.602860	-0.654102
127	6	0	-2.427173	-9.161037	-1.036465
128	6	0	-1.363017	-9.106740	-0.135358
129	6	0	-0.039686	-8.754796	-0.598287
130	6	0	0.192197	-8.488278	-1.938830
131	6	0	-0.907356	-8.548588	-2.886868
132	6	0	-0.707398	-7.486339	-3.848625
133	6	0	-1.802905	-6.743553	-4.300900
134	6	0	-3.129400	-7.044166	-3.813746
135	6	0	-3.332713	-8.097777	-2.919256
136	6	0	-4.269057	-7.935696	-1.819067
137	6	0	-4.963476	-6.732900	-1.662870
138	6	0	-2.196756	-8.864080	-2.439574
139	6	0	4.154342	-5.777550	0.915279
140	6	0	4.703356	-6.068357	2.169817
141	6	0	5.844873	-6.863176	2.228095
142	6	0	6.398468	-7.338995	1.038810
143	6	0	5.775098	-6.996441	-0.160076
144	6	0	0.777081	-4.823514	4.466594
145	6	0	1.455612	-5.958099	4.928627

146	6	0	1.468088	-6.217771	6.296337
147	6	0	0.810829	-5.336737	7.155410
148	6	0	0.174676	-4.227813	6.598114
149	16	0	-1.619739	-1.070499	-2.597089
150	16	0	1.619739	1.070499	-2.597089
151	8	0	3.337011	-3.349105	-0.947271
152	8	0	1.415576	-0.947820	2.143973
153	8	0	-1.415576	0.947820	2.143973
154	8	0	1.381228	-2.147234	-2.509912
155	8	0	-3.337011	3.349105	-0.947271
156	8	0	-1.381228	2.147234	-2.509912
157	7	0	0.154316	-3.968342	5.284997
158	7	0	4.675563	-6.235555	-0.228084
159	7	0	-4.675563	6.235555	-0.228084
160	7	0	-0.154316	3.968342	5.284997
161	1	0	-3.737921	3.110865	1.645502
162	1	0	-2.129058	2.757444	3.416805
163	1	0	-0.561578	1.159396	-0.599004
164	1	0	-4.242320	5.672263	3.068020
165	1	0	-6.296124	7.105888	3.185741
166	1	0	-7.289151	7.958560	1.037629
167	1	0	-6.171781	7.345551	-1.111017
168	1	0	-1.952374	6.619759	4.227008
169	1	0	-1.979826	7.093632	6.684445
170	1	0	-0.791472	5.500989	8.227776
171	1	0	0.344716	3.512036	7.232173
172	1	0	3.737921	-3.110865	1.645502
173	1	0	2.129058	-2.757444	3.416805
174	1	0	0.561578	-1.159396	-0.599004
175	1	0	4.242320	-5.672263	3.068020
176	1	0	6.296124	-7.105888	3.185741
177	1	0	7.289151	-7.958560	1.037629
178	1	0	6.171781	-7.345551	-1.111017
179	1	0	1.952374	-6.619759	4.227008
180	1	0	1.979826	-7.093632	6.684445
181	1	0	0.791472	-5.500989	8.227776
182	1	0	-0.344716	-3.512036	7.232173

The total electronic energy was calculated to be –7119.7334592 Hartree.

Supplementary Table 16. Optimized structure of **6'** (B3LYP-D3/6-31G(d,p))

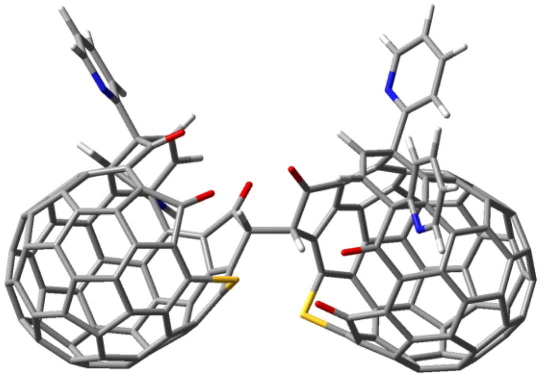

Standard orientation:

Center Number	Atomic Number	Atomic Type	Coordinates (Angstroms) X	Y	Z
1	6	0	-3.593372	3.476076	-1.503909
2	6	0	-3.420259	2.679120	-2.555411
3	6	0	-4.308390	1.510701	-2.906673
4	6	0	-3.617334	0.152825	-2.643845
5	6	0	-2.378978	0.012084	-2.020648
6	6	0	-1.292617	0.924812	-1.483865
7	6	0	-0.736019	0.276647	-0.182862
8	6	0	-1.355654	-1.098857	-0.186296
9	6	0	-3.946518	-2.298097	-2.486913
10	6	0	-1.651080	-1.969878	0.849287
11	6	0	-2.580233	-3.075613	0.665360
12	6	0	-3.482631	-3.531100	1.740349
13	6	0	-3.644737	-2.694479	2.945985
14	6	0	-2.829599	-1.496094	3.125184
15	6	0	-3.417033	-0.325956	3.597552
16	6	0	-3.166229	1.085809	3.057524
17	6	0	-4.593187	1.663931	2.899493
18	6	0	-5.121424	2.374438	1.817582
19	6	0	-4.262782	3.187908	0.900708
20	6	0	-4.770470	3.399387	-0.569693
21	6	0	-5.739536	2.303034	-0.951280
22	6	0	-5.565700	1.478385	-2.042510
23	6	0	-6.469967	0.374674	-2.269996
24	6	0	-5.938086	-0.897073	-2.753971
25	6	0	-4.473203	-1.026139	-2.795852
26	6	0	-2.053825	-1.248404	-1.416719
27	6	0	-6.731830	-2.053310	-2.552038
28	6	0	-6.136414	-3.334948	-2.219532
29	6	0	-4.762677	-3.429510	-2.141649
30	6	0	-4.141622	-4.094325	-1.006757
31	6	0	-2.948909	-3.374965	-0.665490
32	6	0	-2.762658	-2.385831	-1.687526
33	6	0	-4.918710	-4.635321	-0.005866
34	6	0	-4.572613	-4.369578	1.380864
35	6	0	-5.825979	-4.370285	2.109545
36	6	0	-5.974817	-3.569354	3.225583
37	6	0	-4.873237	-2.740113	3.639214
38	6	0	-5.439501	-1.551870	4.234333
39	6	0	-4.753852	-0.361950	4.079589
40	6	0	-5.455199	0.836447	3.676277
41	6	0	-6.826415	0.792898	3.471357
42	6	0	-7.362543	1.453742	2.311801
43	6	0	-6.504800	2.137212	1.428174
44	6	0	-6.798215	2.046774	-0.003255
45	6	0	-7.854222	1.241391	-0.426985
46	6	0	-7.692016	0.396385	-1.588777
47	6	0	-8.479459	-0.791435	-1.381214
48	6	0	-8.001927	-2.002838	-1.852722
49	6	0	-8.143100	-3.194521	-1.028175
50	6	0	-6.964027	-4.005902	-1.236257
51	6	0	-6.362025	-4.648959	-0.145800
52	6	0	-6.930935	-4.514561	1.174505
53	6	0	-8.108968	-3.787397	1.365381
54	6	0	-8.259968	-2.942052	2.538377
55	6	0	-7.220684	-2.858573	3.468179
56	6	0	-6.880906	-1.591992	4.074626
57	6	0	-7.569892	-0.432702	3.703405

58	6	0	-8.615389	-0.506546	2.705842
59	6	0	-8.489198	0.666454	1.857272
60	6	0	-8.732052	0.560680	0.496490
61	6	0	-9.124501	-0.703065	-0.085274
62	6	0	-9.242569	-1.836980	0.719584
63	6	0	-8.726969	-3.108742	0.239173
64	6	0	-8.970352	-1.739219	2.142950
65	6	0	-5.546357	4.736373	-0.603458
66	6	0	-5.751940	5.409232	-1.813769
67	6	0	-6.498515	6.584461	-1.803108
68	6	0	-7.012710	7.047860	-0.591614
69	6	0	-6.755510	6.303828	0.558972
70	6	0	-4.657430	1.650535	-4.400973
71	6	0	-5.749532	2.421517	-4.818296
72	6	0	-5.978953	2.570350	-6.183322
73	6	0	-5.111165	1.951789	-7.083802
74	6	0	-4.045756	1.214472	-6.568252
75	16	0	-1.166350	-1.486264	2.497564
76	8	0	-0.783463	1.899504	-1.994879
77	8	0	-3.221618	3.692229	1.261367
78	8	0	-2.116244	1.557045	2.695407
79	7	0	-6.042127	5.171153	0.560195
80	7	0	-3.815183	1.062872	-5.258151
81	6	0	3.237449	2.232635	2.975003
82	6	0	3.235950	3.111046	1.975147
83	6	0	4.281443	3.182372	0.889121
84	6	0	3.751754	2.661840	-0.467308
85	6	0	2.510888	2.055473	-0.640169
86	6	0	1.295628	1.764729	0.217629
87	6	0	0.804968	0.329387	-0.153882
88	6	0	1.551637	0.033688	-1.442664
89	6	0	4.372746	1.970688	-2.761866
90	6	0	1.883069	-1.160797	-2.061023
91	6	0	2.946868	-1.240797	-3.057517
92	6	0	3.837823	-2.413628	-3.170187
93	6	0	3.830935	-3.434140	-2.102105
94	6	0	2.863245	-3.350992	-1.015238
95	6	0	3.267982	-3.583358	0.295559
96	6	0	2.866971	-2.750488	1.514006
97	6	0	4.209614	-2.499757	2.234912
98	6	0	4.696342	-1.301707	2.769366
99	6	0	3.786256	-0.207872	3.237765
100	6	0	4.351404	1.256000	3.241003
101	6	0	5.477714	1.379426	2.238849
102	6	0	5.477340	2.286342	1.200182
103	6	0	6.526548	2.253037	0.206945
104	6	0	6.187334	2.476182	-1.197030
105	6	0	4.755038	2.531508	-1.525636
106	6	0	2.317203	1.190487	-1.765130
107	6	0	7.106775	2.012504	-2.170363
108	6	0	6.657568	1.434763	-3.424304
109	6	0	5.303462	1.374131	-3.679906
110	6	0	4.711073	0.141376	-4.174248
111	6	0	3.424042	-0.012162	-3.561300
112	6	0	3.170187	1.196953	-2.832652
113	6	0	5.494161	-0.969699	-4.397264
114	6	0	5.043510	-2.261562	-3.908144
115	6	0	6.247268	-3.008093	-3.595497
116	6	0	6.236558	-3.941251	-2.576598
117	6	0	5.018212	-4.151082	-1.839811
118	6	0	5.396286	-4.500948	-0.489400
119	6	0	4.572653	-4.090446	0.541611
120	6	0	5.131286	-3.456403	1.714950
121	6	0	6.504801	-3.295840	1.817109
122	6	0	7.009945	-2.036211	2.292828
123	6	0	6.118737	-1.007325	2.655475
124	6	0	6.502568	0.366086	2.323047
125	6	0	7.677319	0.584396	1.604637
126	6	0	7.693282	1.552255	0.530628
127	6	0	8.612239	1.079635	-0.472584
128	6	0	8.318837	1.302076	-1.807929
129	6	0	8.562351	0.242914	-2.776712
130	6	0	7.507276	0.309701	-3.763762
131	6	0	6.933649	-0.873886	-4.248192
132	6	0	7.409558	-2.152274	-3.775339
133	6	0	8.474937	-2.220112	-2.873223
134	6	0	8.455477	-3.196683	-1.796344
135	6	0	7.367111	-4.063005	-1.669160
136	6	0	6.837159	-4.386048	-0.364585
137	6	0	7.387646	-3.797282	0.778258
138	6	0	8.485170	-2.863450	0.647904
139	6	0	8.253227	-1.786216	1.595075
140	6	0	8.581782	-0.484380	1.250760
141	6	0	9.165901	-0.188318	-0.037833
142	6	0	9.384462	-1.214083	-0.958292
143	6	0	9.060675	-0.994561	-2.358470
144	6	0	9.025328	-2.578402	-0.612715
145	6	0	4.943339	1.518267	4.645068
146	6	0	5.136456	2.827212	5.102215
147	6	0	5.717474	3.015738	6.353415
148	6	0	6.083888	1.898078	7.104163
149	6	0	5.851319	0.635780	6.560159
150	6	0	4.709127	4.657710	0.772794
151	6	0	5.669835	5.194737	1.639366
152	6	0	5.973627	6.549475	1.541817
153	6	0	5.307169	7.322120	0.589793
154	6	0	4.360471	6.695507	-0.219469
155	16	0	1.255347	-2.673922	-1.349617
156	8	0	0.702759	2.487295	0.982291
157	8	0	2.656004	-0.423803	3.620411
158	8	0	1.780096	-2.275697	1.747296
159	7	0	5.296350	0.442552	5.357309
160	7	0	4.061009	5.392879	-0.136341
161	1	0	-2.868675	4.251126	-1.279865
162	1	0	-2.551453	2.800795	-3.189530
163	1	0	-1.080879	0.872170	0.674697
164	1	0	-5.329326	5.015935	-2.731761
165	1	0	-6.674732	7.130516	-2.725305
166	1	0	-7.597202	7.960318	-0.536884
167	1	0	-7.137210	6.626884	1.525185
168	1	0	-6.401979	2.883658	-4.085147
169	1	0	-6.821681	3.156590	-6.537937
170	1	0	-5.253104	2.037480	-8.156091
171	1	0	-3.342223	0.720033	-7.235145
172	1	0	2.407619	2.199330	3.672353
173	1	0	2.401139	3.789600	1.854524
174	1	0	1.148111	-0.336893	0.648018
175	1	0	4.829460	3.666841	4.488470
176	1	0	5.879351	4.018803	6.737134
177	1	0	6.536360	1.998944	8.085108
178	1	0	6.120080	-0.263449	7.110356
179	1	0	6.165628	4.557835	2.364531
180	1	0	6.718083	6.994534	2.195510
181	1	0	5.512958	8.381441	0.476576
182	1	0	3.814391	7.261552	-0.971391

The total electronic energy was calculated to be –7119.7325875 Hartree.

Radical Anions

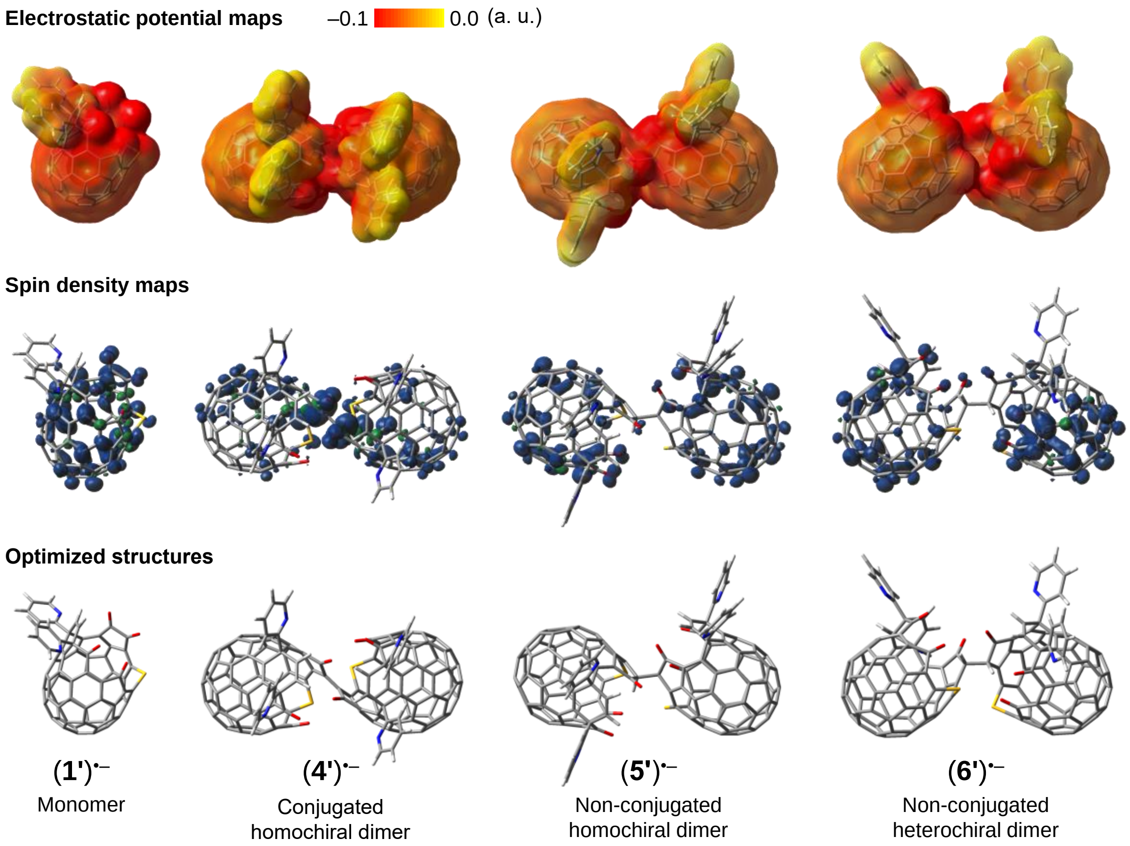

Supplementary Figure 33. Electrostatic potential maps, spin density, and structures of (**1'**)$^{\bullet-}$, (**4'**)$^{\bullet-}$, (**5'**)$^{\bullet-}$, and (**6'**)$^{\bullet-}$ (UB3LYP-D3/6-31G(d,p)).

Supplementary Table 17. Optimized structure of (**1'**)$^{\bullet-}$ (UB3LYP-D3/6-31G(d,p))

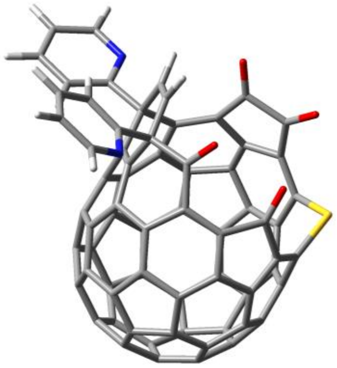

Standard orientation:

Center Number	Atomic Number	Atomic Type	X	Y	Z
1	6	0	3.765439	0.216337	1.518340
2	6	0	3.790990	-0.995079	0.967830
3	6	0	3.001531	-1.405058	-0.252195
4	6	0	1.839905	-2.362579	0.109679
5	6	0	1.485213	-2.695248	1.429129
6	6	0	2.003191	-2.474925	2.822146
7	6	0	0.734227	-2.367988	3.748793
8	6	0	-0.425510	-2.671384	2.876922
9	6	0	-1.768213	-2.307560	2.896068
10	6	0	-2.876444	0.063603	3.074927
11	6	0	-2.273528	1.317786	3.083503
12	6	0	-0.810169	1.638435	3.415390
13	6	0	-0.394193	2.597450	2.272352
14	6	0	0.774110	2.570157	1.500875
15	6	0	2.049310	1.991814	2.001109
16	6	0	3.038585	1.396840	0.932806
17	16	0	-2.323810	-1.209924	4.191764
18	7	0	3.729621	3.762251	0.846876
19	8	0	3.132636	-2.455636	3.273456
20	8	0	2.360511	1.983999	3.175706
21	7	0	4.235178	-3.389894	-0.983225
22	6	0	2.303031	1.046332	-0.342273
23	6	0	1.502255	-0.493574	-2.070796
24	8	0	0.776246	-2.114734	4.934411
25	6	0	1.440046	2.078994	-0.861589
26	6	0	0.911281	-2.649528	-0.979659
27	8	0	-0.127133	1.150885	4.281671
28	6	0	-0.278190	-1.883550	-3.044625
29	6	0	5.144780	-4.005052	-1.747372
30	6	0	-1.430873	-2.687927	-2.717131
31	6	0	3.999461	-2.090322	-1.205506
32	6	0	0.847595	-1.786681	-2.175032
33	6	0	0.999164	0.605594	-2.782220
34	6	0	2.329351	-0.207908	-0.914922
35	6	0	-1.421016	-3.407587	-1.525488
36	6	0	-0.142668	0.492392	-3.656108
37	6	0	-0.670039	-3.571656	0.645208
38	6	0	-0.776058	-0.733117	-3.777193
39	6	0	0.956296	1.908768	-2.166271
40	6	0	0.681698	2.894160	0.076860
41	6	0	-0.240763	-3.425021	-0.714670
42	6	0	5.262094	2.155231	-0.070101
43	6	0	4.063189	2.496224	0.572676
44	6	0	-3.679028	-2.596315	-0.956547
45	6	0	-0.477862	3.536084	-0.406159
46	6	0	-0.217618	2.588858	-2.648463
47	6	0	6.139126	3.171307	-0.433932
48	6	0	0.140410	-3.144110	1.657177
49	6	0	-2.230698	-0.782045	-3.812827
50	6	0	5.863478	-3.370341	-2.759966
51	6	0	-4.274051	-1.746170	0.044451
52	6	0	-2.979679	0.391608	-3.691571
53	6	0	-2.082402	-3.343106	0.714883
54	6	0	5.797599	4.494224	-0.146081
55	6	0	4.680370	-1.361319	-2.190968
56	6	0	-2.621645	-2.519982	1.733275
57	6	0	4.582957	4.731458	0.494140
58	6	0	-0.912722	1.718326	-3.573162
59	6	0	-4.821626	-0.619004	-0.674971
60	6	0	-4.359681	0.778037	1.263383
61	6	0	-2.564118	-3.350293	-0.636219
62	6	0	-1.644423	3.651373	0.423801
63	6	0	-2.632618	-1.984231	-3.113120
64	6	0	-4.139816	0.426523	-2.815907
65	6	0	-3.723682	-1.618651	1.368658
66	6	0	5.623078	-2.013724	-2.979643
67	6	0	-1.600607	3.087753	1.699248
68	6	0	-3.794403	-0.295168	1.993742
69	6	0	-4.487259	-0.724719	-2.093127
70	6	0	-3.748284	-1.945802	-2.261425
71	6	0	-2.719379	2.312639	2.171050
72	6	0	-2.307427	1.675979	-3.582452
73	6	0	-3.062455	2.506229	-2.658736
74	6	0	-0.935351	3.396576	-1.774536
75	6	0	-4.584065	1.832199	-0.837537
76	6	0	-2.384520	3.368388	-1.782716
77	6	0	-2.822965	3.503747	-0.406453
78	6	0	-3.827591	2.110422	1.362880
79	6	0	-4.870941	0.629158	-0.078617
80	6	0	-3.901739	2.739119	0.053483
81	6	0	-4.190260	1.729052	-2.185358
82	1	0	4.295108	0.407160	2.445349
83	1	0	4.338351	-1.795088	1.449106
84	1	0	5.305665	-5.061444	-1.537199
85	1	0	5.486013	1.113374	-0.270939
86	1	0	7.074772	2.935944	-0.933914
87	1	0	6.585854	-3.922203	-3.353366
88	1	0	6.452044	5.319216	-0.409591
89	1	0	4.459819	-0.309200	-2.333331
90	1	0	4.274656	5.746830	0.737186
91	1	0	6.157908	-1.473132	-3.755882

The total electronic energy was calculated to be –3634.5348713 Hartree.

Supplementary Table 18. Optimized structure of $(\mathbf{4'})^{\bullet-}$ (UB3LYP-D3/6-31G(d,p))

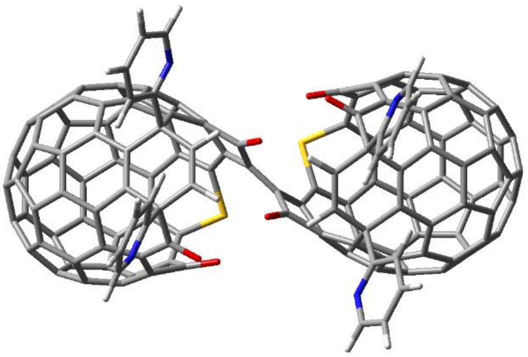

Standard orientation:

Center Number	Atomic Number	Atomic Type	X	Y	Z
1	6	0	-2.590422	3.279857	0.174616
2	6	0	-2.559286	3.063805	-1.138139
3	6	0	-3.674498	2.435622	-1.938893
4	6	0	-3.289056	1.013157	-2.410118
5	6	0	-2.116140	0.367576	-2.037396
6	6	0	-0.842683	0.692797	-1.264437
7	6	0	-0.499864	-0.555810	-0.480619
8	6	0	-1.360203	-1.609678	-0.961756
9	6	0	-4.130166	-1.119366	-3.344457
10	6	0	-1.886389	-2.789894	-0.390271
11	6	0	-3.028784	-3.473722	-0.967304
12	6	0	-4.045082	-4.181117	-0.153613
13	6	0	-4.083587	-3.969821	1.302887
14	6	0	-3.057643	-3.162533	1.953319
15	6	0	-3.405637	-2.258646	2.952370
16	6	0	-2.857522	-0.835368	3.122496
17	6	0	-4.137703	0.004359	3.338036
18	6	0	-4.478332	1.214047	2.731423
19	6	0	-3.456666	2.204512	2.273231
20	6	0	-3.815046	3.092111	1.029235
21	6	0	-4.977929	2.502916	0.255536
22	6	0	-4.938506	2.250414	-1.100963
23	6	0	-6.044785	1.581563	-1.751153
24	6	0	-5.775958	0.605133	-2.802899
25	6	0	-4.373722	0.229756	-3.009486
26	6	0	-2.087959	-1.062498	-2.074748
27	6	0	-6.799585	-0.339800	-3.093127
28	6	0	-6.492116	-1.716182	-3.419054
29	6	0	-5.167950	-2.101871	-3.480643
30	6	0	-4.723728	-3.315666	-2.810249
31	6	0	-3.426382	-3.076757	-2.271292
32	6	0	-3.010238	-1.787724	-2.757192
33	6	0	-5.628455	-4.098820	-2.113056
34	6	0	-5.274215	-4.544382	-0.782378
35	6	0	-6.523386	-4.637517	-0.048002
36	6	0	-6.544246	-4.425646	1.317282
37	6	0	-5.313363	-4.103109	1.987581
38	6	0	-5.647721	-3.236909	3.092323
39	6	0	-4.735241	-2.263702	3.459224
40	6	0	-5.172573	-0.908739	3.697014
41	6	0	-6.518335	-0.588367	3.577517
42	6	0	-6.871280	0.616509	2.877097
43	6	0	-5.862743	1.448293	2.348975
44	6	0	-6.105515	2.068317	1.048010
45	6	0	-7.287151	1.767267	0.368065
46	6	0	-7.259839	1.530996	-1.055430
47	6	0	-8.283626	0.562428	-1.359397
48	6	0	-8.049044	-0.368493	-2.357835
49	6	0	-8.460911	-1.752020	-2.156681
50	6	0	-7.469046	-2.589492	-2.786499
51	6	0	-7.037953	-3.760176	-2.145136
52	6	0	-7.609213	-4.131830	-0.871356
53	6	0	-8.625673	-3.360760	-0.296208
54	6	0	-8.638399	-3.131675	1.139350
55	6	0	-7.631561	-3.682510	1.936540
56	6	0	-7.063745	-2.924733	3.028193
57	6	0	-7.497201	-1.616370	3.269957
58	6	0	-8.503711	-1.024121	2.415033
59	6	0	-8.113392	0.357112	2.182745
60	6	0	-8.319559	0.929771	0.936577
61	6	0	-8.943351	0.176447	-0.127404
62	6	0	-9.319218	-1.153469	0.083136
63	6	0	-9.059918	-2.140287	-0.950278
64	6	0	-9.079642	-1.769104	1.377644
65	6	0	-4.287087	4.462956	1.570672
66	6	0	-4.161315	5.622995	0.795429
67	6	0	-4.658864	6.821422	1.299433
68	6	0	-5.262756	6.828352	2.557054
69	6	0	-5.341436	5.621657	3.251110
70	6	0	-3.949491	3.361509	-3.138467
71	6	0	-4.769093	4.490406	-2.992212
72	6	0	-4.940279	5.340652	-4.079433
73	6	0	-4.286031	5.043748	-5.276391
74	6	0	-3.486250	3.902861	-5.319324
75	6	0	2.590416	3.279842	-0.174610
76	6	0	2.559306	3.063801	1.138148
77	6	0	3.674541	2.435637	1.938886
78	6	0	3.289112	1.013178	2.410138
79	6	0	2.116189	0.367594	2.037447
80	6	0	0.842723	0.692807	1.264502
81	6	0	0.499888	-0.555808	0.480705
82	6	0	1.360225	-1.609674	0.961849
83	6	0	1.886395	-2.789901	0.390373
84	6	0	3.028802	-3.473721	0.967390
85	6	0	4.045082	-4.181126	0.153684
86	6	0	4.083553	-3.969848	-1.302820
87	6	0	3.057593	-3.162568	-1.953237
88	6	0	3.405565	-2.258682	-2.952296
89	6	0	2.857458	-0.835398	-3.122394
90	6	0	4.137629	0.004323	-3.338005
91	6	0	4.478278	1.214026	-2.731433
92	6	0	3.456633	2.204514	-2.273245
93	6	0	3.815028	3.092106	-1.029248
94	6	0	4.977927	2.502914	-0.255570
95	6	0	4.938532	2.250422	1.100932
96	6	0	6.044824	1.581578	1.751106
97	6	0	5.776021	0.605161	2.802870
98	6	0	4.373790	0.229786	3.009494
99	6	0	4.130240	-1.119332	3.344486
100	6	0	3.010298	-1.787698	2.757254
101	6	0	2.088005	-1.062479	2.074819
102	6	0	3.426431	-3.076737	2.271363
103	6	0	4.723790	-3.315639	2.810293
104	6	0	5.168027	-2.101836	3.480661

105	6	0	6.492192	-1.716147	3.419038
106	6	0	6.799654	-0.339769	3.093087
107	6	0	8.049096	-0.368472	2.357768
108	6	0	8.283657	0.562438	1.359314
109	6	0	7.259864	1.531003	1.055358
110	6	0	7.287146	1.767260	-0.368140
111	6	0	6.105497	2.068307	-1.048064
112	6	0	5.862698	1.448271	-2.349019
113	6	0	6.871219	0.616474	-2.877150
114	6	0	6.518252	-0.588412	-3.577542
115	6	0	5.172487	-0.908783	-3.696997
116	6	0	4.735158	-2.263742	-3.459181
117	6	0	5.647646	-3.236948	-3.092297
118	6	0	5.313314	-4.103139	-1.987540
119	6	0	6.544213	-4.425668	-1.317266
120	6	0	6.523385	-4.637524	0.048021
121	6	0	5.274230	-4.544382	0.782425
122	6	0	5.628501	-4.098802	2.113090
123	6	0	7.037999	-3.760157	2.145133
124	6	0	7.469108	-2.589465	2.786472
125	6	0	8.460958	-1.752001	2.156622
126	6	0	9.059938	-2.140283	0.950209
127	6	0	9.319216	-1.153477	-0.083221
128	6	0	8.943354	0.176442	0.127311
129	6	0	8.319541	0.929755	-0.936665
130	6	0	8.113345	0.357081	-2.182821
131	6	0	8.503656	-1.024155	-2.415102
132	6	0	7.497124	-1.616413	-3.269995
133	6	0	7.063672	-2.924773	-3.028205
134	6	0	7.631514	-3.682539	-1.936557
135	6	0	8.638370	-3.131695	-1.139397
136	6	0	8.625678	-3.360763	0.296164
137	6	0	7.609230	-4.131827	0.871344
138	6	0	9.079609	-1.769127	-1.377717
139	6	0	4.287051	4.462956	-1.570686
140	6	0	4.161263	5.622992	-0.795441
141	6	0	4.658798	6.821426	-1.299441
142	6	0	5.262694	6.828366	-2.557059
143	6	0	5.341393	5.621673	-3.251116
144	6	0	3.949554	3.361542	3.138442
145	6	0	4.769147	4.490441	2.992153
146	6	0	4.940351	5.340704	4.079359
147	6	0	4.286131	5.043812	5.276336
148	6	0	3.486357	3.902922	5.319301
149	16	0	1.415121	-3.171934	-1.291296
150	16	0	-1.415155	-3.171903	1.291416
151	8	0	2.411291	2.371863	-2.861545
152	8	0	0.135434	1.675612	1.376307
153	8	0	-0.135351	1.675564	-1.376301
154	8	0	1.721781	-0.455694	-2.977380
155	8	0	-2.411405	2.371974	2.861643
156	8	0	-1.721872	-0.455648	2.977319
157	7	0	3.316096	3.075611	4.280623
158	7	0	4.870393	4.463675	-2.775790
159	7	0	-4.870424	4.463665	2.775780
160	7	0	-3.316007	3.075566	-4.280631
161	1	0	-1.703793	3.623168	0.695994
162	1	0	-1.645332	3.239608	-1.690209
163	1	0	-3.679752	5.573215	-0.174206
164	1	0	-4.574005	7.736192	0.719314
165	1	0	-5.661300	7.740179	2.990393
166	1	0	-5.803987	5.578548	4.235545
167	1	0	-5.259580	4.683343	-2.044075
168	1	0	-5.574710	6.218785	-3.995763
169	1	0	-4.390979	5.676672	-6.151956
170	1	0	-2.953922	3.635526	-6.230454
171	1	0	1.703775	3.623143	-0.695974
172	1	0	1.645360	3.239599	1.690233
173	1	0	3.679697	5.573204	0.174194
174	1	0	4.573925	7.736195	-0.719320
175	1	0	5.661228	7.740199	-2.990395
176	1	0	5.803948	5.578572	-4.235550
177	1	0	5.259612	4.683369	2.044003
178	1	0	5.574776	6.218839	3.995664
179	1	0	4.391094	5.676749	6.151890
180	1	0	2.954050	3.635596	6.230446

The total electronic energy was calculated to be –7118.5849014 Hartree.

Supplementary Table 19. Optimized structure of (**5'**)$^{\bullet-}$ (UB3LYP-D3/6-31G(d,p))

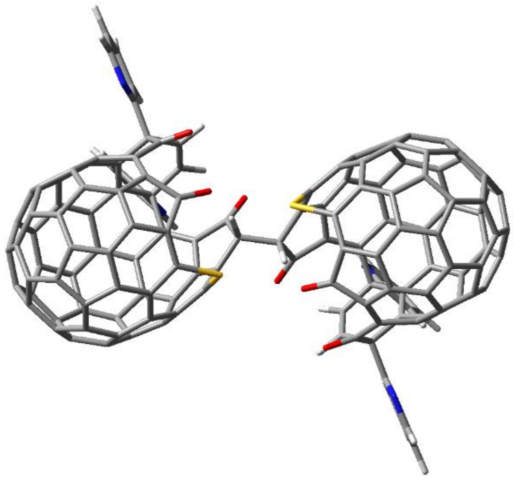

Standard orientation:

Center Number	Atomic Number	Atomic Type	Coordinates (Angstroms) X	Y	Z
1	6	0	-3.288692	3.467170	1.788431
2	6	0	-2.380202	3.369305	2.755889
3	6	0	-1.245779	4.340718	2.973881
4	6	0	0.126118	3.734391	2.593976
5	6	0	0.294962	2.488405	1.982535
6	6	0	-0.587169	1.333467	1.557591
7	6	0	-0.022381	0.774351	0.216948
8	6	0	1.311541	1.474232	0.088050
9	6	0	2.520788	4.224073	2.196770
10	6	0	2.062715	1.793623	-1.029107
11	6	0	3.108862	2.810410	-0.979576
12	6	0	3.391347	3.706136	-2.111918
13	6	0	2.430960	3.778400	-3.230440
14	6	0	1.282433	2.878604	-3.273127

15	6	0	0.027143	3.370928	-3.627211
16	6	0	-1.298632	3.058991	-2.926825
17	6	0	-1.966482	4.432532	-2.779315
18	6	0	-2.622025	4.954240	-1.646600
19	6	0	-3.297526	4.089313	-0.646246
20	6	0	-3.385775	4.624074	0.830918
21	6	0	-2.322180	5.669136	1.082170
22	6	0	-1.384672	5.576247	2.089993
23	6	0	-0.328283	6.557212	2.188461
24	6	0	1.016849	6.124079	2.558549
25	6	0	1.248094	4.670319	2.618071
26	6	0	1.513217	2.232435	1.274909
27	6	0	2.091423	6.987396	2.229121
28	6	0	3.375379	6.471495	1.786586
29	6	0	3.555187	5.103502	1.730548
30	6	0	4.153257	4.495655	0.550744
31	6	0	3.496295	3.245700	0.308696
32	6	0	2.616397	3.026440	1.419631
33	6	0	4.542523	5.278652	-0.514772
34	6	0	4.177330	4.870806	-1.861066
35	6	0	4.018936	6.094650	-2.613608
36	6	0	3.102976	6.156352	-3.649735
37	6	0	2.319791	4.986572	-3.951556
38	6	0	1.041808	5.448758	-4.439951
39	6	0	-0.075135	4.691260	-4.147122
40	6	0	-1.278402	5.321348	-3.649564
41	6	0	-1.311541	6.702069	-3.492653
42	6	0	-1.890324	7.231518	-2.282987
43	6	0	-2.430960	6.358914	-1.316764
44	6	0	-2.231710	6.711800	0.090376
45	6	0	-1.460197	7.831272	0.407319
46	6	0	-0.498819	7.755707	1.485551
47	6	0	0.606957	8.613695	1.146718
48	6	0	1.887443	8.230439	1.509159
49	6	0	2.985226	8.425639	0.571528
50	6	0	3.892266	7.313808	0.728496
51	6	0	4.475282	6.724735	-0.409349
52	6	0	4.171456	7.238213	-1.720204
53	6	0	3.344350	8.356510	-1.871880
54	6	0	2.388663	8.419383	-2.962683
55	6	0	2.288447	7.341217	-3.858333
56	6	0	0.999368	6.897285	-4.324078
57	6	0	-0.168577	7.514336	-3.856991
58	6	0	-0.072893	8.594902	-2.895779
59	6	0	-1.147191	8.421234	-1.936832
60	6	0	-0.935082	8.725113	-0.594577
61	6	0	0.349460	9.210990	-0.151175
62	6	0	1.394038	9.377417	-1.064036
63	6	0	2.735083	8.962644	-0.697705
64	6	0	1.182388	9.049366	-2.466210
65	6	0	-4.755500	5.318233	1.005734
66	6	0	-5.280327	5.529128	2.288224
67	6	0	-6.489336	6.206143	2.413117
68	6	0	-7.137112	6.650466	1.259306
69	6	0	-6.533074	6.394368	0.029601
70	6	0	-1.276161	4.714629	4.468441
71	6	0	-2.049345	5.789435	4.928658
72	6	0	-2.098135	6.043010	6.296102
73	6	0	-1.381098	5.215812	7.161265
74	6	0	-0.651670	4.164442	6.607090
75	6	0	3.288692	-3.467170	1.788431
76	6	0	2.380202	-3.369305	2.755889
77	6	0	1.245779	-4.340718	2.973881
78	6	0	-0.126118	-3.734391	2.593976
79	6	0	-0.294962	-2.488405	1.982535
80	6	0	0.022381	-0.774351	0.216948
81	6	0	0.587169	-1.333467	1.557591
82	6	0	-1.311541	-1.474232	0.088050
83	6	0	-2.062715	-1.793623	-1.029107
84	6	0	-3.108862	-2.810410	-0.979576
85	6	0	-3.391347	-3.706136	-2.111918
86	6	0	-2.430960	-3.778400	-3.230440
87	6	0	-1.282433	-2.878604	-3.273127
88	6	0	-0.027143	-3.370928	-3.627211
89	6	0	1.298632	-3.058991	-2.926825
90	6	0	1.966482	-4.432532	-2.779315
91	6	0	2.622025	-4.954240	-1.646600
92	6	0	3.297526	-4.089313	-0.646246
93	6	0	3.385775	-4.624074	0.830918
94	6	0	2.322180	-5.669136	1.082170
95	6	0	1.384672	-5.576247	2.089993
96	6	0	0.328283	-6.557212	2.188461
97	6	0	-1.016849	-6.124079	2.558549
98	6	0	-1.248094	-4.670319	2.618071
99	6	0	-2.520788	-4.224073	2.196770
100	6	0	-2.616397	-3.026440	1.419631
101	6	0	-1.513217	-2.232435	1.274909
102	6	0	-3.496295	-3.245700	0.308696
103	6	0	-4.153257	-4.495655	0.550744
104	6	0	-3.555187	-5.103502	1.730548
105	6	0	-3.375379	-6.471495	1.786586
106	6	0	-2.091423	-6.987396	2.229121
107	6	0	-1.887443	-8.230439	1.509159
108	6	0	-0.606957	-8.613695	1.146718
109	6	0	0.498819	-7.755707	1.485551
110	6	0	1.460197	-7.831272	0.407319
111	6	0	2.231710	-6.711800	0.090376
112	6	0	2.430960	-6.358914	-1.316764
113	6	0	1.890324	-7.231518	-2.282987
114	6	0	1.311541	-6.702069	-3.492653
115	6	0	1.278402	-5.321348	-3.649564
116	6	0	0.075135	-4.691260	-4.147122
117	6	0	-1.041808	-5.448758	-4.439951
118	6	0	-2.319791	-4.986572	-3.951556
119	6	0	-3.102976	-6.156352	-3.649735
120	6	0	-4.018936	-6.094650	-2.613608
121	6	0	-4.177330	-4.870806	-1.861066
122	6	0	-4.542523	-5.278652	-0.514772
123	6	0	-4.475282	-6.724735	-0.409349
124	6	0	-3.892266	-7.313808	0.728496
125	6	0	-2.985226	-8.425639	0.571528
126	6	0	-2.735083	-8.962644	-0.697705
127	6	0	-1.394038	-9.377417	-1.064036
128	6	0	-0.349460	-9.210990	-0.151175
129	6	0	0.935082	-8.725113	-0.594577
130	6	0	1.147191	-8.421234	-1.936832
131	6	0	0.072893	-8.594902	-2.895779
132	6	0	0.168577	-7.514336	-3.856991
133	6	0	-0.999368	-6.897285	-4.324078
134	6	0	-2.288447	-7.341217	-3.858333
135	6	0	-2.388663	-8.419383	-2.962683
136	6	0	-3.344350	-8.356510	-1.871880
137	6	0	-4.171456	-7.238213	-1.720204
138	6	0	-1.182388	-9.049366	-2.466210
139	6	0	4.755500	-5.318233	1.005734
140	6	0	5.280327	-5.529128	2.288224
141	6	0	6.489336	-6.206143	2.413117
142	6	0	7.137112	-6.650466	1.259306
143	6	0	6.533074	-6.394368	0.029601
144	6	0	1.276161	-4.714629	4.468441

145	6	0	2.049345	-5.789435	4.928658
146	6	0	2.098135	-6.043010	6.296102
147	6	0	1.381098	-5.215812	7.161265
148	6	0	0.651670	-4.164442	6.607090
149	16	0	-1.462381	-1.236796	-2.617967
150	16	0	1.462381	1.236796	-2.617967
151	8	0	3.794235	-3.014253	-0.922224
152	8	0	1.495740	-0.793443	2.154951
153	8	0	-1.495740	0.793443	2.154951
154	8	0	1.645650	-2.000991	-2.445670
155	8	0	-3.794235	3.014253	-0.922224
156	8	0	-1.645650	2.000991	-2.445670
157	7	0	0.595729	-3.911174	5.294352
158	7	0	5.369400	-5.746586	-0.102724
159	7	0	-5.369400	5.746586	-0.102724
160	7	0	-0.595729	3.911174	5.294352
161	1	0	-4.028459	2.685809	1.652467
162	1	0	-2.374697	2.505580	3.408023
163	1	0	-0.694176	1.091170	-0.590783
164	1	0	-4.743760	5.162502	3.156390
165	1	0	-6.920022	6.383481	3.394712
166	1	0	-8.083221	7.180063	1.308487
167	1	0	-7.001514	6.722991	-0.896283
168	1	0	-2.589164	6.410284	4.221915
169	1	0	-2.683464	6.874039	6.679795
170	1	0	-1.386318	5.377557	8.234541
171	1	0	-0.082860	3.490441	7.245301
172	1	0	4.028459	-2.685809	1.652467
173	1	0	2.374697	-2.505580	3.408023
174	1	0	0.694176	-1.091170	-0.590783
175	1	0	4.743760	-5.162502	3.156390
176	1	0	6.920022	-6.383481	3.394712
177	1	0	8.083221	-7.180063	1.308487
178	1	0	7.001514	-6.722991	-0.896283
179	1	0	2.589164	-6.410284	4.221915
180	1	0	2.683464	-6.874039	6.679795
181	1	0	1.386318	-5.377557	8.234541
182	1	0	0.082860	-3.490441	7.245301

The total electronic energy was calculated to be –7119.8177619 Hartree.

Supplementary Table 20. Optimized structure of (**6'**)$^{\bullet-}$ (UB3LYP-D3/6-31G(d,p))

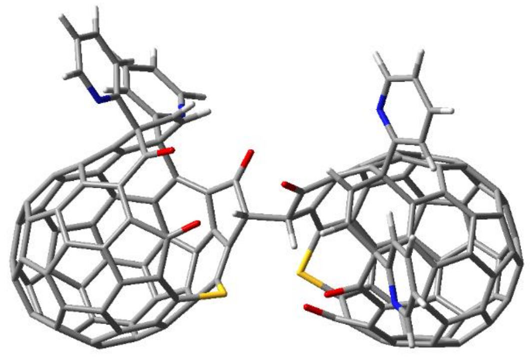

Standard orientation:

Center Number	Atomic Number	Atomic Type	Coordinates (Angstroms) X	Y	Z
1	6	0	-3.665602	3.506654	-1.440597
2	6	0	-3.449448	2.714846	-2.488066
3	6	0	-4.306516	1.530913	-2.865312
4	6	0	-3.589193	0.187228	-2.595206
5	6	0	-2.354480	0.074420	-1.949297
6	6	0	-1.290927	1.005350	-1.399100
7	6	0	-0.724074	0.359858	-0.100829
8	6	0	-1.340965	-1.015558	-0.098476
9	6	0	-3.867610	-2.270434	-2.443247
10	6	0	-1.633808	-1.891275	0.931640
11	6	0	-2.536839	-3.018096	0.734209
12	6	0	-3.442200	-3.493536	1.791231
13	6	0	-3.647509	-2.657175	2.988695
14	6	0	-2.861911	-1.440059	3.183173
15	6	0	-3.487815	-0.279856	3.634058
16	6	0	-3.270441	1.134737	3.085745
17	6	0	-4.702916	1.682971	2.926597
18	6	0	-5.235717	2.390490	1.836526
19	6	0	-4.398116	3.239925	0.947983
20	6	0	-4.869476	3.412475	-0.542648
21	6	0	-5.799974	2.288958	-0.942105
22	6	0	-5.581098	1.468537	-2.029353
23	6	0	-6.455072	0.343210	-2.274186
24	6	0	-5.884744	-0.914652	-2.748911
25	6	0	-4.414555	-1.007441	-2.763126
26	6	0	-2.019134	-1.177488	-1.338601
27	6	0	-6.655112	-2.089901	-2.562831
28	6	0	-6.038814	-3.359484	-2.219652
29	6	0	-4.662427	-3.419849	-2.115364
30	6	0	-4.047610	-4.068697	-0.966664
31	6	0	-2.876202	-3.325166	-0.604693
32	6	0	-2.697691	-2.330800	-1.620690
33	6	0	-4.830574	-4.627614	0.020121
34	6	0	-4.513367	-4.357786	1.412449
35	6	0	-5.775443	-4.385334	2.115993
36	6	0	-5.964912	-3.583848	3.228439
37	6	0	-4.888119	-2.729220	3.658774
38	6	0	-5.490239	-1.554825	4.243305
39	6	0	-4.831322	-0.349316	4.094756
40	6	0	-5.554541	0.833702	3.684022
41	6	0	-6.924920	0.755539	3.460526
42	6	0	-7.456845	1.398776	2.286562
43	6	0	-6.601405	2.106029	1.418533
44	6	0	-6.871128	2.011125	-0.016731
45	6	0	-7.900041	1.178010	-0.459985
46	6	0	-7.691576	0.337007	-1.617862
47	6	0	-8.454582	-0.869378	-1.425941
48	6	0	-7.939192	-2.068886	-1.887705
49	6	0	-8.066756	-3.265125	-1.066516
50	6	0	-6.868032	-4.049252	-1.254308
51	6	0	-6.271672	-4.681965	-0.148273
52	6	0	-6.863502	-4.555672	1.158885
53	6	0	-8.061132	-3.853249	1.332139
54	6	0	-8.256951	-3.016815	2.501877
55	6	0	-7.229714	-2.906423	3.453722
56	6	0	-6.929927	-1.632771	4.059092
57	6	0	-7.638488	-0.487201	3.675617

58	6	0	-8.666333	-0.586957	2.658414
59	6	0	-8.556025	0.586654	1.813081
60	6	0	-8.779777	0.479395	0.444119
61	6	0	-9.125459	-0.794797	-0.141216
62	6	0	-9.228825	-1.933080	0.661297
63	6	0	-8.675012	-3.190357	0.191713
64	6	0	-8.980528	-1.830714	2.091153
65	6	0	-5.680578	4.725279	-0.633329
66	6	0	-5.867335	5.356895	-1.870456
67	6	0	-6.649675	6.506301	-1.922793
68	6	0	-7.219756	6.990242	-0.744317
69	6	0	-6.977972	6.289117	0.435821
70	6	0	-4.624021	1.681145	-4.365332
71	6	0	-5.752781	2.389733	-4.799255
72	6	0	-5.954785	2.556556	-6.166117
73	6	0	-5.025047	2.017425	-7.055892
74	6	0	-3.928358	1.338790	-6.525349
75	16	0	-1.185447	-1.403292	2.589158
76	8	0	-0.804861	2.000398	-1.896711
77	8	0	-3.399399	3.814978	1.330748
78	8	0	-2.231914	1.625279	2.709060
79	7	0	-6.229946	5.181167	0.497973
80	7	0	-3.723511	1.170810	-5.213744
81	6	0	3.343667	2.268081	2.975317
82	6	0	3.335723	3.139993	1.969633
83	6	0	4.365100	3.192310	0.866408
84	6	0	3.803787	2.671014	-0.478006
85	6	0	2.547020	2.081512	-0.623063
86	6	0	1.340532	1.821404	0.254224
87	6	0	0.819149	0.393378	-0.094500
88	6	0	1.540614	0.068433	-1.391014
89	6	0	4.374393	1.946630	-2.775317
90	6	0	1.838166	-1.136174	-2.005650
91	6	0	2.885874	-1.245801	-3.019184
92	6	0	3.748046	-2.433292	-3.137582
93	6	0	3.743562	-3.442756	-2.059332
94	6	0	2.795317	-3.337130	-0.957468
95	6	0	3.219170	-3.551331	0.351575
96	6	0	2.863519	-2.690239	1.566219
97	6	0	4.209444	-2.476212	2.266073
98	6	0	4.734438	-1.275888	2.791829
99	6	0	3.867485	-0.174390	3.280348
100	6	0	4.453864	1.285814	3.236296
101	6	0	5.560455	1.383750	2.209922
102	6	0	5.553932	2.283838	1.163945
103	6	0	6.584343	2.223827	0.152556
104	6	0	6.223953	2.439934	-1.247116
105	6	0	4.785084	2.515857	-1.549496
106	6	0	2.323362	1.208420	-1.734180
107	6	0	7.117692	1.951573	-2.232236
108	6	0	6.638872	1.367907	-3.473602
109	6	0	5.277575	1.329031	-3.704391
110	6	0	4.655819	0.099311	-4.175222
111	6	0	3.377236	-0.027405	-3.541101
112	6	0	3.159413	1.189271	-2.816003
113	6	0	5.418116	-1.026558	-4.399314
114	6	0	4.951786	-2.307996	-3.895106
115	6	0	6.142990	-3.070370	-3.598640
116	6	0	6.135493	-3.993398	-2.566345
117	6	0	4.924573	-4.174863	-1.809445
118	6	0	5.316345	-4.520026	-0.462272
119	6	0	4.519348	-4.080953	0.576676
120	6	0	5.106466	-3.445622	1.736414
121	6	0	6.487594	-3.315147	1.813280
122	6	0	7.025135	-2.054403	2.262078
123	6	0	6.156041	-1.007438	2.632119
124	6	0	6.567579	0.355407	2.286830
125	6	0	7.735288	0.546319	1.545067
126	6	0	7.744939	1.505454	0.461741
127	6	0	8.637837	1.009140	-0.553189
128	6	0	8.324308	1.224243	-1.885080
129	6	0	8.532687	0.154158	-2.851361
130	6	0	7.465731	0.230371	-3.819421
131	6	0	6.862904	-0.954076	-4.286321
132	6	0	7.319796	-2.231846	-3.804817
133	6	0	8.397066	-2.313121	-2.915865
134	6	0	8.386046	-3.283352	-1.835673
135	6	0	7.277176	-4.133741	-1.679888
136	6	0	6.764375	-4.430697	-0.367021
137	6	0	7.339495	-3.836599	0.763897
138	6	0	8.451941	-2.920743	0.605360
139	6	0	8.258756	-1.833622	1.545102
140	6	0	8.618548	-0.535392	1.189368
141	6	0	9.176742	-0.265512	-0.113107
142	6	0	9.358810	-1.302651	-1.032044
143	6	0	9.015045	-1.090584	-2.424625
144	6	0	8.975683	-2.659801	-0.669583
145	6	0	5.084020	1.587410	4.615422
146	6	0	5.322435	2.912384	5.006400
147	6	0	5.940051	3.148025	6.230438
148	6	0	6.299818	2.059284	7.026485
149	6	0	6.021661	0.779677	6.549430
150	6	0	4.802492	4.663445	0.741616
151	6	0	5.839256	5.175787	1.534202
152	6	0	6.151122	6.528328	1.437781
153	6	0	5.418059	7.328168	0.559979
154	6	0	4.398502	6.727048	-0.176957
155	16	0	1.194058	-2.637695	-1.283919
156	8	0	0.773650	2.560593	1.025703
157	8	0	2.749472	-0.353503	3.725225
158	8	0	1.792528	-2.168598	1.799199
159	7	0	5.430327	0.540692	5.372709
160	7	0	4.090205	5.427308	-0.094461
161	1	0	-2.956359	4.287879	-1.189594
162	1	0	-2.561134	2.847134	-3.092021
163	1	0	-1.054917	0.958480	0.759866
164	1	0	-5.402225	4.947411	-2.760331
165	1	0	-6.811223	7.017124	-2.867999
166	1	0	-7.834530	7.884668	-0.737440
167	1	0	-7.403265	6.628570	1.378477
168	1	0	-6.453453	2.789411	-4.074307
169	1	0	-6.824990	3.095315	-6.530657
170	1	0	-5.143208	2.118987	-8.130016
171	1	0	-3.175541	0.907244	-7.182684
172	1	0	2.519520	2.240941	3.679775
173	1	0	2.501681	3.819680	1.850923
174	1	0	1.164613	-0.270488	0.708858
175	1	0	5.021227	3.727541	4.357834
176	1	0	6.136440	4.164870	6.559002
177	1	0	6.781014	2.195600	7.989775
178	1	0	6.284119	-0.098898	7.135942
179	1	0	6.386126	4.518227	2.201408
180	1	0	6.954089	6.950887	2.035398
181	1	0	5.627424	8.387415	0.449075
182	1	0	3.797395	7.314490	-0.868848

The total electronic energy was calculated to be –7119.81681 Hartree.

Supplementary References

1. Sheldrick, G. M. SHELXT – Integrated space-group and crystal-structure determination. *Acta Crystallogr. A* **71**, 3–8 (2015).
