# Supplementary material for: Synthesis of inter-[60]fullerene conjugates with inherent chirality
Source: Nat Commun. 2024 Jan 15;15:514. doi: 10.1038/s41467-024-44834-x (PMC10789730; doi:10.1038/s41467-024-44834-x)

checkCIF/PLATON report

Structure factors have been supplied for datablock(s) 1Dis17_a

THIS REPORT IS FOR GUIDANCE ONLY. IF USED AS PART OF A REVIEW PROCEDURE FOR PUBLICATION, IT SHOULD NOT REPLACE THE EXPERTISE OF AN EXPERIENCED CRYSTALLOGRAPHIC REFEREE.

No syntax errors found. CIF dictionary Interpreting this report

Datablock: 1Dis17_a

```
Bond precision:   C-C = 0.0064 A          Wavelength=0.71073

Cell:            a=14.482(2)       b=20.564(3)       c=23.960(3)
                 alpha=68.906(1)   beta=78.266(2)    gamma=80.022(2)
Temperature:     100 K
```

	Calculated	Reported
Volume	6479.0(15)	6479.0(15)
Space group	P -1	P -1
Hall group	-P 1	-P 1
Moiety formula	C164 H52 N4 O6 S2, C0.26 H0.10 Cl0.30 S0.33, 5.22(C H Cl3), 0.5	?
Sum formula	C171.48 H57.84 Ar0.62 Cl17.51 N6.12 O6 S5.28	C171.48 H57.84 Ar0.62 Cl17.51 N6.13 O6 S5.28
Mr	3114.25	3114.49
Dx,g cm-3	1.596	1.596
Z	2	2
Mu (mm-1)	0.541	0.541
F000	3141.7	3142.0
F000'	3149.43	
h,k,lmax	17,24,28	17,24,28
Nref	22755	22596
Tmin,Tmax	0.896,0.932	0.791,0.932
Tmin'	0.784	

```
Correction method= # Reported T Limits: Tmin=0.791 Tmax=0.932
AbsCorr = EMPIRICAL

Data completeness= 0.993          Theta(max)= 24.969
```

R(reflections)= 0.0736(17711)

wR2(reflections)= 0.2133(22596)

S = 1.036   Npar= 2176

The following ALERTS were generated. Each ALERT has the format
test-name_ALERT_alert-type_alert-level.
Click on the hyperlinks for more details of the test.

Alert level B

```
PLAT234_ALERT_4_B Large Hirshfeld Difference C124     --C165     .        0.29 Ang.
PLAT245_ALERT_2_B U(iso) H183      Smaller than U(eq) C175      by       0.052 Ang**2
```

Alert level C

```
PLAT041_ALERT_1_C Calc. and Reported SumFormula    Strings  Differ     Please Check
PLAT077_ALERT_4_C Unitcell Contains Non-integer Number of Atoms ..     Please Check
PLAT213_ALERT_2_C Atom C53              has ADP max/min Ratio .....       3.5 oblate
PLAT213_ALERT_2_C Atom C54              has ADP max/min Ratio .....       3.4 prolat
PLAT213_ALERT_2_C Atom C62              has ADP max/min Ratio .....       3.7 oblate
PLAT213_ALERT_2_C Atom C63              has ADP max/min Ratio .....       3.2 prolat
PLAT220_ALERT_2_C NonSolvent   Resd 1  C   Ueq(max)/Ueq(min) Range       4.9 Ratio
PLAT222_ALERT_3_C NonSolvent Resd 1  H   Uiso(max)/Uiso(min) Range       5.4 Ratio
PLAT242_ALERT_2_C Low   'MainMol' Ueq as Compared to Neighbors of       C70 Check
PLAT242_ALERT_2_C Low   'MainMol' Ueq as Compared to Neighbors of       C79 Check
PLAT242_ALERT_2_C Low   'MainMol' Ueq as Compared to Neighbors of      C152 Check
PLAT244_ALERT_4_C Low   'Solvent' Ueq as Compared to Neighbors of      C165 Check
PLAT244_ALERT_4_C Low   'Solvent' Ueq as Compared to Neighbors of      C166 Check
PLAT245_ALERT_2_C U(iso) H182      Smaller than U(eq) C176      by      0.019 Ang**2
PLAT250_ALERT_2_C Large U3/U1 Ratio for Average U(i,j) Tensor ....       2.4 Note
PLAT260_ALERT_2_C Large Average Ueq of Residue Including      Cl42      0.117 Check
PLAT260_ALERT_2_C Large Average Ueq of Residue Including      Cl10      0.119 Check
PLAT260_ALERT_2_C Large Average Ueq of Residue Including      Cl16      0.141 Check
PLAT260_ALERT_2_C Large Average Ueq of Residue Including      Cl32      0.185 Check
PLAT260_ALERT_2_C Large Average Ueq of Residue Including      Cl11      0.103 Check
PLAT260_ALERT_2_C Large Average Ueq of Residue Including       S16      0.130 Check
PLAT340_ALERT_3_C Low Bond Precision on  C-C Bonds ..............    0.00636 Ang.
PLAT369_ALERT_2_C Long   C(sp2)-C(sp2) Bond  C5       - C6       .       1.53 Ang.
PLAT369_ALERT_2_C Long   C(sp2)-C(sp2) Bond  C6       - C7       .       1.54 Ang.
PLAT369_ALERT_2_C Long   C(sp2)-C(sp2) Bond  C16      - C17      .       1.56 Ang.
PLAT369_ALERT_2_C Long   C(sp2)-C(sp2) Bond  C88      - C89      .       1.54 Ang.
PLAT369_ALERT_2_C Long   C(sp2)-C(sp2) Bond  C96      - C97      .       1.53 Ang.
PLAT369_ALERT_2_C Long   C(sp2)-C(sp2) Bond  C97      - C98      .       1.55 Ang.
PLAT906_ALERT_3_C Large K Value in the Analysis of Variance ......      2.056 Check
PLAT911_ALERT_3_C Missing FCF Refl Between Thmin & STh/L=    0.594        157 Report
```

Alert level G

```
FORMU01_ALERT_2_G  There is a discrepancy between the atom counts in the
            _chemical_formula_sum and the formula from the _atom_site* data.
            Atom count from _chemical_formula_sum:C171.48 H57.84 Ar0.62 Cl17.51 N6
            Atom count from the _atom_site data:  C171.4780 H57.83699 Ar0.617 Cl17
PLAT002_ALERT_2_G Number of Distance or Angle Restraints on AtSite         36 Note
PLAT003_ALERT_2_G Number of Uiso or Uij Restrained non-H Atoms ...         45 Report
PLAT004_ALERT_5_G Polymeric Structure Found with Maximum Dimension          1 Info
```

```
PLAT066_ALERT_1_G Predicted and Reported Tmin&Tmax Range Identical          ? Check
PLAT068_ALERT_1_G Reported F000 Differs from Calcd (or Missing)...     Please Check
PLAT083_ALERT_2_G SHELXL Second Parameter in WGHT  Unusually Large      22.04 Why ?
PLAT093_ALERT_1_G No s.u.'s on H-positions, Refinement Reported as      mixed Check
PLAT172_ALERT_4_G The CIF-Embedded .res File Contains DFIX Records         40 Report
PLAT178_ALERT_4_G The CIF-Embedded .res File Contains SIMU Records          8 Report
PLAT302_ALERT_4_G Anion/Solvent/Minor-Residue Disorder (Resd  2  )       100% Note
PLAT302_ALERT_4_G Anion/Solvent/Minor-Residue Disorder (Resd  3  )        75% Note
PLAT302_ALERT_4_G Anion/Solvent/Minor-Residue Disorder (Resd  6  )       100% Note
PLAT302_ALERT_4_G Anion/Solvent/Minor-Residue Disorder (Resd  7  )       100% Note
PLAT302_ALERT_4_G Anion/Solvent/Minor-Residue Disorder (Resd  8  )       100% Note
PLAT302_ALERT_4_G Anion/Solvent/Minor-Residue Disorder (Resd  9  )       100% Note
PLAT302_ALERT_4_G Anion/Solvent/Minor-Residue Disorder (Resd 10  )       100% Note
PLAT302_ALERT_4_G Anion/Solvent/Minor-Residue Disorder (Resd 11  )       100% Note
PLAT302_ALERT_4_G Anion/Solvent/Minor-Residue Disorder (Resd 12  )       100% Note
PLAT302_ALERT_4_G Anion/Solvent/Minor-Residue Disorder (Resd 13  )       100% Note
PLAT302_ALERT_4_G Anion/Solvent/Minor-Residue Disorder (Resd 14  )       100% Note
PLAT302_ALERT_4_G Anion/Solvent/Minor-Residue Disorder (Resd 15  )       100% Note
PLAT302_ALERT_4_G Anion/Solvent/Minor-Residue Disorder (Resd 16  )       100% Note
PLAT302_ALERT_4_G Anion/Solvent/Minor-Residue Disorder (Resd 17  )       100% Note
PLAT302_ALERT_4_G Anion/Solvent/Minor-Residue Disorder (Resd 18  )       100% Note
PLAT302_ALERT_4_G Anion/Solvent/Minor-Residue Disorder (Resd 19  )       100% Note
PLAT302_ALERT_4_G Anion/Solvent/Minor-Residue Disorder (Resd 20  )       100% Note
PLAT343_ALERT_2_G Unusual sp?      Angle Range in Main Residue for        C28 Check
PLAT343_ALERT_2_G Unusual sp?      Angle Range in Main Residue for        C29 Check
PLAT343_ALERT_2_G Unusual sp?      Angle Range in Main Residue for        C30 Check
PLAT343_ALERT_2_G Unusual sp?      Angle Range in Main Residue for        C38 Check
PLAT343_ALERT_2_G Unusual sp?      Angle Range in Main Residue for        C41 Check
PLAT343_ALERT_2_G Unusual sp?      Angle Range in Main Residue for        C49 Check
PLAT343_ALERT_2_G Unusual sp?      Angle Range in Main Residue for        C50 Check
PLAT343_ALERT_2_G Unusual sp?      Angle Range in Main Residue for        C51 Check
PLAT343_ALERT_2_G Unusual sp?      Angle Range in Main Residue for        C52 Check
PLAT343_ALERT_2_G Unusual sp?      Angle Range in Main Residue for        C54 Check
PLAT343_ALERT_2_G Unusual sp?      Angle Range in Main Residue for        C55 Check
PLAT343_ALERT_2_G Unusual sp?      Angle Range in Main Residue for        C56 Check
PLAT343_ALERT_2_G Unusual sp?      Angle Range in Main Residue for        C57 Check
PLAT343_ALERT_2_G Unusual sp?      Angle Range in Main Residue for        C58 Check
PLAT343_ALERT_2_G Unusual sp?      Angle Range in Main Residue for        C59 Check
PLAT343_ALERT_2_G Unusual sp?      Angle Range in Main Residue for        C61 Check
PLAT343_ALERT_2_G Unusual sp?      Angle Range in Main Residue for        C62 Check
PLAT343_ALERT_2_G Unusual sp?      Angle Range in Main Residue for        C64 Check
PLAT343_ALERT_2_G Unusual sp?      Angle Range in Main Residue for       C112 Check
PLAT343_ALERT_2_G Unusual sp?      Angle Range in Main Residue for       C122 Check
PLAT343_ALERT_2_G Unusual sp?      Angle Range in Main Residue for       C125 Check
PLAT343_ALERT_2_G Unusual sp?      Angle Range in Main Residue for       C130 Check
PLAT343_ALERT_2_G Unusual sp?      Angle Range in Main Residue for       C131 Check
PLAT343_ALERT_2_G Unusual sp?      Angle Range in Main Residue for       C132 Check
PLAT343_ALERT_2_G Unusual sp?      Angle Range in Main Residue for       C133 Check
PLAT343_ALERT_2_G Unusual sp?      Angle Range in Main Residue for       C134 Check
PLAT343_ALERT_2_G Unusual sp?      Angle Range in Main Residue for       C135 Check
PLAT343_ALERT_2_G Unusual sp?      Angle Range in Main Residue for       C139 Check
PLAT343_ALERT_2_G Unusual sp?      Angle Range in Main Residue for       C140 Check
PLAT343_ALERT_2_G Unusual sp?      Angle Range in Main Residue for       C141 Check
PLAT343_ALERT_2_G Unusual sp?      Angle Range in Main Residue for       C142 Check
PLAT343_ALERT_2_G Unusual sp?      Angle Range in Main Residue for       C143 Check
PLAT343_ALERT_2_G Unusual sp?      Angle Range in Main Residue for       C144 Check
PLAT343_ALERT_2_G Unusual sp?      Angle Range in Main Residue for       C145 Check
```

```
PLAT343_ALERT_2_G Unusual sp?      Angle Range in Main Residue for       C146 Check
PLAT431_ALERT_2_G Short Inter HL..A Contact  Cl9      ..S15      .       3.38 Ang.
                                              -1+x,y,z  =     1_455 Check
PLAT432_ALERT_2_G Short Inter X...Y Contact  Ar1      ..C12      .       3.36 Ang.
                                                 x,y,z  =     1_555 Check
PLAT432_ALERT_2_G Short Inter X...Y Contact  Ar1      ..C13      .       3.37 Ang.
                                                 x,y,z  =     1_555 Check
PLAT432_ALERT_2_G Short Inter X...Y Contact  Ar2      ..C93      .       3.38 Ang.
                                                 x,y,z  =     1_555 Check
PLAT432_ALERT_2_G Short Inter X...Y Contact  Cl15     ..C76      .       3.21 Ang.
                                                 x,y,z  =     1_555 Check
PLAT432_ALERT_2_G Short Inter X...Y Contact  Cl15     ..C77      .       3.23 Ang.
                                                 x,y,z  =     1_555 Check
PLAT432_ALERT_2_G Short Inter X...Y Contact  Cl19     ..C155     .       3.19 Ang.
                                                 x,y,z  =     1_555 Check
PLAT432_ALERT_2_G Short Inter X...Y Contact  S14      ..C27      .       3.23 Ang.
                                                 x,y,z  =     1_555 Check
PLAT432_ALERT_2_G Short Inter X...Y Contact  S15      ..C73      .       3.11 Ang.
                                               1+x,y,z  =     1_655 Check
PLAT432_ALERT_2_G Short Inter X...Y Contact  O6       ..C166     .       3.01 Ang.
                                                 x,y,z  =     1_555 Check
PLAT432_ALERT_2_G Short Inter X...Y Contact  O8       ..C166     .       3.02 Ang.
                                                 x,y,z  =     1_555 Check
PLAT432_ALERT_2_G Short Inter X...Y Contact  C31      ..C141     .       3.06 Ang.
                                           1-x,1-y,1-z  =     2_666 Check
PLAT432_ALERT_2_G Short Inter X...Y Contact  C36      ..C36      .       3.11 Ang.
                                            -x,2-y,1-z  =     2_576 Check
PLAT432_ALERT_2_G Short Inter X...Y Contact  C63      ..C116     .       3.02 Ang.
                                               x,1+y,z  =     1_565 Check
PLAT432_ALERT_2_G Short Inter X...Y Contact  C63      ..C136     .       3.13 Ang.
                                               x,1+y,z  =     1_565 Check
PLAT432_ALERT_2_G Short Inter X...Y Contact  C108     ..C110     .       3.19 Ang.
                                            -x,1-y,1-z  =     2_566 Check
PLAT432_ALERT_2_G Short Inter X...Y Contact  C110     ..C110     .       2.99 Ang.
                                            -x,1-y,1-z  =     2_566 Check
PLAT780_ALERT_1_G Coordinates do not Form a Properly Connected Set     Please Do !
PLAT860_ALERT_3_G Number of Least-Squares Restraints ............        529 Note
PLAT883_ALERT_1_G No Info/Value for _atom_sites_solution_primary .     Please Do !
PLAT909_ALERT_3_G Percentage of I>2sig(I) Data at Theta(Max) Still       58% Note
PLAT910_ALERT_3_G Missing # of FCF Reflection(s) Below Theta(Min).         4 Note
PLAT933_ALERT_2_G Number of HKL-OMIT Records in Embedded .res File        11 Note
PLAT941_ALERT_3_G Average HKL Measurement Multiplicity ..........        2.7 Low
PLAT960_ALERT_3_G Number of Intensities with I  <   - 2*sig(I) ...        76 Check
PLAT965_ALERT_2_G The SHELXL WEIGHT Optimisation has not Converged     Please Check
PLAT967_ALERT_5_G Note: Two-Theta Cutoff Value in Embedded .res ..      51.0 Degree
PLAT978_ALERT_2_G Number C-C Bonds with Positive Residual Density.         1 Info
```

---

0 **ALERT level A** = Most likely a serious problem - resolve or explain
2 **ALERT level B** = A potentially serious problem, consider carefully
30 **ALERT level C** = Check. Ensure it is not caused by an omission or oversight
90 **ALERT level G** = General information/check it is not something unexpected

6 ALERT type 1 CIF construction/syntax error, inconsistent or missing data

```
82 ALERT type 2 Indicator that the structure model may be wrong or deficient
 9 ALERT type 3 Indicator that the structure quality may be low
23 ALERT type 4 Improvement, methodology, query or suggestion
 2 ALERT type 5 Informative message, check
```

It is advisable to attempt to resolve as many as possible of the alerts in all categories. Often the minor alerts point to easily fixed oversights, errors and omissions in your CIF or refinement strategy, so attention to these fine details can be worthwhile. In order to resolve some of the more serious problems it may be necessary to carry out additional measurements or structure refinements. However, the purpose of your study may justify the reported deviations and the more serious of these should normally be commented upon in the discussion or experimental section of a paper or in the "special_details" fields of the CIF. checkCIF was carefully designed to identify outliers and unusual parameters, but every test has its limitations and alerts that are not important in a particular case may appear. Conversely, the absence of alerts does not guarantee there are no aspects of the results needing attention. It is up to the individual to critically assess their own results and, if necessary, seek expert advice.

Publication of your CIF in IUCr journals

A basic structural check has been run on your CIF. These basic checks will be run on all CIFs submitted for publication in IUCr journals (*Acta Crystallographica*, *Journal of Applied Crystallography*, *Journal of Synchrotron Radiation*); however, if you intend to submit to *Acta Crystallographica Section C* or *E* or *IUCrData*, you should make sure that full publication checks are run on the final version of your CIF prior to submission.

Publication of your CIF in other journals

Please refer to the *Notes for Authors* of the relevant journal for any special instructions relating to CIF submission.

PLATON version of 18/05/2022; check.def file version of 17/05/2022

Datablock 1Dis17_a - ellipsoid plot

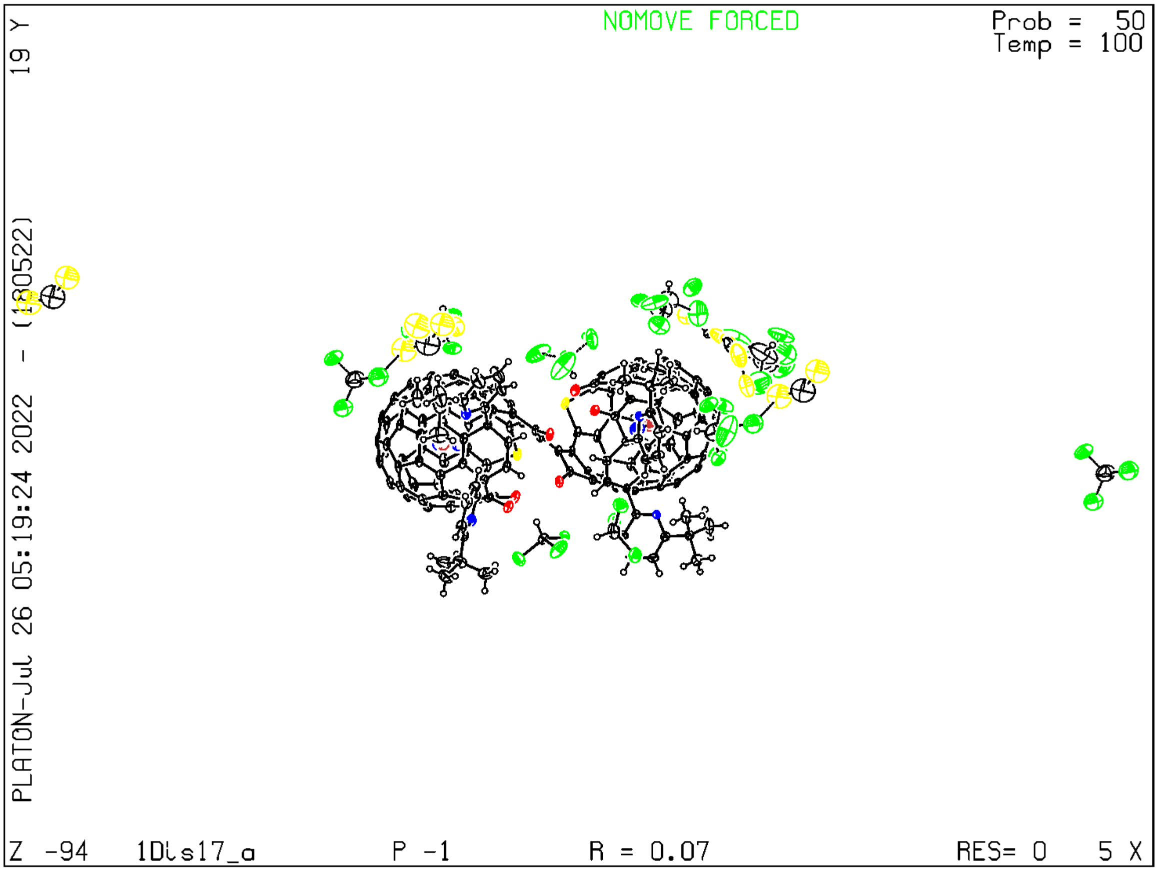

Supplement: Supplementary file 4 — Source data [file 41467_2024_44834_MOESM4_ESM.zip › Source Data/Compd4_checkcif.pdf]
