# Supplementary material for: Synthesis of inter-[60]fullerene conjugates with inherent chirality
Source: Nat Commun. 2024 Jan 15;15:514. doi: 10.1038/s41467-024-44834-x (PMC10789730; doi:10.1038/s41467-024-44834-x)

checkCIF/PLATON report

You have not supplied any structure factors. As a result the full set of tests cannot be run.

THIS REPORT IS FOR GUIDANCE ONLY. IF USED AS PART OF A REVIEW PROCEDURE FOR PUBLICATION, IT SHOULD NOT REPLACE THE EXPERTISE OF AN EXPERIENCED CRYSTALLOGRAPHIC REFEREE.

No syntax errors found. CIF dictionary Interpreting this report

Datablock: 2sp3PP_a

Bond precision: C-C = 0.0107 A Wavelength=0.71073

Cell:	a=19.364(2)	b=24.487(3)	c=28.535(3)
	alpha=104.498(1)	beta=90.616(2)	gamma=102.812(1)
Temperature:	100 K		

	Calculated	Reported
Volume	12742(2)	12741(3)
Space group	P -1	P -1
Hall group	-P 1	-P 1
Moiety formula	2(C164 H54 N4 O6 S2), 8.5(C7 H8), 0.504(C6 H5), 2.805(C S2), C0	?
Sum formula	C394.40 H188.02 N8 O16 S10.46	C394.39 H188.01 N8 O16 S10.46
Mr	5629.57	5629.52
Dx,g cm-3	1.467	1.467
Z	2	2
Mu (mm-1)	0.171	0.171
F000	5811.4	5811.0
F000'	5816.32	
h,k,lmax		23,29,33
Nref		42951
Tmin,Tmax	0.929,0.978	0.799,0.978
Tmin'	0.791	

Correction method= # Reported T Limits: Tmin=0.799 Tmax=0.978
AbsCorr = EMPIRICAL

Data completeness= Theta(max)= 25.025

R(reflections)= 0.1252(32178) wR2(reflections)= 0.2893(42951)

S = 1.176 Npar= 3925

The following ALERTS were generated. Each ALERT has the format
test-name_ALERT_alert-type_alert-level.
Click on the hyperlinks for more details of the test.

Alert level B

```
CRYSS02_ALERT_3_B  The value of _exptl_crystal_size_max is > 1.0
   Maximum crystal size given =   1.370
PLAT005_ALERT_5_B No Embedded Refinement Details Found  in the CIF     Please Do !
PLAT029_ALERT_3_B _diffrn_measured_fraction_theta_full value Low .      0.955 Why?
```

Author Response: Due to the fragility of the crystal caused by a continuous release of cocrystalized solvent molecules during measurements, collection at higher resolutions and better completeness may be possible only using a synchrotron which enables short-time measurements.

```
PLAT097_ALERT_2_B Large Reported Max.  (Positive) Residual Density       1.75 eA-3
```

Author Response: Due to the severe disorders of solvent molecules, large density still remained though they were solved using appropriate models with 11 (for CS2) and 14 (for toluene) dispositions.

```
PLAT213_ALERT_2_B Atom C215            has ADP max/min Ratio .....         4.3 oblate
PLAT220_ALERT_2_B NonSolvent   Resd 1  C   Ueq(max)/Ueq(min) Range       10.0 Ratio
```

Author Response: This is due to the severe disorders of solvent molecules but not by main structures.

```
PLAT221_ALERT_2_B Solv./Anion  Resd 2  C   Ueq(max)/Ueq(min) Range       10.0 Ratio
```

Author Response: This is due to the severe disorders of solvent molecules and inevitable for the crystal of this fullerene compound because many solvent molecules are filled in relatively large voids generated among fullerene cages.

```
PLAT242_ALERT_2_B Low    'MainMol' Ueq as Compared to Neighbors of        C70 Check
```

Author Response: This is due to the high flexibility of the t-butyl group where C70 is a quaternary carbon atom.

PLAT332_ALERT_2_B Large Phenyl C-C Range   C366   -C371   .   0.35 Ang.

Author Response: This is due to the severe disorders of toluene molecules and inevitable for the crystal of this fullerene compound.

PLAT340_ALERT_3_B Low Bond Precision on C-C Bonds ....   0.01073 Ang.

Author Response: This is due to the severe disorders of solvent molecules and inevitable for the crystal of this fullerene compound because many solvent molecules are filled in relatively large voids generated among fullerene cages.

PLAT420_ALERT_2_B D-H Bond Without Acceptor O13   --H13   .   Please Check

Author Response: The encapsulated H2O molecule inside fullerenes behaves like a gaseous single water molecule in vacuo without definitive interactions with H-bonding acceptors as discussed in ChemPhysChem 2017, 18, 1229-1233. Therefore, the absence of acceptors for the protons are reasonable.

PLAT420_ALERT_2_B D-H Bond Without Acceptor O13   --H13A   .   Please Check

Author Response: The encapsulated H2O molecule inside fullerenes behaves like a gaseous single water molecule in vacuo without definitive interactions with H-bonding acceptors as discussed in ChemPhysChem 2017, 18, 1229-1233. Therefore, the absence of acceptors for the protons are reasonable.

PLAT420_ALERT_2_B D-H Bond Without Acceptor O14   --H14   .   Please Check

Author Response: The encapsulated H2O molecule inside fullerenes behaves like a gaseous single water molecule in vacuo without definitive interactions with H-bonding acceptors as discussed in ChemPhysChem 2017, 18, 1229-1233. Therefore, the absence of acceptors for the protons are reasonable.

PLAT420_ALERT_2_B D-H Bond Without Acceptor O14   --H14A   .   Please Check

Author Response: The encapsulated H2O molecule inside fullerenes behaves like a gaseous single water molecule in vacuo without definitive interactions with H-bonding acceptors as discussed in ChemPhysChem 2017, 18, 1229-1233. Therefore, the absence of acceptors for the protons are reasonable.

PLAT420_ALERT_2_B D-H Bond Without Acceptor O15   --H15   .   Please Check

Author Response: The encapsulated H2O molecule inside fullerenes behaves like a gaseous single water molecule in vacuo without definitive interactions with H-bonding acceptors as discussed in ChemPhysChem 2017, 18, 1229-1233. Therefore, the absence of acceptors for the protons are reasonable.

```
PLAT420_ALERT_2_B D-H Bond Without Acceptor  O15      --H15A     .     Please Check
```

Author Response: The encapsulated H2O molecule inside fullerenes behaves like a gaseous single water molecule in vacuo without definitive interactions with H-bonding acceptors as discussed in ChemPhysChem 2017, 18, 1229-1233. Therefore, the absence of acceptors for the protons are reasonable.

```
PLAT420_ALERT_2_B D-H Bond Without Acceptor  O16      --H16      .     Please Check
```

Author Response: The encapsulated H2O molecule inside fullerenes behaves like a gaseous single water molecule in vacuo without definitive interactions with H-bonding acceptors as discussed in ChemPhysChem 2017, 18, 1229-1233. Therefore, the absence of acceptors for the protons are reasonable.

```
PLAT420_ALERT_2_B D-H Bond Without Acceptor  O16      --H16A     .     Please Check
```

Author Response: The encapsulated H2O molecule inside fullerenes behaves like a gaseous single water molecule in vacuo without definitive interactions with H-bonding acceptors as discussed in ChemPhysChem 2017, 18, 1229-1233. Therefore, the absence of acceptors for the protons are reasonable.

```
PLAT964_ALERT_2_B SHELXL WEIGHT Par. Values in CIF & RES Differ ..     Please Check
```

Alert level C

```
CRYSC01_ALERT_1_C The word below has not been recognised as a standard
            identifier.
            'wine
CRYSC01_ALERT_1_C The word below has not been recognised as a standard
            identifier.
            red'
CRYSC01_ALERT_1_C No recognised colour has been given for crystal colour.
DIFMN02_ALERT_2_C  The minimum difference density is < -0.1*ZMAX*0.75
            _refine_diff_density_min given =     -1.600
            Test value =     -1.200
DIFMN03_ALERT_1_C  The minimum difference density is < -0.1*ZMAX*0.75
            The relevant atom site should be identified.
DIFMX02_ALERT_1_C  The maximum difference density is > 0.1*ZMAX*0.75
            The relevant atom site should be identified.
PLAT041_ALERT_1_C Calc. and Reported SumFormula    Strings  Differ     Please Check
              Calc: C394.40 H188.02 N8 O16 S10.46
              Rep.: C394.39 H188.01 N8 O16 S10.46
PLAT082_ALERT_2_C High R1 Value ................................       0.13 Report
PLAT084_ALERT_3_C High wR2 Value (i.e. > 0.25) ..................       0.29 Report
```

```
PLAT098_ALERT_2_C Large Reported Min.  (Negative) Residual Density      -1.60 eA-3
PLAT202_ALERT_3_C Isotropic non-H Atoms in Anion/Solvent .........         39 Check
              C341    C355    C356    C357    C358    C359    C360    C385
              C387    C388    C389    C390    C391    C392    C394    C401
              C402    C403    C404    C405    C406    C414    C415    C416
              C417    C418    C419    C420    C408    C409    C410    C411
              C412    C413    S5      S6      C346    C383    C407
PLAT213_ALERT_2_C Atom C213             has ADP max/min Ratio .....        3.5 oblate
PLAT213_ALERT_2_C Atom C214             has ADP max/min Ratio .....        3.1 oblate
PLAT213_ALERT_2_C Atom C291             has ADP max/min Ratio .....        3.3 prolat
PLAT213_ALERT_2_C Atom C294             has ADP max/min Ratio .....        3.1 prolat
PLAT213_ALERT_2_C Atom C295             has ADP max/min Ratio .....        3.5 oblate
PLAT213_ALERT_2_C Atom C299             has ADP max/min Ratio .....        3.3 oblate
PLAT213_ALERT_2_C Atom C301             has ADP max/min Ratio .....        3.3 oblate
PLAT213_ALERT_2_C Atom C307             has ADP max/min Ratio .....        3.2 oblate
PLAT213_ALERT_2_C Atom C308             has ADP max/min Ratio .....        3.6 prolat
PLAT213_ALERT_2_C Atom C325             has ADP max/min Ratio .....        3.9 prolat
PLAT214_ALERT_2_C Atom C71        (Anion/Solvent) ADP max/min Ratio        4.2 prolat
PLAT214_ALERT_2_C Atom C84        (Anion/Solvent) ADP max/min Ratio        4.6 oblate
PLAT220_ALERT_2_C NonSolvent   Resd 1  N   Ueq(max)/Ueq(min) Range         3.7 Ratio
```

Author Response: This is due to the severe disorders of solvent molecules but not by main structures.

```
PLAT221_ALERT_2_C Solv./Anion  Resd 9  C   Ueq(max)/Ueq(min) Range         4.2 Ratio
```

Author Response: This is due to the severe disorders of solvent molecules and inevitable for the crystal of this fullerene compound because many solvent molecules are filled in relatively large voids generated among fullerene cages.

```
PLAT221_ALERT_2_C Solv./Anion  Resd10  C   Ueq(max)/Ueq(min) Range         4.6 Ratio
```

Author Response: This is due to the severe disorders of solvent molecules and inevitable for the crystal of this fullerene compound because many solvent molecules are filled in relatively large voids generated among fullerene cages.

```
PLAT222_ALERT_3_C NonSolvent Resd 1  H   Uiso(max)/Uiso(min) Range        10.0 Ratio
PLAT223_ALERT_4_C Solv./Anion  Resd 2  H   Ueq(max)/Ueq(min) Range        10.0 Ratio
PLAT223_ALERT_4_C Solv./Anion  Resd 9  H   Ueq(max)/Ueq(min) Range         5.2 Ratio
PLAT223_ALERT_4_C Solv./Anion  Resd10  H   Ueq(max)/Ueq(min) Range         5.7 Ratio
PLAT223_ALERT_4_C Solv./Anion  Resd28  H   Ueq(max)/Ueq(min) Range        10.0 Ratio
PLAT223_ALERT_4_C Solv./Anion  Resd31  H   Ueq(max)/Ueq(min) Range         6.8 Ratio
PLAT242_ALERT_2_C Low    'MainMol' Ueq as Compared to Neighbors of        C324 Check
```

Author Response: This is due to the high flexibility of the t-butyl group where C70 is a quaternary carbon atom.

```
PLAT242_ALERT_2_C Low    'MainMol' Ueq as Compared to Neighbors of        C79 Check
```

Author Response: This is due to the high flexibility of the t-butyl group where C70 is a quaternary carbon atom.

```
PLAT243_ALERT_4_C High   'Solvent' Ueq as Compared to Neighbors of       C353 Check
PLAT243_ALERT_4_C High   'Solvent' Ueq as Compared to Neighbors of       C355 Check
PLAT244_ALERT_4_C Low    'Solvent' Ueq as Compared to Neighbors of       C329 Check
PLAT244_ALERT_4_C Low    'Solvent' Ueq as Compared to Neighbors of       C339 Check
PLAT244_ALERT_4_C Low    'Solvent' Ueq as Compared to Neighbors of       C340 Check
PLAT244_ALERT_4_C Low    'Solvent' Ueq as Compared to Neighbors of       C356 Check
PLAT245_ALERT_2_C U(iso) H13       Smaller than U(eq) O13       by      0.012 Ang**2
PLAT250_ALERT_2_C Large U3/U1 Ratio for Average U(i,j) Tensor ....        2.9 Note
PLAT250_ALERT_2_C Large U3/U1 Ratio for Average U(i,j) Tensor ....        2.3 Note
PLAT260_ALERT_2_C Large Average Ueq of Residue Including      C339      0.159 Check
PLAT260_ALERT_2_C Large Average Ueq of Residue Including      C386      0.114 Check
PLAT360_ALERT_2_C Short  C(sp3)-C(sp3) Bond  C70      - C73      .       1.38 Ang.
PLAT369_ALERT_2_C Long   C(sp2)-C(sp2) Bond  C174     - C175     .       1.55 Ang.
PLAT369_ALERT_2_C Long   C(sp2)-C(sp2) Bond  C175     - C176     .       1.54 Ang.
PLAT369_ALERT_2_C Long   C(sp2)-C(sp2) Bond  C256     - C257     .       1.55 Ang.
PLAT369_ALERT_2_C Long   C(sp2)-C(sp2) Bond  C11      - C12      .       1.55 Ang.
PLAT369_ALERT_2_C Long   C(sp2)-C(sp2) Bond  C93      - C94      .       1.55 Ang.
PLAT369_ALERT_2_C Long   C(sp2)-C(sp2) Bond  C94      - C95      .       1.53 Ang.
PLAT412_ALERT_2_C Short Intra XH3 .. XHn     H327     ..H330     .       1.85 Ang.
                                                      x,y,z  =      1_555 Check
```

Alert level G

```
PLAT002_ALERT_2_G Number of Distance or Angle Restraints on AtSite         56 Note
PLAT003_ALERT_2_G Number of Uiso or Uij Restrained non-H Atoms ...         62 Report
PLAT063_ALERT_4_G Crystal Size Possibly too Large for Beam Size ..       1.37 mm
PLAT066_ALERT_1_G Predicted and Reported Tmin&Tmax Range Identical          ? Check
PLAT068_ALERT_1_G Reported F000 Differs from Calcd (or Missing)...     Please Check
PLAT083_ALERT_2_G SHELXL Second Parameter in WGHT  Unusually Large     107.62 Why ?
PLAT301_ALERT_3_G Main Residue  Disorder .............(Resd  1  )         8% Note
PLAT302_ALERT_4_G Anion/Solvent/Minor-Residue Disorder (Resd  2  )         2% Note
PLAT302_ALERT_4_G Anion/Solvent/Minor-Residue Disorder (Resd  4  )       100% Note
PLAT302_ALERT_4_G Anion/Solvent/Minor-Residue Disorder (Resd  7  )       100% Note
PLAT302_ALERT_4_G Anion/Solvent/Minor-Residue Disorder (Resd  8  )       100% Note
PLAT302_ALERT_4_G Anion/Solvent/Minor-Residue Disorder (Resd  9  )       100% Note
PLAT302_ALERT_4_G Anion/Solvent/Minor-Residue Disorder (Resd 10  )       100% Note
PLAT302_ALERT_4_G Anion/Solvent/Minor-Residue Disorder (Resd 11  )       100% Note
PLAT302_ALERT_4_G Anion/Solvent/Minor-Residue Disorder (Resd 12  )       100% Note
PLAT302_ALERT_4_G Anion/Solvent/Minor-Residue Disorder (Resd 13  )       100% Note
PLAT302_ALERT_4_G Anion/Solvent/Minor-Residue Disorder (Resd 14  )       100% Note
PLAT302_ALERT_4_G Anion/Solvent/Minor-Residue Disorder (Resd 15  )       100% Note
PLAT302_ALERT_4_G Anion/Solvent/Minor-Residue Disorder (Resd 16  )       100% Note
PLAT302_ALERT_4_G Anion/Solvent/Minor-Residue Disorder (Resd 17  )       100% Note
PLAT302_ALERT_4_G Anion/Solvent/Minor-Residue Disorder (Resd 18  )       100% Note
PLAT302_ALERT_4_G Anion/Solvent/Minor-Residue Disorder (Resd 19  )       100% Note
PLAT302_ALERT_4_G Anion/Solvent/Minor-Residue Disorder (Resd 20  )       100% Note
PLAT302_ALERT_4_G Anion/Solvent/Minor-Residue Disorder (Resd 21  )       100% Note
PLAT302_ALERT_4_G Anion/Solvent/Minor-Residue Disorder (Resd 22  )       100% Note
PLAT302_ALERT_4_G Anion/Solvent/Minor-Residue Disorder (Resd 23  )       100% Note
PLAT302_ALERT_4_G Anion/Solvent/Minor-Residue Disorder (Resd 24  )       100% Note
```

```
PLAT302_ALERT_4_G Anion/Solvent/Minor-Residue Disorder (Resd 25  )       100% Note
PLAT302_ALERT_4_G Anion/Solvent/Minor-Residue Disorder (Resd 26  )       100% Note
PLAT302_ALERT_4_G Anion/Solvent/Minor-Residue Disorder (Resd 27  )       100% Note
PLAT302_ALERT_4_G Anion/Solvent/Minor-Residue Disorder (Resd 32  )       100% Note
PLAT304_ALERT_4_G Non-Integer Number of Atoms in ..... (Resd  4  )      12.32 Check
PLAT304_ALERT_4_G Non-Integer Number of Atoms in ..... (Resd  7  )       8.64 Check
PLAT304_ALERT_4_G Non-Integer Number of Atoms in ..... (Resd  8  )      11.87 Check
PLAT304_ALERT_4_G Non-Integer Number of Atoms in ..... (Resd  9  )      12.32 Check
PLAT304_ALERT_4_G Non-Integer Number of Atoms in ..... (Resd 10  )       8.40 Check
PLAT304_ALERT_4_G Non-Integer Number of Atoms in ..... (Resd 11  )       6.94 Check
PLAT304_ALERT_4_G Non-Integer Number of Atoms in ..... (Resd 12  )       3.22 Check
PLAT304_ALERT_4_G Non-Integer Number of Atoms in ..... (Resd 13  )       7.44 Check
PLAT304_ALERT_4_G Non-Integer Number of Atoms in ..... (Resd 14  )       6.60 Check
PLAT304_ALERT_4_G Non-Integer Number of Atoms in ..... (Resd 15  )       4.73 Check
PLAT304_ALERT_4_G Non-Integer Number of Atoms in ..... (Resd 16  )       5.54 Check
PLAT304_ALERT_4_G Non-Integer Number of Atoms in ..... (Resd 17  )       1.61 Check
PLAT304_ALERT_4_G Non-Integer Number of Atoms in ..... (Resd 18  )       0.63 Check
PLAT304_ALERT_4_G Non-Integer Number of Atoms in ..... (Resd 19  )       0.54 Check
PLAT304_ALERT_4_G Non-Integer Number of Atoms in ..... (Resd 20  )       1.27 Check
PLAT304_ALERT_4_G Non-Integer Number of Atoms in ..... (Resd 21  )       1.42 Check
PLAT304_ALERT_4_G Non-Integer Number of Atoms in ..... (Resd 22  )       0.60 Check
PLAT304_ALERT_4_G Non-Integer Number of Atoms in ..... (Resd 23  )       0.63 Check
PLAT304_ALERT_4_G Non-Integer Number of Atoms in ..... (Resd 24  )       0.54 Check
PLAT304_ALERT_4_G Non-Integer Number of Atoms in ..... (Resd 25  )       0.90 Check
PLAT304_ALERT_4_G Non-Integer Number of Atoms in ..... (Resd 26  )       0.81 Check
PLAT304_ALERT_4_G Non-Integer Number of Atoms in ..... (Resd 27  )       0.90 Check
PLAT304_ALERT_4_G Non-Integer Number of Atoms in ..... (Resd 32  )       2.02 Check
PLAT315_ALERT_2_G Singly Bonded Carbon Detected (H-atoms Missing).       C383 Check
PLAT343_ALERT_2_G Unusual sp?      Angle Range in Main Residue for       C194 Check
PLAT343_ALERT_2_G Unusual sp?      Angle Range in Main Residue for       C197 Check
PLAT343_ALERT_2_G Unusual sp?      Angle Range in Main Residue for       C205 Check
PLAT343_ALERT_2_G Unusual sp?      Angle Range in Main Residue for       C206 Check
PLAT343_ALERT_2_G Unusual sp?      Angle Range in Main Residue for       C212 Check
PLAT343_ALERT_2_G Unusual sp?      Angle Range in Main Residue for       C213 Check
PLAT343_ALERT_2_G Unusual sp?      Angle Range in Main Residue for       C214 Check
PLAT343_ALERT_2_G Unusual sp?      Angle Range in Main Residue for       C215 Check
PLAT343_ALERT_2_G Unusual sp?      Angle Range in Main Residue for       C216 Check
PLAT343_ALERT_2_G Unusual sp?      Angle Range in Main Residue for       C217 Check
PLAT343_ALERT_2_G Unusual sp?      Angle Range in Main Residue for       C218 Check
PLAT343_ALERT_2_G Unusual sp?      Angle Range in Main Residue for       C219 Check
PLAT343_ALERT_2_G Unusual sp?      Angle Range in Main Residue for       C220 Check
PLAT343_ALERT_2_G Unusual sp?      Angle Range in Main Residue for       C222 Check
PLAT343_ALERT_2_G Unusual sp?      Angle Range in Main Residue for       C223 Check
PLAT343_ALERT_2_G Unusual sp?      Angle Range in Main Residue for       C224 Check
PLAT343_ALERT_2_G Unusual sp?      Angle Range in Main Residue for       C225 Check
PLAT343_ALERT_2_G Unusual sp?      Angle Range in Main Residue for       C226 Check
PLAT343_ALERT_2_G Unusual sp?      Angle Range in Main Residue for       C227 Check
PLAT343_ALERT_2_G Unusual sp?      Angle Range in Main Residue for       C276 Check
PLAT343_ALERT_2_G Unusual sp?      Angle Range in Main Residue for       C279 Check
PLAT343_ALERT_2_G Unusual sp?      Angle Range in Main Residue for       C281 Check
PLAT343_ALERT_2_G Unusual sp?      Angle Range in Main Residue for       C284 Check
PLAT343_ALERT_2_G Unusual sp?      Angle Range in Main Residue for       C285 Check
PLAT343_ALERT_2_G Unusual sp?      Angle Range in Main Residue for       C288 Check
PLAT343_ALERT_2_G Unusual sp?      Angle Range in Main Residue for       C290 Check
PLAT343_ALERT_2_G Unusual sp?      Angle Range in Main Residue for       C295 Check
PLAT343_ALERT_2_G Unusual sp?      Angle Range in Main Residue for       C296 Check
PLAT343_ALERT_2_G Unusual sp?      Angle Range in Main Residue for       C297 Check
```

```
PLAT343_ALERT_2_G Unusual sp?      Angle Range in Main Residue for       C298 Check
PLAT343_ALERT_2_G Unusual sp?      Angle Range in Main Residue for       C300 Check
PLAT343_ALERT_2_G Unusual sp?      Angle Range in Main Residue for       C302 Check
PLAT343_ALERT_2_G Unusual sp?      Angle Range in Main Residue for       C303 Check
PLAT343_ALERT_2_G Unusual sp?      Angle Range in Main Residue for       C304 Check
PLAT343_ALERT_2_G Unusual sp?      Angle Range in Main Residue for       C305 Check
PLAT343_ALERT_2_G Unusual sp?      Angle Range in Main Residue for       C306 Check
PLAT343_ALERT_2_G Unusual sp?      Angle Range in Main Residue for       C307 Check
PLAT343_ALERT_2_G Unusual sp?      Angle Range in Main Residue for       C308 Check
PLAT343_ALERT_2_G Unusual sp?      Angle Range in Main Residue for       C309 Check
PLAT343_ALERT_2_G Unusual sp?      Angle Range in Main Residue for        C31 Check
PLAT343_ALERT_2_G Unusual sp?      Angle Range in Main Residue for        C34 Check
PLAT343_ALERT_2_G Unusual sp?      Angle Range in Main Residue for        C36 Check
PLAT343_ALERT_2_G Unusual sp?      Angle Range in Main Residue for        C39 Check
PLAT343_ALERT_2_G Unusual sp?      Angle Range in Main Residue for        C42 Check
PLAT343_ALERT_2_G Unusual sp?      Angle Range in Main Residue for        C43 Check
PLAT343_ALERT_2_G Unusual sp?      Angle Range in Main Residue for        C45 Check
PLAT343_ALERT_2_G Unusual sp?      Angle Range in Main Residue for        C50 Check
PLAT343_ALERT_2_G Unusual sp?      Angle Range in Main Residue for        C51 Check
PLAT343_ALERT_2_G Unusual sp?      Angle Range in Main Residue for        C52 Check
PLAT343_ALERT_2_G Unusual sp?      Angle Range in Main Residue for        C53 Check
PLAT343_ALERT_2_G Unusual sp?      Angle Range in Main Residue for        C54 Check
PLAT343_ALERT_2_G Unusual sp?      Angle Range in Main Residue for        C55 Check
PLAT343_ALERT_2_G Unusual sp?      Angle Range in Main Residue for        C56 Check
PLAT343_ALERT_2_G Unusual sp?      Angle Range in Main Residue for        C58 Check
PLAT343_ALERT_2_G Unusual sp?      Angle Range in Main Residue for        C59 Check
PLAT343_ALERT_2_G Unusual sp?      Angle Range in Main Residue for        C61 Check
PLAT343_ALERT_2_G Unusual sp?      Angle Range in Main Residue for        C62 Check
PLAT343_ALERT_2_G Unusual sp?      Angle Range in Main Residue for        C63 Check
PLAT343_ALERT_2_G Unusual sp?      Angle Range in Main Residue for        C64 Check
PLAT343_ALERT_2_G Unusual sp?      Angle Range in Main Residue for       C113 Check
PLAT343_ALERT_2_G Unusual sp?      Angle Range in Main Residue for       C115 Check
PLAT343_ALERT_2_G Unusual sp?      Angle Range in Main Residue for       C116 Check
PLAT343_ALERT_2_G Unusual sp?      Angle Range in Main Residue for       C117 Check
PLAT343_ALERT_2_G Unusual sp?      Angle Range in Main Residue for       C121 Check
PLAT343_ALERT_2_G Unusual sp?      Angle Range in Main Residue for       C122 Check
PLAT343_ALERT_2_G Unusual sp?      Angle Range in Main Residue for       C123 Check
PLAT343_ALERT_2_G Unusual sp?      Angle Range in Main Residue for       C124 Check
PLAT343_ALERT_2_G Unusual sp?      Angle Range in Main Residue for       C125 Check
PLAT343_ALERT_2_G Unusual sp?      Angle Range in Main Residue for       C130 Check
PLAT343_ALERT_2_G Unusual sp?      Angle Range in Main Residue for       C133 Check
PLAT343_ALERT_2_G Unusual sp?      Angle Range in Main Residue for       C135 Check
PLAT343_ALERT_2_G Unusual sp?      Angle Range in Main Residue for       C136 Check
PLAT343_ALERT_2_G Unusual sp?      Angle Range in Main Residue for       C137 Check
PLAT343_ALERT_2_G Unusual sp?      Angle Range in Main Residue for       C140 Check
PLAT343_ALERT_2_G Unusual sp?      Angle Range in Main Residue for       C144 Check
PLAT343_ALERT_2_G Unusual sp?      Angle Range in Main Residue for       C146 Check
PLAT380_ALERT_4_G Incorrectly? Oriented X(sp2)-Methyl Moiety .....       C394 Check
PLAT380_ALERT_4_G Incorrectly? Oriented X(sp2)-Methyl Moiety .....       C420 Check
PLAT411_ALERT_2_G Short Inter H...H Contact  H473     ..H491     .       1.61 Ang.
                                               1-x,1-y,1-z  =      2_666 Check
PLAT412_ALERT_2_G Short Intra XH3 .. XHn     H150     ..H385     .       2.11 Ang.
                                                     x,y,z  =      1_555 Check
PLAT413_ALERT_2_G Short Inter XH3 .. XHn     H71A     ..H501     .       1.77 Ang.
                                                 -x,-y,2-z  =      2_557 Check
PLAT413_ALERT_2_G Short Inter XH3 .. XHn     H73A     ..H465     .       2.12 Ang.
                                                     x,y,z  =      1_555 Check
```

```
PLAT413_ALERT_2_G Short Inter XH3 .. XHn     H82A     ..H426     .       2.05 Ang.
                                                -1+x,y,z  =     1_455 Check
PLAT413_ALERT_2_G Short Inter XH3 .. XHn     H380     ..H446     .       2.11 Ang.
                                             1-x,1-y,1-z  =     2_666 Check
PLAT432_ALERT_2_G Short Inter X...Y Contact  S3       ..C380     .       3.26 Ang.
                                              1-x,-y,1-z  =     2_656 Check
PLAT432_ALERT_2_G Short Inter X...Y Contact  S22      ..C316     .       3.05 Ang.
                                                x,-1+y,z  =     1_545 Check
PLAT432_ALERT_2_G Short Inter X...Y Contact  S22      ..C141     .       3.29 Ang.
                                              1+x,-1+y,z  =     1_645 Check
PLAT432_ALERT_2_G Short Inter X...Y Contact  C15      ..C301     .       3.19 Ang.
                                                   x,y,z  =     1_555 Check
PLAT432_ALERT_2_G Short Inter X...Y Contact  C58      ..C436     .       3.19 Ang.
                                             -1+x,-1+y,z  =     1_445 Check
PLAT432_ALERT_2_G Short Inter X...Y Contact  C126     ..C333     .       3.17 Ang.
                                                   x,y,z  =     1_555 Check
PLAT432_ALERT_2_G Short Inter X...Y Contact  C174     ..C174     .       3.14 Ang.
                                              1-x,-y,1-z  =     2_656 Check
PLAT432_ALERT_2_G Short Inter X...Y Contact  C174     ..C175     .       3.15 Ang.
                                              1-x,-y,1-z  =     2_656 Check
PLAT432_ALERT_2_G Short Inter X...Y Contact  C353     ..C389     .       3.18 Ang.
                                             1-x,1-y,1-z  =     2_666 Check
PLAT722_ALERT_1_G Angle   Calc      108.00, Rep      109.50 Dev...       1.50 Degree
              H407    -C407    -H408       1_555  1_555  1_555     # 1412 Check
PLAT773_ALERT_2_G Check long C-C Bond in CIF: C407     --C408            1.82 Ang.
PLAT790_ALERT_4_G Centre of Gravity not Within Unit Cell: Resd.  #          8 Note
              C7 H8
PLAT790_ALERT_4_G Centre of Gravity not Within Unit Cell: Resd.  #         12 Note
              C7 H8
PLAT790_ALERT_4_G Centre of Gravity not Within Unit Cell: Resd.  #         14 Note
              C7 H8
PLAT790_ALERT_4_G Centre of Gravity not Within Unit Cell: Resd.  #         15 Note
              C7 H8
PLAT790_ALERT_4_G Centre of Gravity not Within Unit Cell: Resd.  #         16 Note
              C6 H5
PLAT790_ALERT_4_G Centre of Gravity not Within Unit Cell: Resd.  #         18 Note
              C S2
PLAT790_ALERT_4_G Centre of Gravity not Within Unit Cell: Resd.  #         22 Note
              C S2
PLAT790_ALERT_4_G Centre of Gravity not Within Unit Cell: Resd.  #         23 Note
              C S2
PLAT790_ALERT_4_G Centre of Gravity not Within Unit Cell: Resd.  #         25 Note
              C S2
PLAT790_ALERT_4_G Centre of Gravity not Within Unit Cell: Resd.  #         32 Note
              C H3
PLAT860_ALERT_3_G Number of Least-Squares Restraints ............        333 Note
PLAT883_ALERT_1_G No Info/Value for _atom_sites_solution_primary .     Please Do !
PLAT899_ALERT_4_G SHELXL2018 is Deprecated and Succeeded by SHELXL     2019/3 Note
```

---

0 **ALERT level A** = Most likely a serious problem - resolve or explain
19 **ALERT level B** = A potentially serious problem, consider carefully
53 **ALERT level C** = Check. Ensure it is not caused by an omission or oversight
163 **ALERT level G** = General information/check it is not something unexpected

10 ALERT type 1 CIF construction/syntax error, inconsistent or missing data

```
144 ALERT type 2 Indicator that the structure model may be wrong or deficient
  8 ALERT type 3 Indicator that the structure quality may be low
 72 ALERT type 4 Improvement, methodology, query or suggestion
  1 ALERT type 5 Informative message, check
```

It is advisable to attempt to resolve as many as possible of the alerts in all categories. Often the minor alerts point to easily fixed oversights, errors and omissions in your CIF or refinement strategy, so attention to these fine details can be worthwhile. In order to resolve some of the more serious problems it may be necessary to carry out additional measurements or structure refinements. However, the purpose of your study may justify the reported deviations and the more serious of these should normally be commented upon in the discussion or experimental section of a paper or in the "special_details" fields of the CIF. checkCIF was carefully designed to identify outliers and unusual parameters, but every test has its limitations and alerts that are not important in a particular case may appear. Conversely, the absence of alerts does not guarantee there are no aspects of the results needing attention. It is up to the individual to critically assess their own results and, if necessary, seek expert advice.

Publication of your CIF in IUCr journals

A basic structural check has been run on your CIF. These basic checks will be run on all CIFs submitted for publication in IUCr journals (*Acta Crystallographica*, *Journal of Applied Crystallography*, *Journal of Synchrotron Radiation*); however, if you intend to submit to *Acta Crystallographica Section C* or *E* or *IUCrData*, you should make sure that full publication checks are run on the final version of your CIF prior to submission.

Publication of your CIF in other journals

Please refer to the *Notes for Authors* of the relevant journal for any special instructions relating to CIF submission.

PLATON version of 14/11/2023; check.def file version of 14/09/2023

Datablock 2sp3PP_a - ellipsoid plot

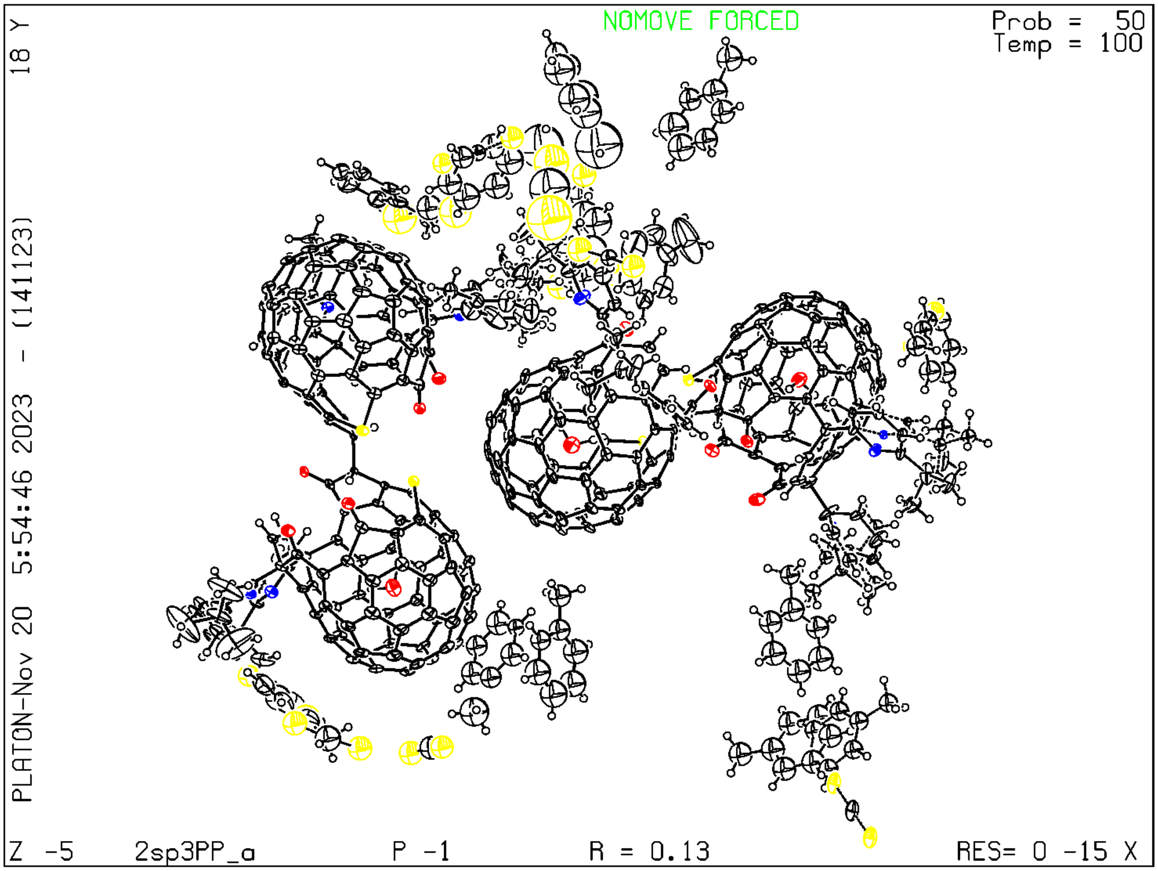

Supplement: Supplementary file 4 — Source data [file 41467_2024_44834_MOESM4_ESM.zip › Source Data/Compd5_checkcif.pdf]
